# Supplementary material for: Bioinformatics leading to conveniently accessible, helix enforcing, bicyclic ASX motif mimics (BAMMs)
Source: Nat Commun. 2024 May 17;15:4217. doi: 10.1038/s41467-024-48323-z (PMC11101637; doi:10.1038/s41467-024-48323-z)
Supplement: Supplementary file 1 — Supplementary Information [file 41467_2024_48323_MOESM1_ESM.pdf]

Supplementary Information

Bioinformatics Leading To Conveniently Accessible, Helix Enforcing, Bicyclic ASX Motif Mimics (BAMMs)

Tianxiong Mi[1], Duyen Nguyen[1], Zhe Gao[1], Kevin Burgess[1]

[1]Department of Chemistry, Texas A & M University, Box 30012, College Station, TX 77842, USA

E-mail: burgess@tamu.edu

Supplementary Notes

TLC: thin layer chromatography

prepHPLC: preparation high performance liquid chromatography

MeCN: acetonitrile

analyHPLC: analytical high performance liquid chromatography

ESI-MS: electrospray ionization mass spectrometry

Fmoc: fluorenylmethoxycarbonyl

DIPEA: N,N-diisopropylethylamine

DMF: dimethylformamide

Oxyma: ethyl cyanohydroxyiminoacetate

DIC: N,N'-diisopropylcarbodiimide

PyBOP: benzotriazol-1-yloxytripyrrolidinophosphonium hexafluorophosphate

HOBt: hydroxybenzotriazole

NMM: N-methylmorpholine

TFA: trifluoroacetic acid

TFE: 2,2,2-trifluoroethanol

TIPS: triisopropylsilane

DMSO: dimethyl sulfoxide

PBS: phosphate-buffered saline

MCMM: Monte Carlo Multiple Minimum

TBMB: 1,3,5-tris(bromomethyl)benzene

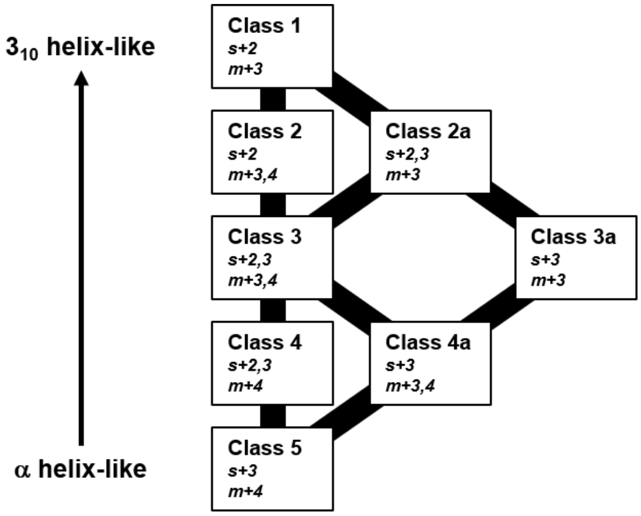

Supplementary Figure 1* Classification of ASX motifs based on *H*-bond patterns.[1] *s+x* refers to *H*-bond between *Ncap* side chain *CO* and *Nx* main chain *NH*, *m+x* refers to *H*-bond between *Ncap* main chain *CO* and *Nx* main chain *NH.*

*Supplementary Figure 1 is reprinted (adapted) from original Figure 3 in A Natural Grouping of Motifs with an Aspartate or Asparagine Residue Forming Two Hydrogen Bonds to Residues Ahead in Sequence: Their Occurrence at α-Helical N Termini and in Other Situations, 286, Wan, W.-Y. and Milner-White, E. J., 1633-1649, Copyright (1999), with permission from Elsevier.

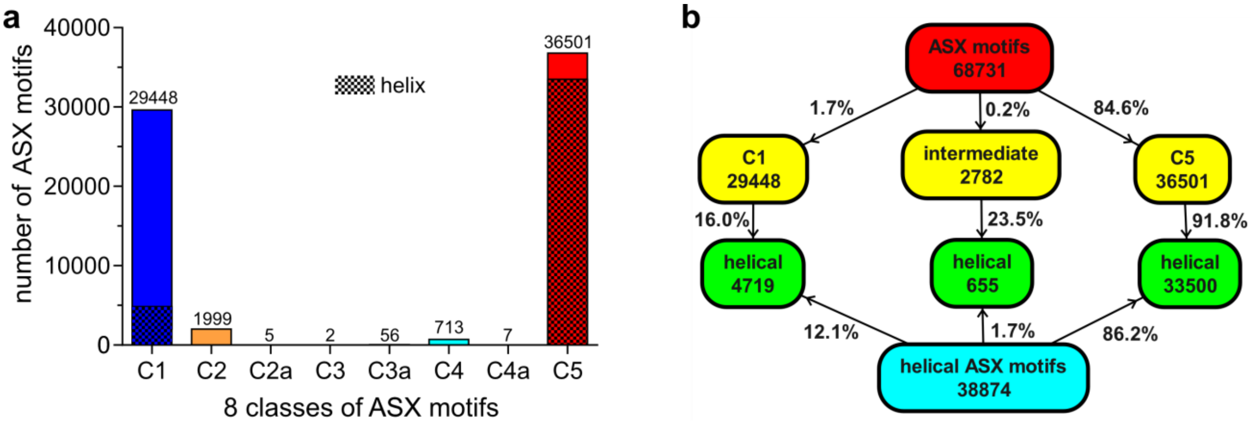

Supplementary Figure 2 ASX Motifs in Different Classes. **a**. number of ASX motifs in different classes. **b**. number of helical ASX motifs in class 1 (C1), intermediate classes (intermediate) and class 5 (C5). Grid lines represent the percentage of motifs at the *N*-terminus of α-helix in each class. 96% motifs were in class 1 and class 5, while helical motifs were mainly in class 5 (86%), with *H*-bond pattern s*Ncap*,m*N3* and m*Ncap*,m*N4*.

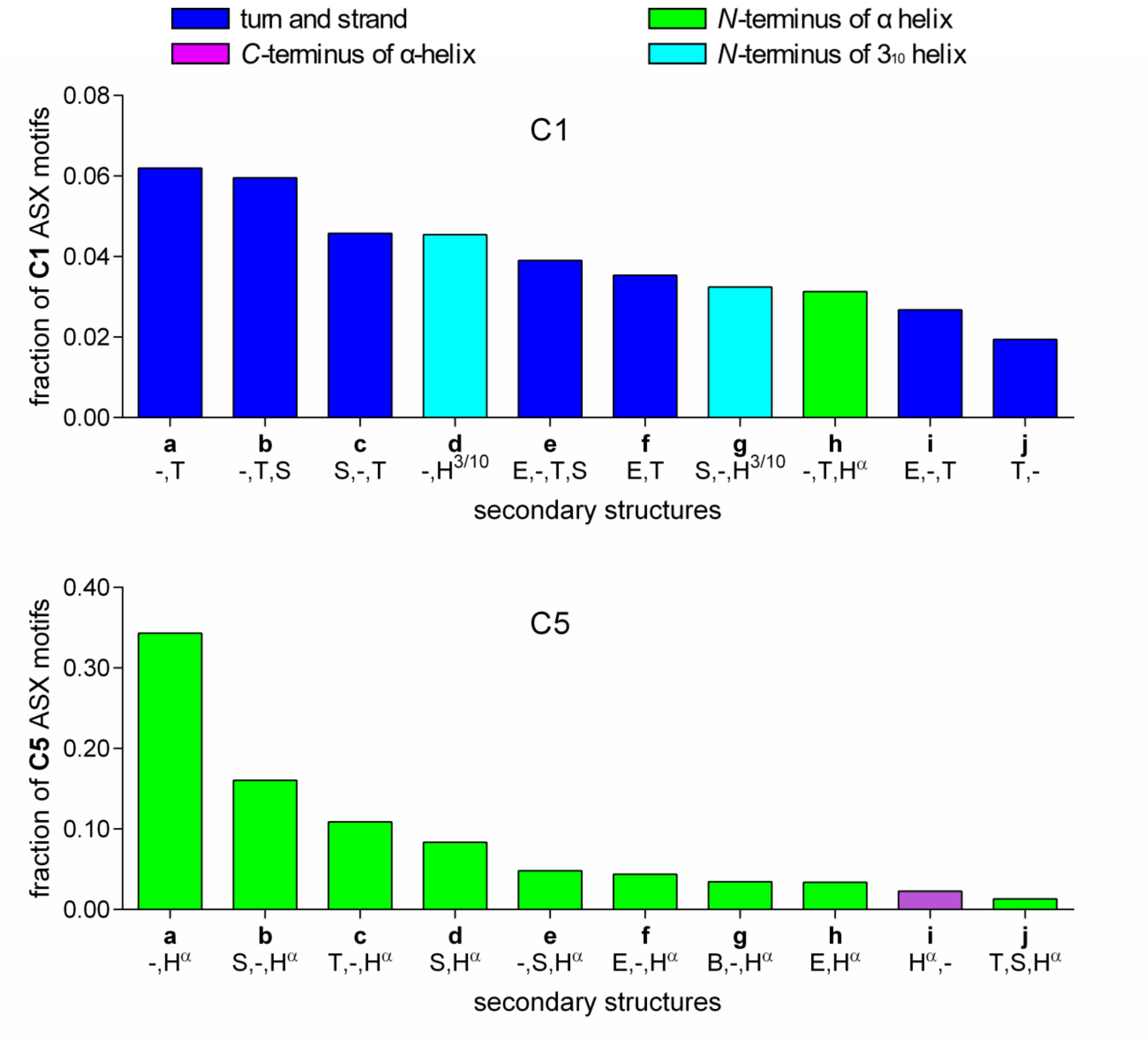

Supplementary Figure 3 Top 10 secondary structures of ASX motifs in C1 and C5. For each motif, *N'*, *Ncap*, *N1*, *N2*, *N3* and *N4* were used in the secondary structure analyses with the help of *DSSP* program[2]. The final result for each motif showed the secondary structure from *N'* to *N5*. Those with same secondary structure combos were combined and divided by total number of motifs in that class to obtain the fraction and make graphics below. ASX motifs in C1 were more loop-like with features of turn and strand, while those in C5 were dominated by helical features

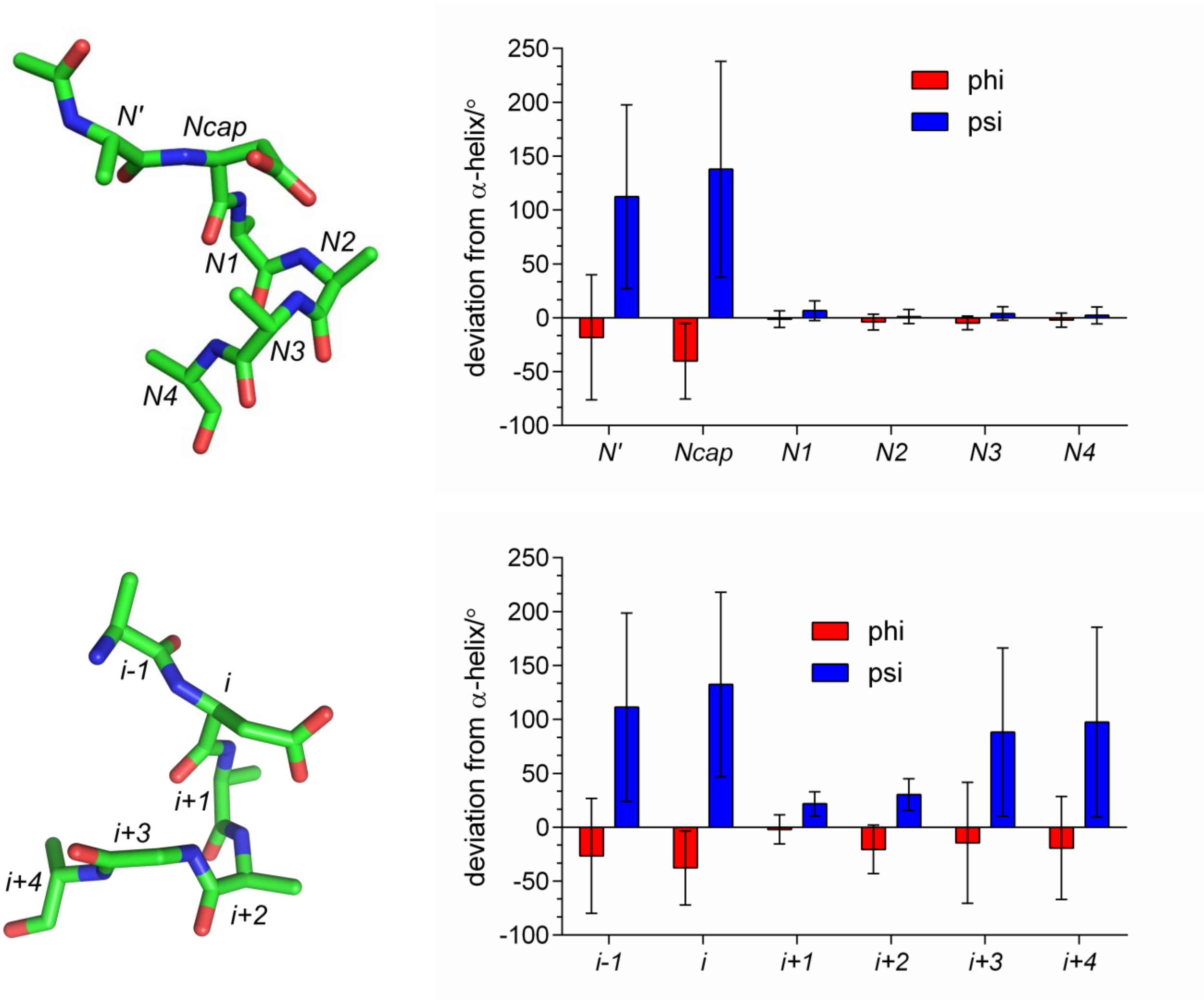

Supplementary Figure 4 Dihedral angles {relative to (-60,-40°)} of helical C5 ASX motifs (top) and C1 ASX motifs. Among 36501 ASX motifs in C5, 33500 motifs (92%) are at the *N*-termini of α-helices. Dihedral angles analyses were made using dihedral angles of these (33500) motifs; error bars refer to S.D. of dihedrals at each position of 33500 ASX motfis. It indicated that they were helix-inducing motifs and helix usually started at *N1*. (-60°, -40°) was used as the dihedral angle for standard α-helix. 29448 motifs are in C1. Dihedral angle analyses were made using these motifs. Significant deviation from *i-1* to *i+4* (except for *i+1*} indicated that they were usually not served as helix-inducing motifs.

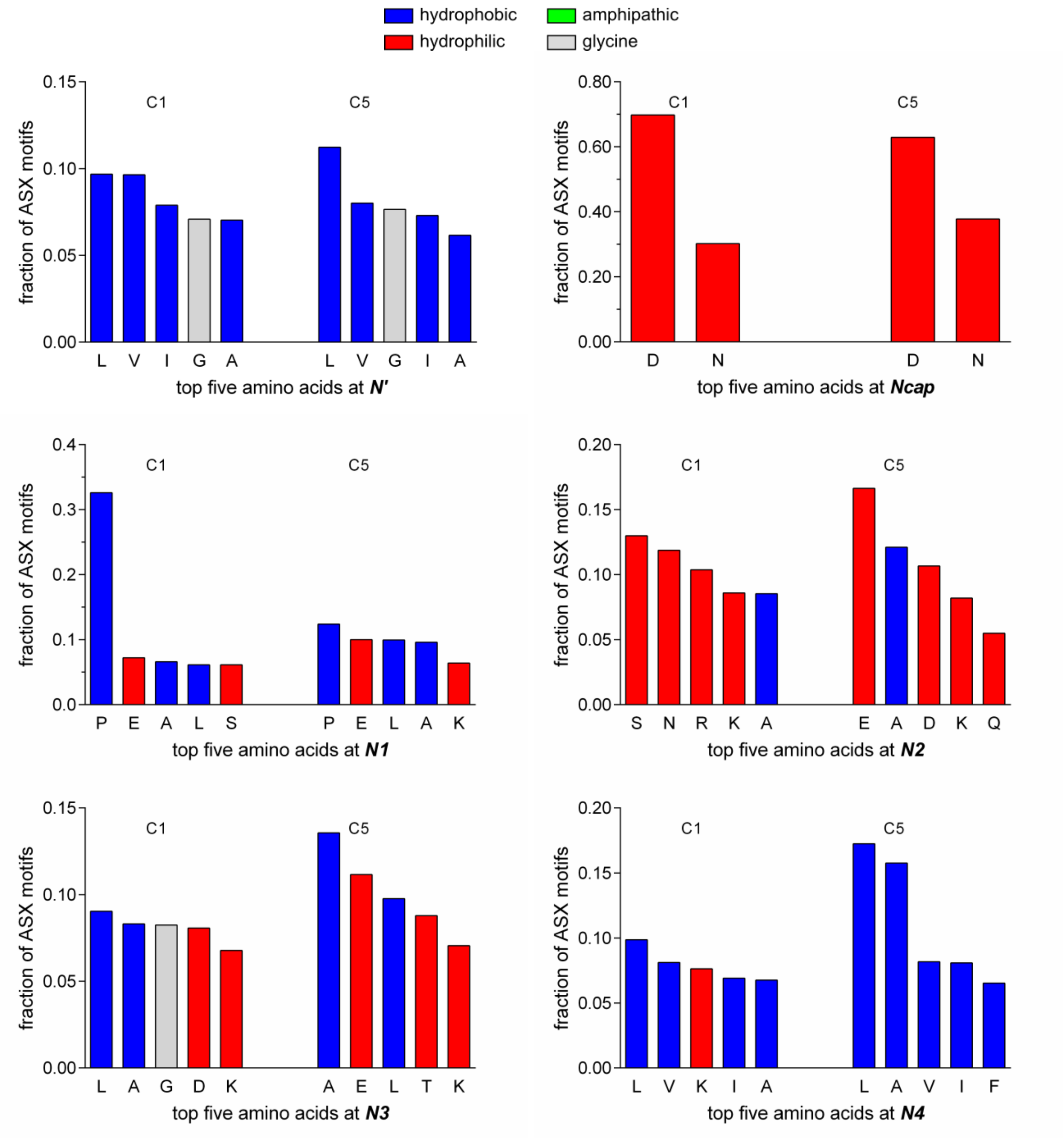

Supplementary Figure 5 Top 5 most abundant residues from *N'* to *N4* in C1 and C5 ASX motifs. For motifs in each class, residues at the same position (*N'*, *Ncap*, *N1*, *N2*, *N3* and *N4*) (in non-helical ASX motifs, their corresponding labels are *i-1*, *i*, *i+1*, *i+2*, *i+3*, *i+4*) were collected, grouped and counted. The numbers of each residue were divided by total number of motifs in the class to obtain fractions. Top five residues at each position were presented and compared in C1 and C5.

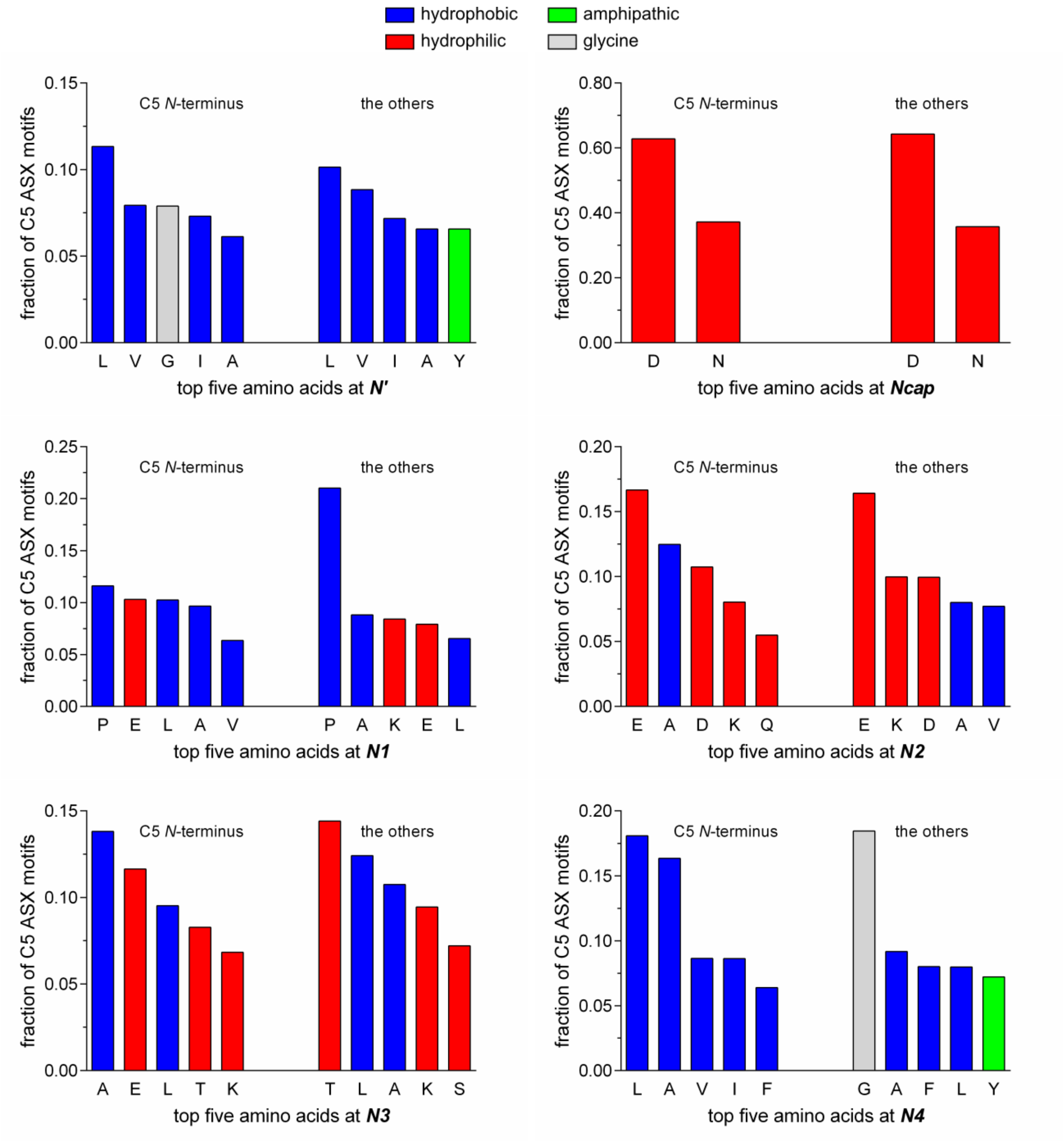

Supplementary Figure 6 Top 5 most abundant residues from *N'* to *N4* in C5 helical and non-helical ASX motifs. All motifs in C5 were separated into two sub-classes: C5-*N*-terminus (92%) and C5-the-others (8%). The former represents those C5 ASX motifs at the *N*-termini of α-helices, which serve as *N*-caps, while the latter are the remaining C5 ASX motifs. Residue in the sub-classes were collected, grouped and analyzed. Top five residues at (*N'*, *Ncap*, *N1*, *N2*, *N3* and *N4*) were presented and compared (in non-helical ASX motifs, their corresponding labels should be *i-1*, *i*, *i+1*, *i+2*, *i+3*, *i+4*, but for simplicity, here we use *N'* to *N4* for both sub-classes).

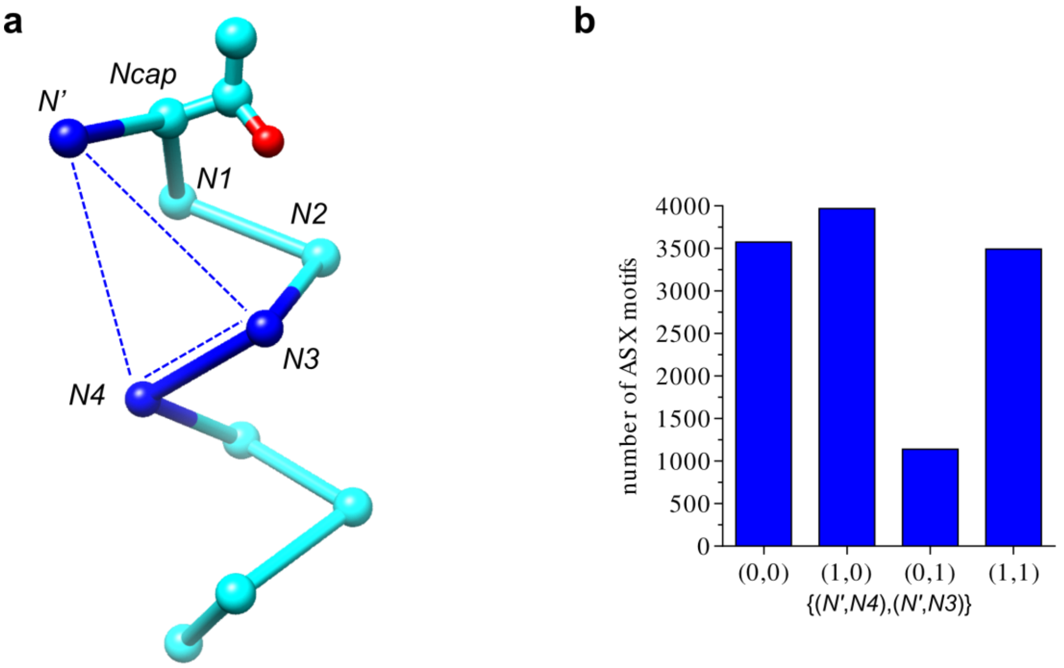

Supplementary Figure 7 Hydrophobic Interactions in *N*-cap ASX Motifs. **a**. interactions between side chains of residues *N'*,*N3* and *N4* to form hydrophobic triangles. **b**. hydrophobic interaction patterns of helical C5 ASX motifs (when *N'* and *N4* are hydrophobic). (0,0) for no hydrophobic interactions; (1,0) for a hydrophobic patch of (*N'*,*N4*); (0,1) for a hydrophobic patch of (*N'*,*N3*); (1,1) for (*N'*,*N4*) and (*N'*,*N3*), *ie* triangle. Among 33500 C5 ASX motifs at helical *N*-cap, 12175 which had hydrophobic residues at *N'* and *N4* were used as dataset in this study. Around 1/3 motifs are unlikely to have hydrophobic interactions. More than 1/2 can have one hydrophobic patch either between *N'* and *N4*, or *N'* and *N3*, and around 1/3 have hydrophobic triangles

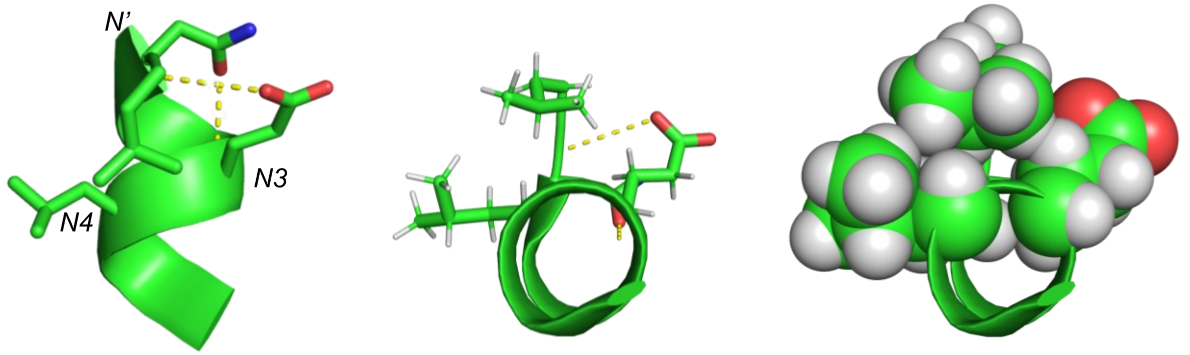

Supplementary Figure 8 An example of ASX motif with both triangle and capping box. The high abundancy of Q and E in (1,1) (see main text Fig. 2g) indicates that the hydrophobic triangle can coexist with capping box to stabilize the helix in proteins. Above is an example from crystal 2F7K.

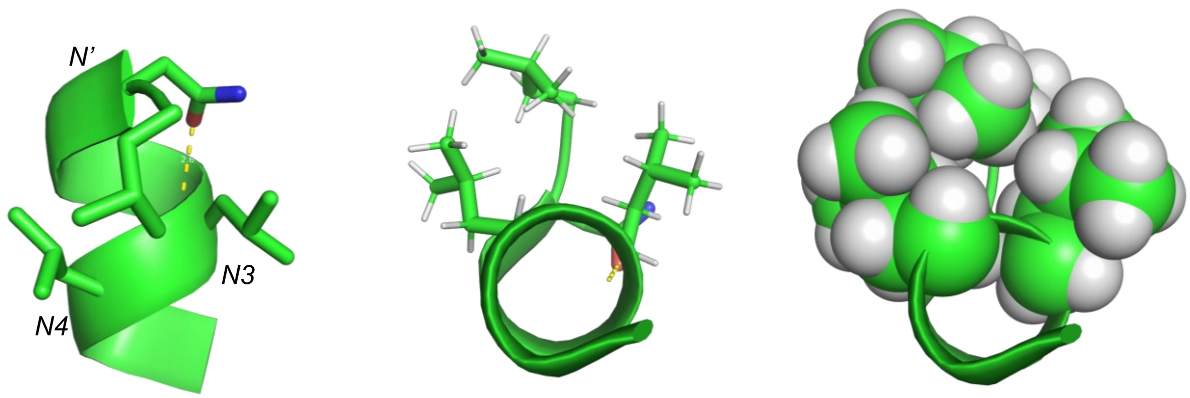

Supplementary Figure 9 An example of ASX motif with hydrophobic triangle with three hydrophobic residues at *N'*,*N3* and *N4*. Hydrophobic residues like L and V can also help form the hydrophobic triangle. Above is an example from crystal 4JW1.

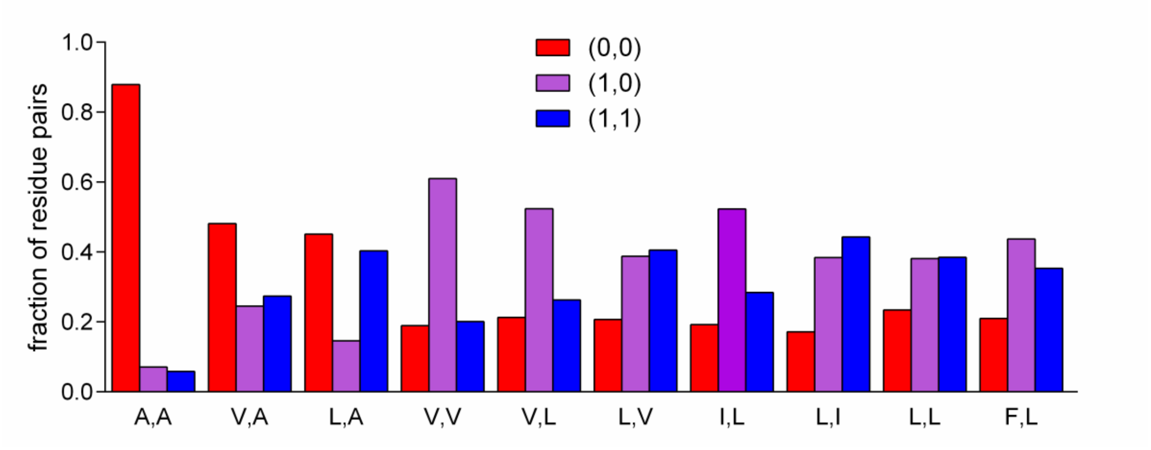

Supplementary Figure 10 Distribution of (*N'*, *N4*) residue pairs of helical ASX motifs in three categories: no interactions (0,0), patches (1,0) and triangles (1,1). Residue pairs (*N'*, *N4*) abundance in different hydrophobic patterns were analyzed. Pairs that occur in more than 250 motifs were considered in this study. Overall, (A,A) at (*N'*,*N4*) is unlikely to form either patch or triangle, and when sidechains are longer, the tendency to form patches for residue pairs becomes dominant (in V,V and V,L), and as they become even longer (L,I and L,L), the tendency to form triangles increases and finally reach a approximately the same level as patches

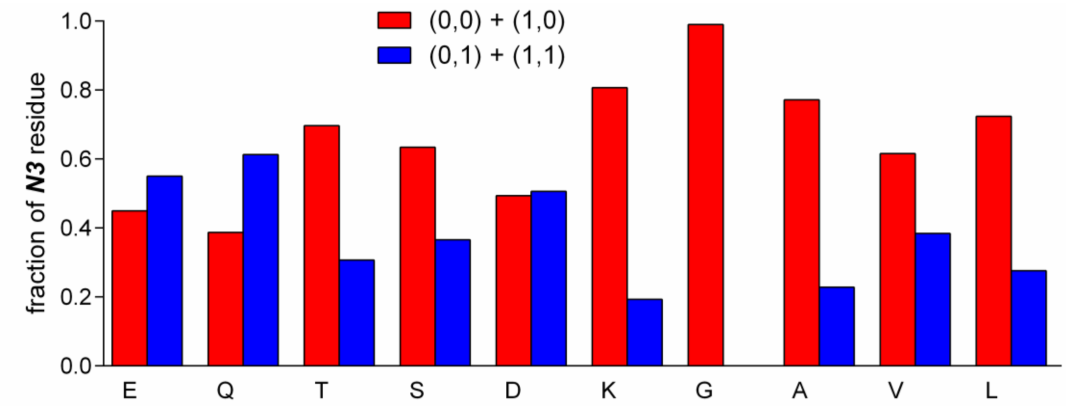

Supplementary Figure 11 Distribution of *N3* residues of helical ASX motifs in two categories: no interactions (0,0) + (1,0), and interactions (0,1 + 1,1). *N3* Residue abundance in different hydrophobic patterns were also analyzed. Here (1,0) and (0,0) were combined as one class because *N3* did not participate in their hydrophobic interactions. (1,1) and (0,1) were also combined because *N3* interacts with *N'* in both classes. Residues that occur in more than 500 motifs were analyzed in this study. E,Q and D are more likely to participate in (*N'*,*N3*) hydrophobic interactions, probably due to the cooperative effect between capping boxes and triangles mentioned in the main text. When these sidechains form capping boxes with *Ncap* amides, the distance between *N'* and *N3* are closer and the methylene groups in these hydrophilic sidechains are in sweet spots to interact with hydrophobic sidechains of *N'* to form hydrophobic interactions. The other residues are less favorable to participate in (*N'*,*N3*) interactions in proteins probably due to the absence of the cooperative effects. Gly has no sidechains hence can not interact with *N'*.

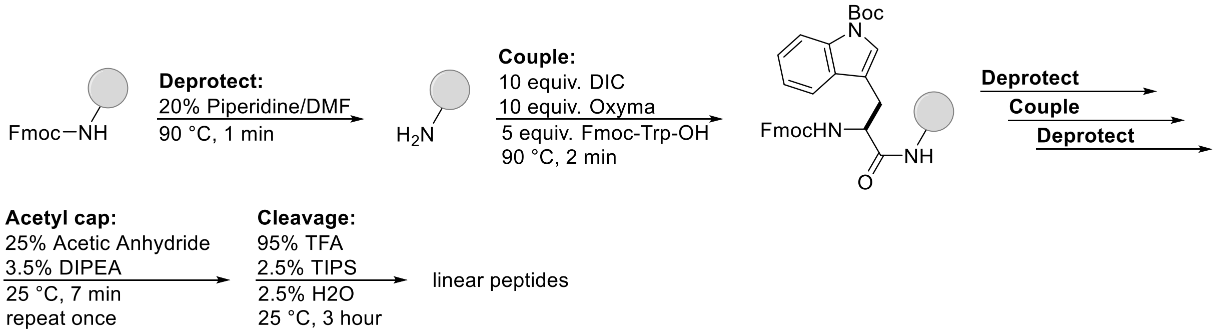

Supplementary Figure 12 Synthesis route of linear peptides.

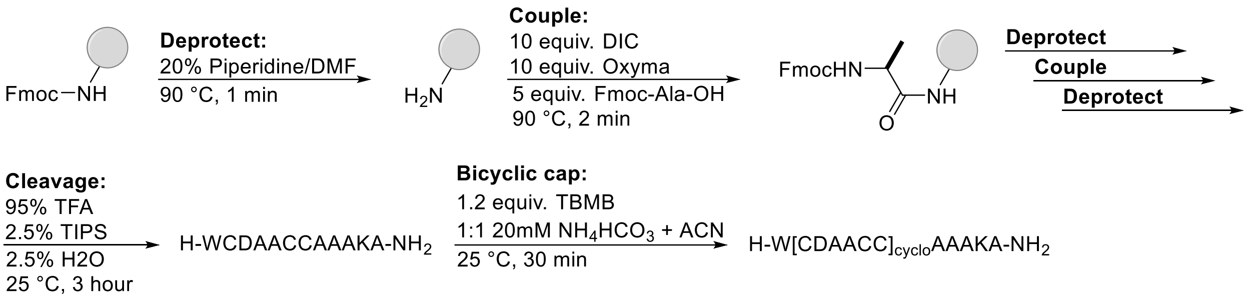

Supplementary Figure 13 Synthesis route of bicyclic peptides.

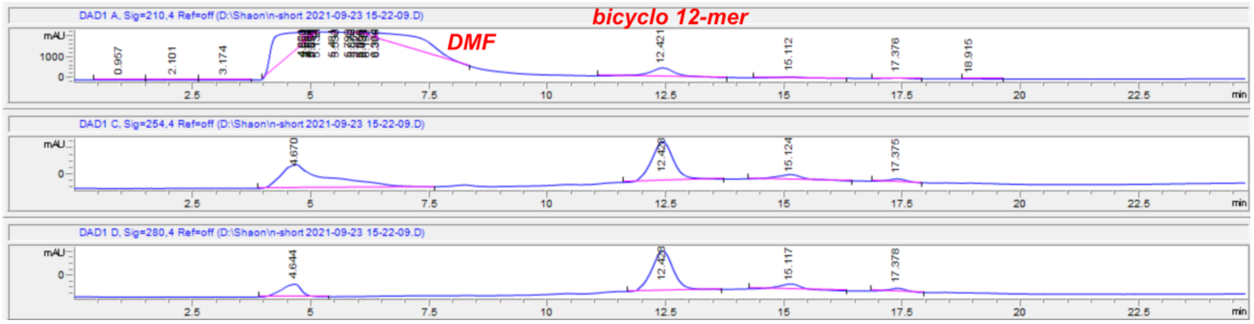

Supplementary Figure 14 Prep HPLC trace of *crude* bicyclo 12-mer. Condition: 20% (0.1% TFA/water)/ 80% (0.1% TFA/acetonitrile) to 80% (0.1% TFA/water)/ 20% (0.1% TFA/acetonitrile) in 25 min. Crude bicyclic peptides were semi-pure, demonstrated by only one major peak in HPLC traces during purification (other than solvent). This proved the high accessibility of the new bicyclic caps. mAU refers to mini Arbitrary Unit.

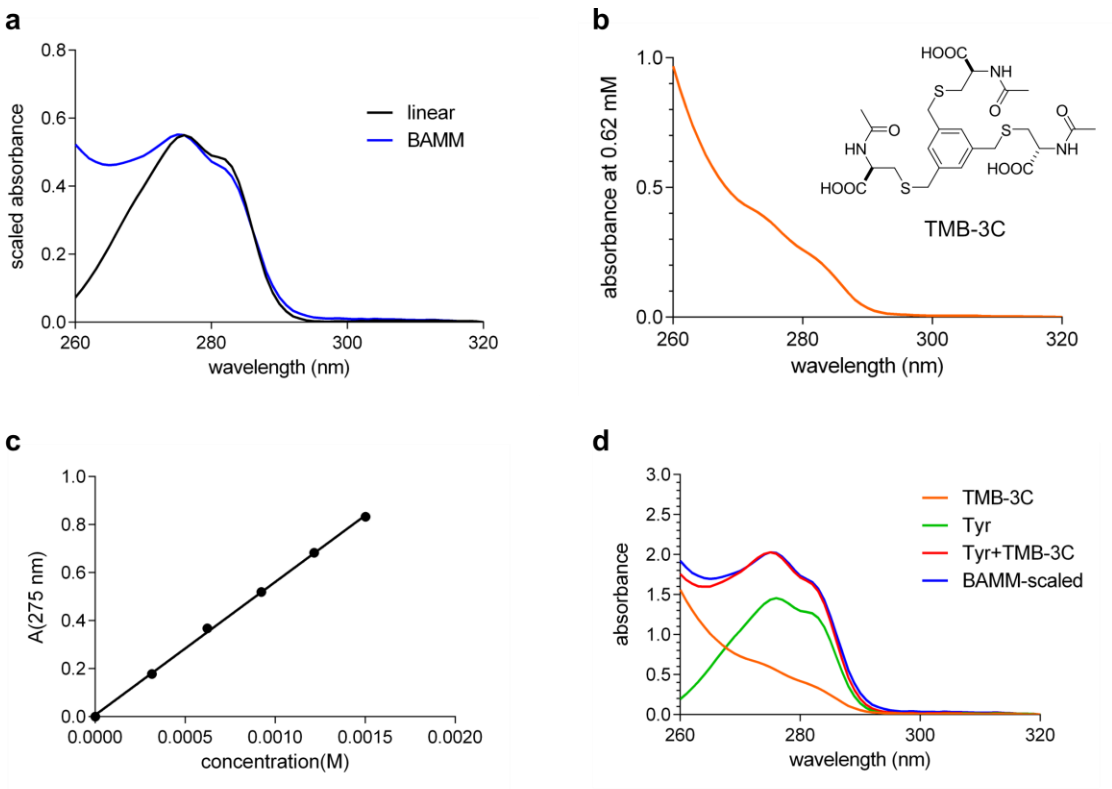

Supplementary Figure 15 Extinction Coefficient of Tri-thiol alkylated TMB **a.**UV spectra of BAMM-capped peptides and linear peptides. **b.** UV spectra of tri-thiol alkylated TMB. **c.** Fittings of A (275 nm) at different concentrations for TMB-3C (n = 1). **d** Merging equal-molar Tyr and TMB-3C gives very close UV spectrum as BAMM *N*-capped peptides.

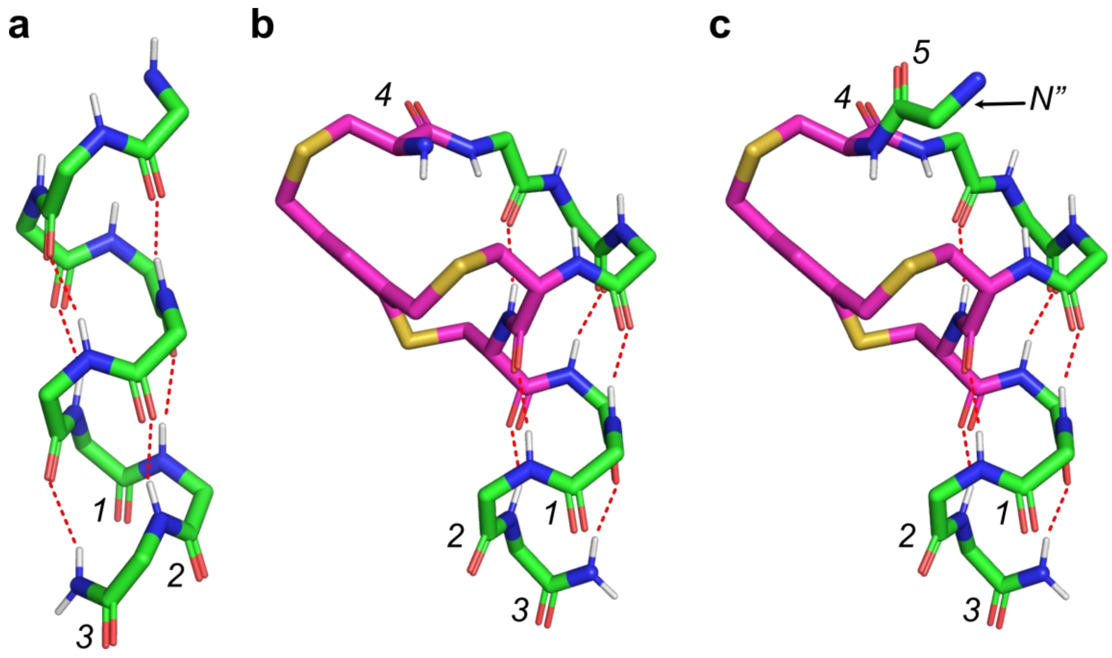

Supplementary Figure 16 Non-H-bonded peptide carbonyls. In ideal helical conformations, **a.** linear peptides have 3 non-*H*-bonded peptide carbonyls, while bicyclic-capped peptides have **b.** 4 or **c.** 5 (magenta represent three Cys and TMB segment).

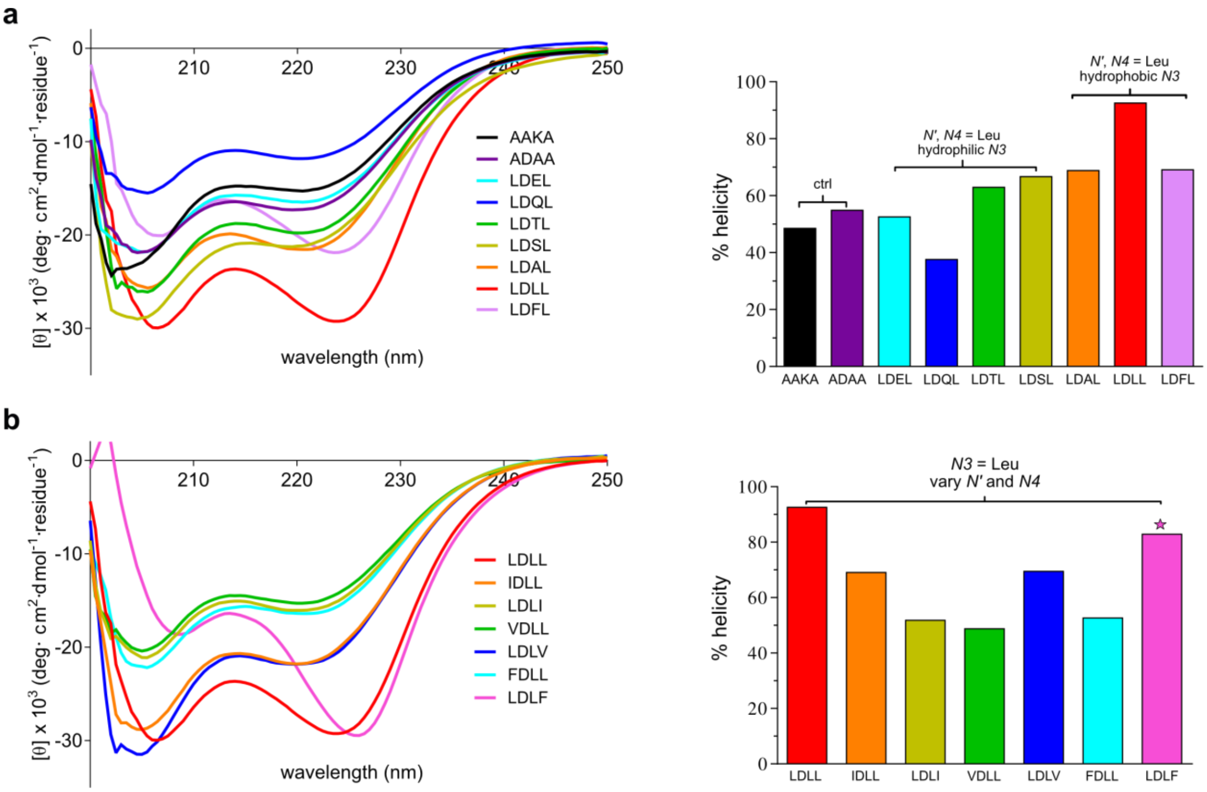

Supplementary Figure 17 CD Spectra of Synthesized Linear Model Peptides in PBS Buffer. Peptides were synthesized, dissolved in PBS buffer (pH 7.4) and tested at 25.0 °C. CD curves were shown on the left-hand side and % helicities on the right-hand side. **a.** smoothed CD curves (left) and corresponding % helicities (right) of peptides including controls and series with various *N3* residues. **b.** smoothed CD curves (left) and corresponding % helicities (right) of peptides with different (*N'*,*N4*) residue pairs. The purple star on LDLF means this peptide may not be a pure α-helix based on its special CD spectrum shape, so its calculated % helicity is only for reference and should not be compared with other sequences.

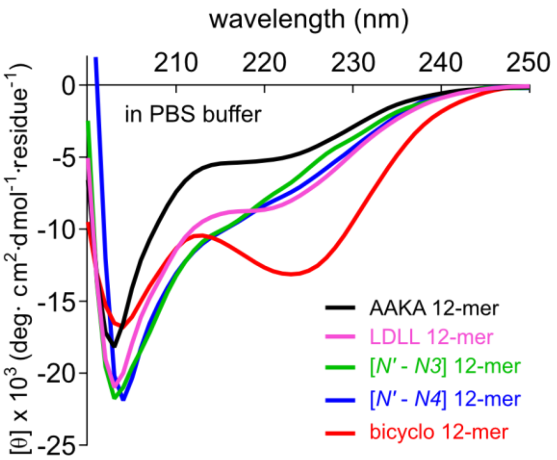

Supplementary Figure 18 Smoothed CD curves of bicyclo 12-mer and four controls in PBS buffer. Peptides were synthesized, dissolved in PBS buffer (pH 7.4, 10 mM) and tested at 25.0 °C.

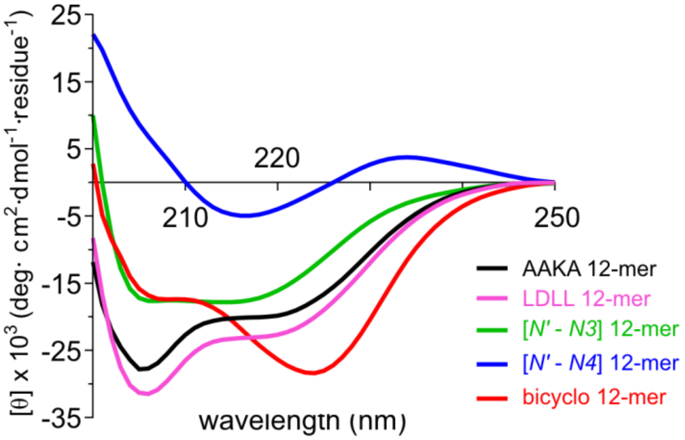

Supplementary Figure 19 Smoothed CD curves of bicyclo 12-mer,four controls in 20% TFE/PBS buffer. Peptides were dissolved in 20% TFE/PBS buffer and tested at 25.0 °C. 12-mers showed interesting behaviors in 20% TFE/PBS. Based on CD spectra, both linear controls were partially helical. Bicyclo 12-mer was highly helical in this medium and showed 'curvature' effect which significantly enhanced ellipticity intensity at 222 nm in Ala-rich helical peptides. The impact is even larger when peptides are *N*-capped.[3] The CD spectra of two monocyclic controls were not like those of α-helices in 20% TFE/PBS. The narrower stapled peptide, [*N'* – *N3*] 12-mer, has a peak around 216 nm, similar to an antiparallel β-sheet.[4] The wider one, [*N'* – *N4*] 12-mer, shows a intriguing shape in CD spectra. We did not find a single secondary structure to show such shape, so it is probably a mixture of clusters with different secondary structures.

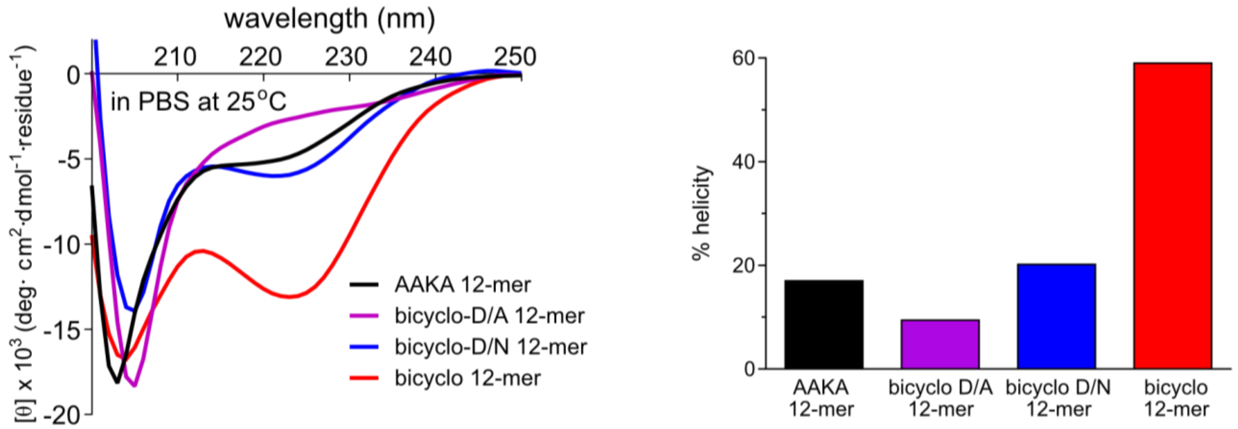

Supplementary Figure 20 Smoothed CD curves of bicyclo 12-mer, AAKA 12-mer,and two controls. To test if the Asp at *Ncap* in bicyclo 12-mer (WC**D**AACCAAAKA) is essential and irreplaceable, another two controls were prepared. The first control replaced Asp with Asn, the other featuring amino acid in ASX motifs, and we called it bicyclo-D/N 12-mer (WC**N**AACCAAAKA). The other control replaced Asp with Ala, *ie* bicyclo-D/A 12-mer (WC**A**AACCAAAKA).

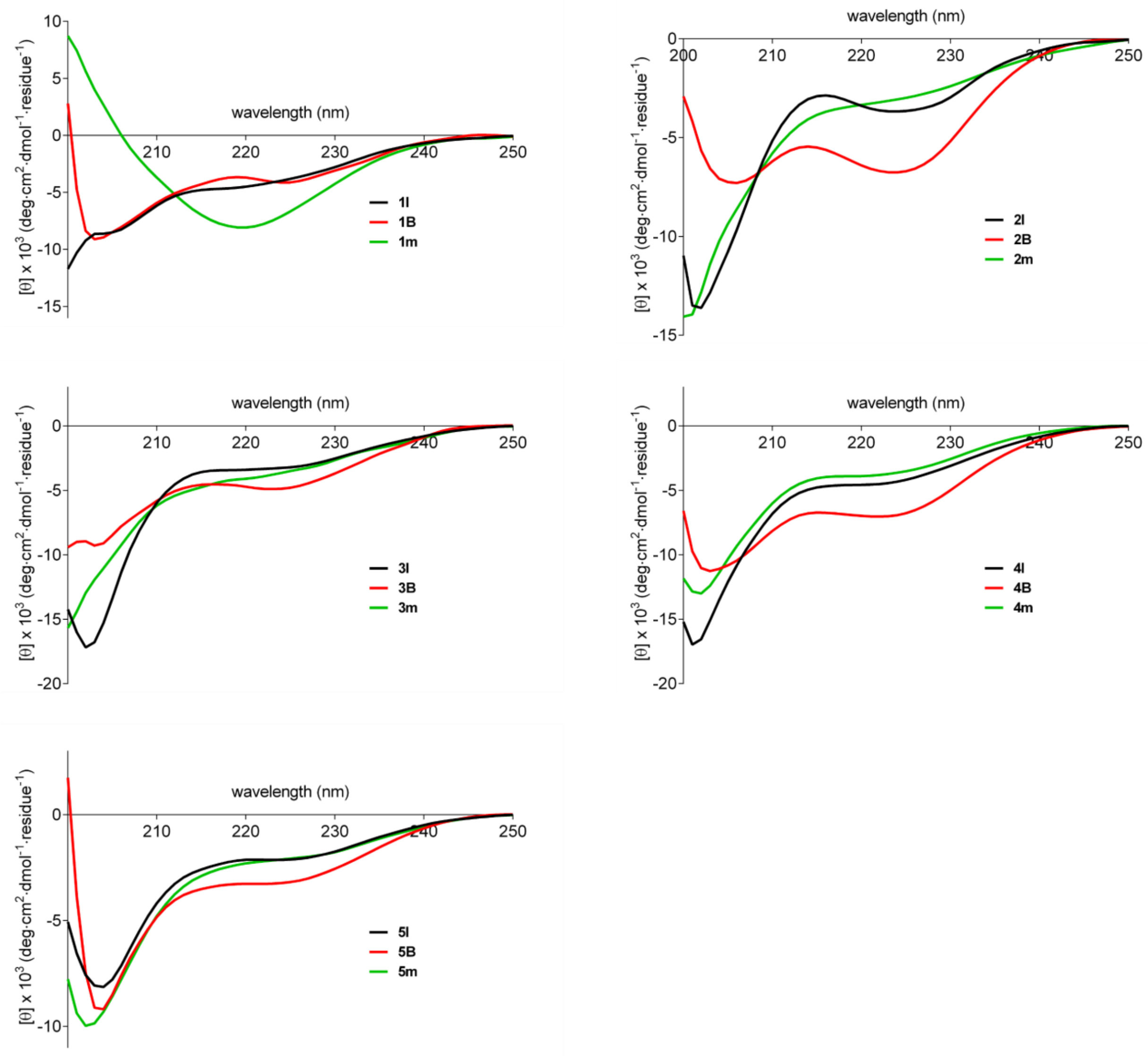

Supplementary Figure 21 CD curves of peptide series 1 - 5 in PBS buffer. Peptides were synthesized, dissolved in PBS buffer (pH 7.4, 10 mM) and tested at 25.0 °C. Although partially helical, increased intensities at $\theta_{[222]}$, and more helical-like CD shapes were observed in BAMM analogues over linear controls. It indicated that BAMM analogues were robustly useful in inducing helicity for these linear peptides in PBS buffer.

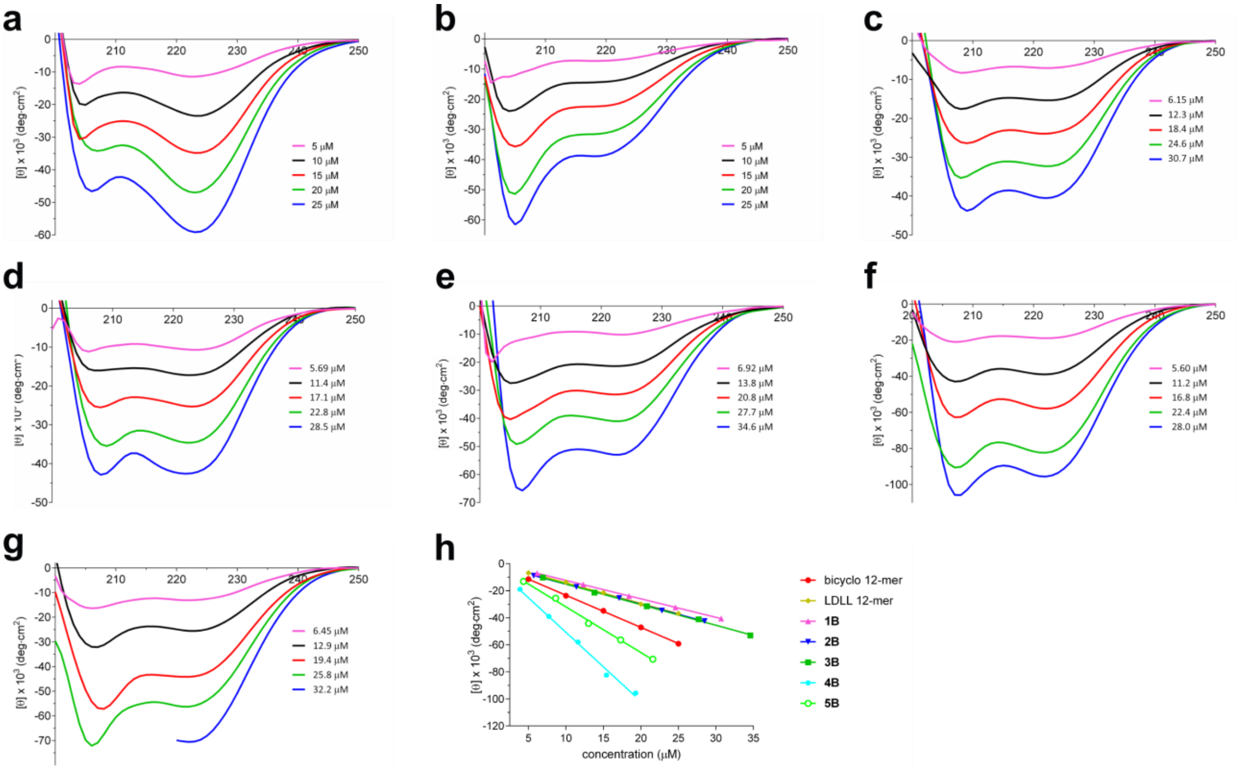

Supplementary Figure 22 Smoothed CD curves of **a.** bicyclo 12-mer, **b.** LDLL 12-mer, **c. 1B**, **d. 2B**, **e. 3B**, **f. 4B** and **g. 5B** (at 21.6 μM its curve becomes scrambled before 220 nm due to machine limitation) in 10% TFE/PBS. **h.** Linear regressions on five concentration points for the tested peptides. To test if BAMM peptides and LDLL 12-mer, the linear peptide with natural hydrophobic triangle, aggregated within concentrations we used in this study, careful oligomeric tests were facilitated. Each peptide was dissolved in 10% TFE/PBS to prepare five samples with concentrations between 3 and 35 μM. These samples were tested in CD experiments, and their $\theta_{[222]}$ were fit by linear regression. There was no obvious change of shapes for the five peptides as concentrations increased, and the linear regression all gave R square values > 0.995. This confirmed there were no significant aggregations in BAMM peptides and LDLL 12-mer within tested concentrations.

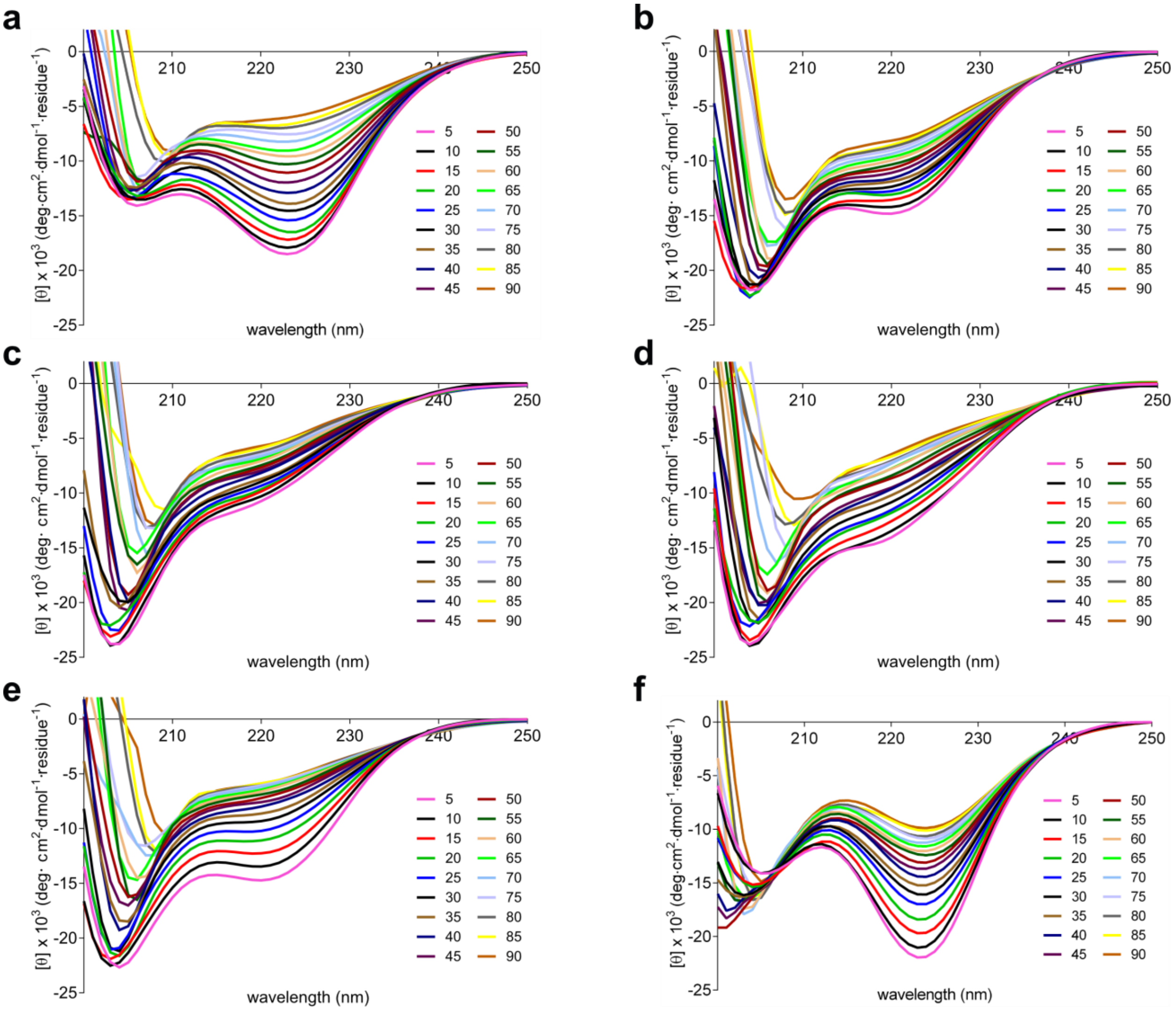

Supplementary Figure 23 Variable CD experiments. Smoothed CD curves in temperatures from 5 to 90 °C for **a.** bicyclo 12-mer, **b.** LDLL 12-mer, **c.** [*N' – N3*] 12-mer, **d.** [*N' – N4*] 12-mer , **e.** AAKA 12-mer in 10% TFE/PBS and **f.** BSM C-capped 12-mer in 10% TFE/PBS.

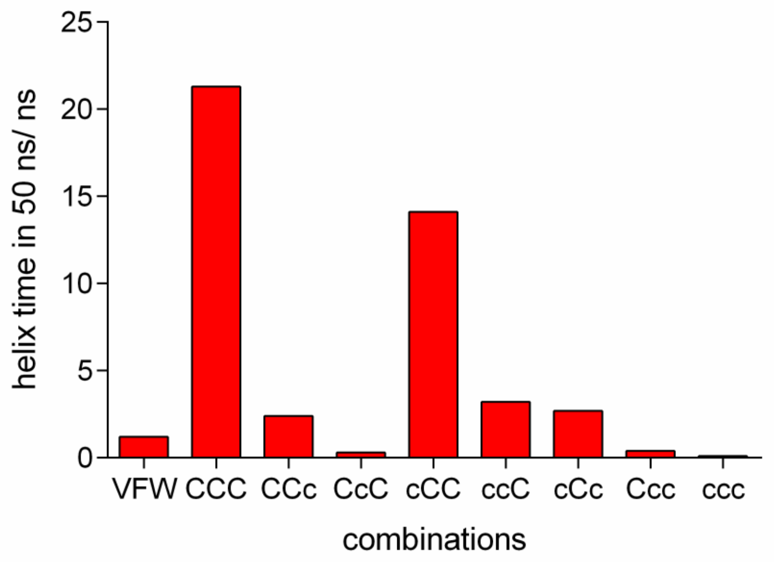

Supplementary Figure 24 Time of different peptides required to unwind from helical into non-helical states. VFW refers to the linear control with a natural hydrophobic triangle; the others are bicyclic peptides with different combinations of L- and D-Cys cyclized with TBMB to mimic the hydrophobic triangle.

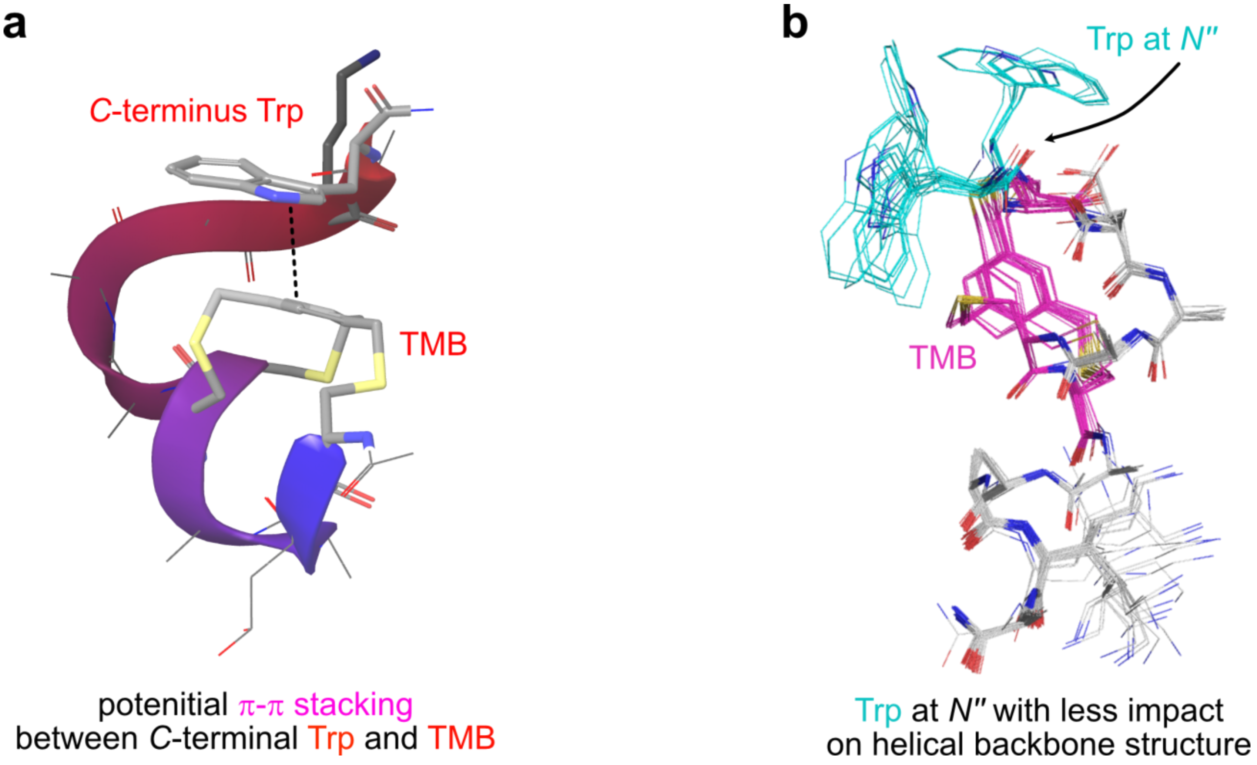

Supplementary Figure 25 Why Putting Trp At N-terminus in Bicyclo 12-mer and Monocyclic Controls. a. A frame from MD simulation indicating potential π - π stacking interactions between TMB and *C*-terminal Trp. **b.** *N*-terminal Trp in low energy conformers of bicyclo 12-mer derived from NMR constraints. Trp was put at the *N*-terminus of bicyclo 12-mer, because in MD simulations we found the *C*-terminal Trp could sometimes fold back towards the *N*-terminal TMB group and potentially formed π-π stacking interactions (Supplementary Figure 25a). Such folding is unfavorable for helical structures, so we put the Trp at the *N*-terminus of bicyclo 12-mer. Besides, the Trp at the *N*-terminal is at the *N''* position, which is outside of a α-helix (helical features start at *N1*), so less impact to the helical backbone is anticipated (Supplementary Figure 25b). Due to the same reason, the Trp in two monocyclic controls were also put at the *N''* position, serving as controls to bicyclo 12-mer.

Ac-L1-D-A1-A3-L2-L3-A4-A5-A2-K-A6-W

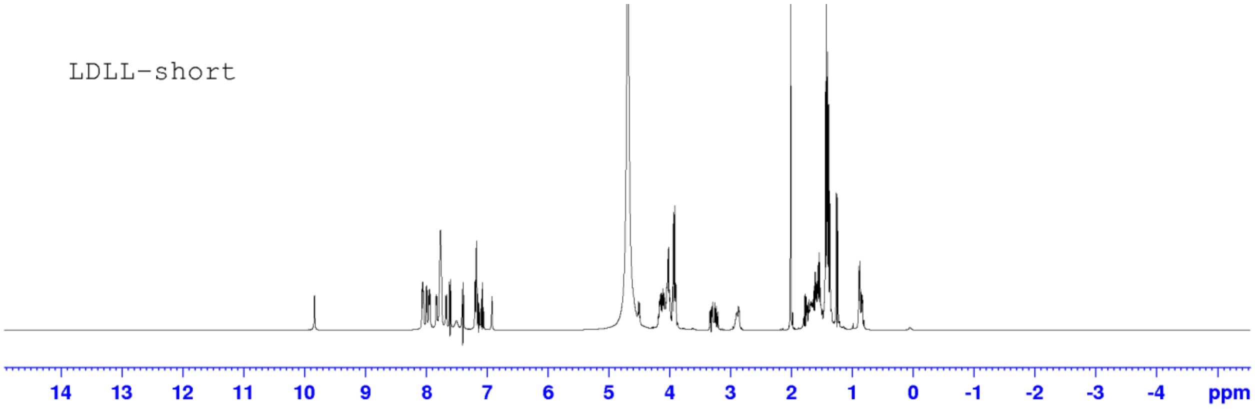

Supplementary Figure 26 1D H[1] NMR spectrum of LDLL 12-mer.

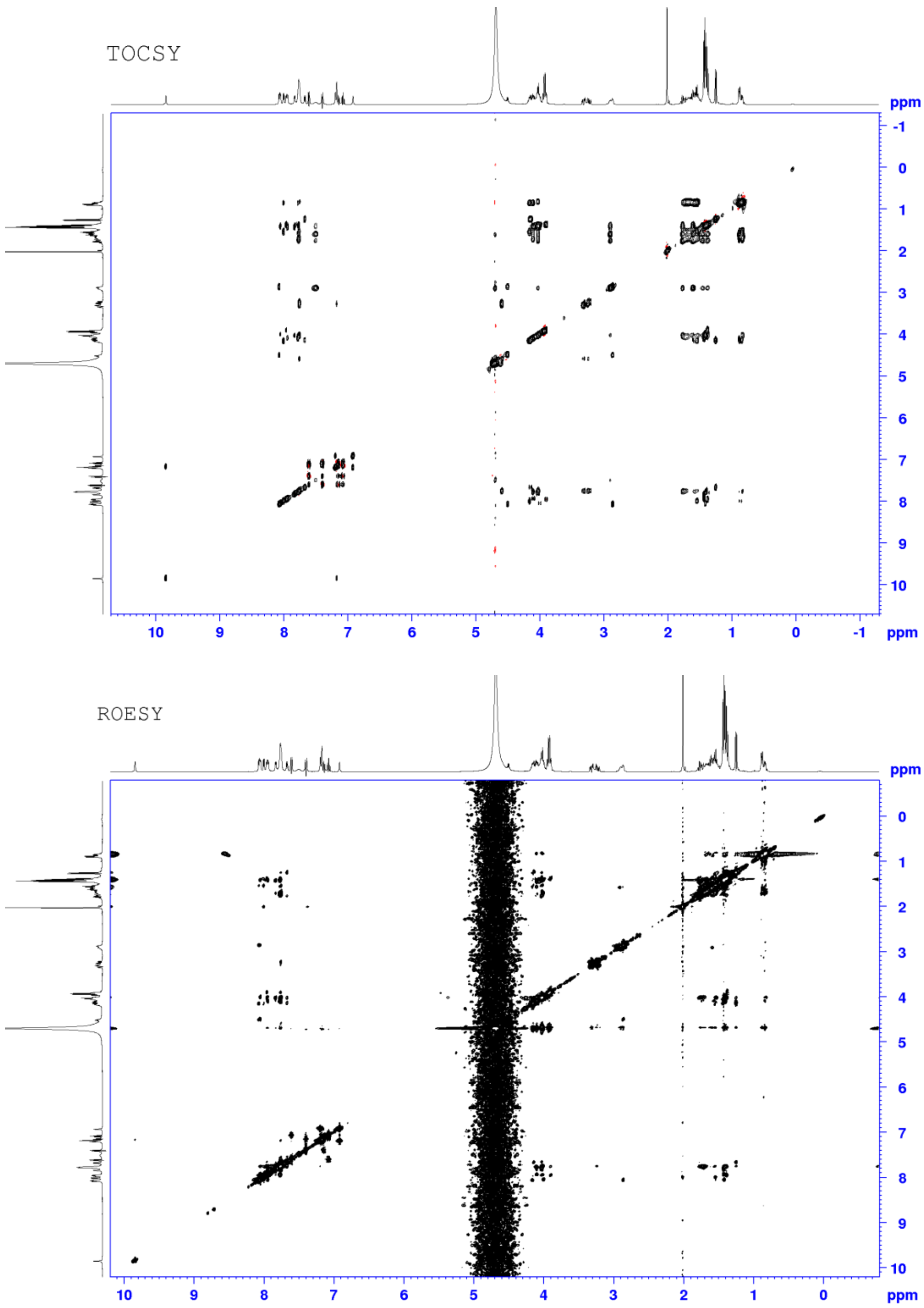

Supplementary Figure 27 2D TOCSY (top) spectrum and ROESY (bottom) spectrum of LDLL 12-mer

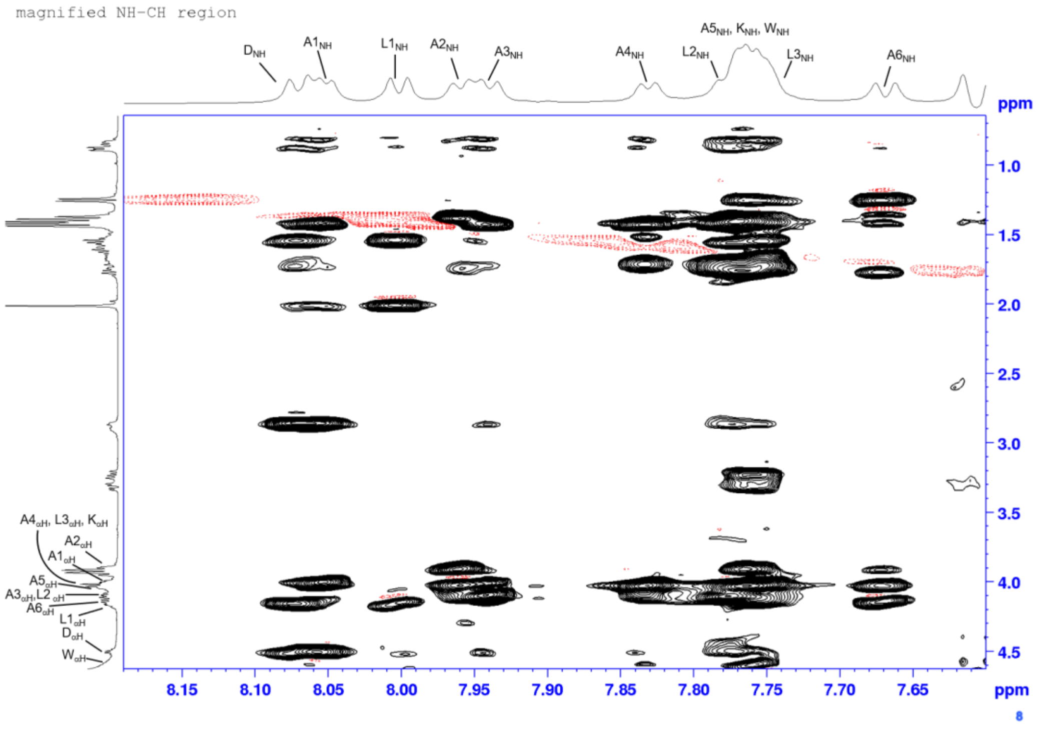

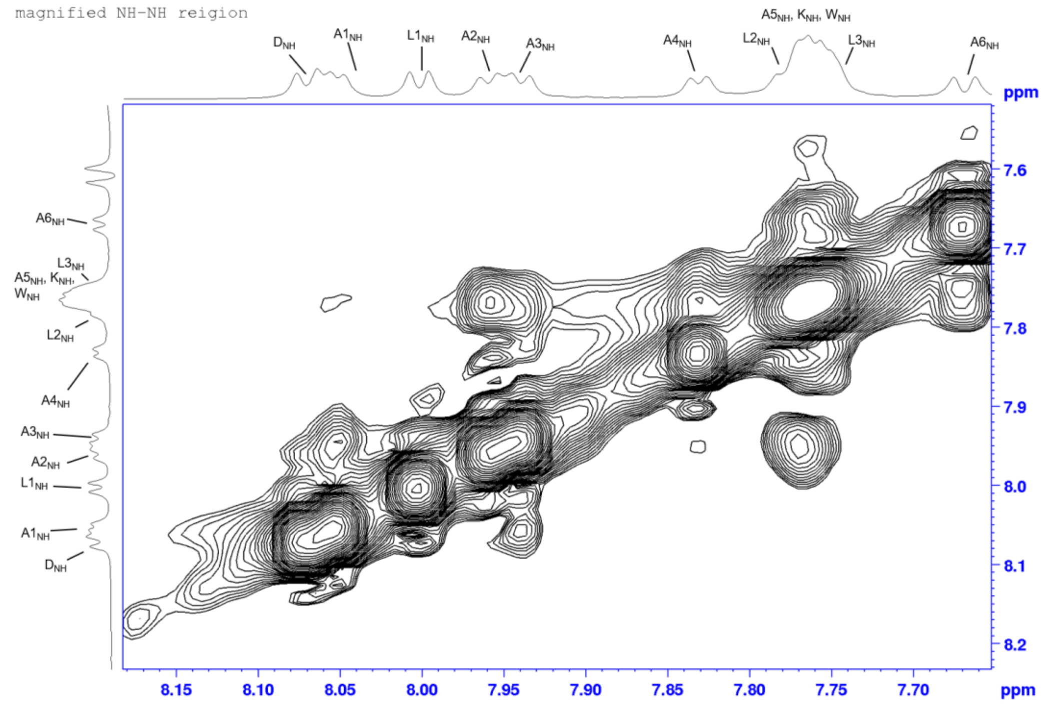

Supplementary Figure 28 magnified fingerprints regions of LDLL 12-mer from the ROESY spectrum.

Ac-L1-D-A1-A3-L2-L3-A4-A5-A2-K-A6-W-NH_2

Supplementary Table 1 Peak assignments for all *H* in LDLL 12-mer and coupling constants between *N<u>H</u>* and α*<u>H</u>*

Residue	αH	βH	γH	δH	εH	εNH	NH	Aromatic	$^3J_{NH\text{-}\alpha H}$ (Hz)
Ac-	2.01								
L1	4.16	1.55	1.55	0.86			8.00		5.8
D	4.51	2.86					8.07		6.2
A1	4.00	1.42					8.05		4.2
A3	4.10	1.43					7.94		5.4
L2	4.10	1.73	1.58	0.88			7.78		
L3	4.03	1.69	1.53	0.83			7.75		
A4	4.03	1.43					7.83		4.8
A5	4.05	1.4					7.77		
A2	3.90	1.38					7.96		5.4
K	4.03	1.77	1.37, 1.45	1.6	2.9	7.5	7.76		
A6	4.14	1.25					7.67		6.6
W	4.59	3.22, 3.32					7.76	7.07, 7.15, 7.17, 7.40, 7.61	7.2
-NH_2							6.92, 7.20		

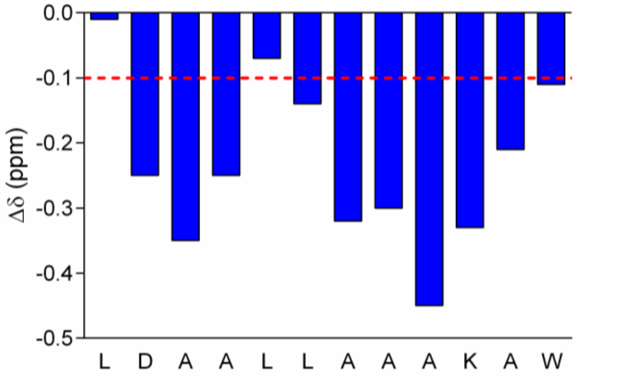

Supplementary Figure 29 CSI graph of LDLL 12-mer.

Supplementary Table 2 Distance constraints from ROESY spectrum.

Atom1	Chemical shift	Atom2	Chemical shift		Upper limit	Lower limit
**Within Residue**						
**L1**						
L1γ	1.55	L1α	4.17		3.75	2.50
L1α	4.17	L1β	1.57		4.07	2.71
L1NH	8.00	L1α	4.17		3.88	2.58
L1NH	8.00	L1β	1.54		3.38	2.25
**D**						
Dβ	2.86	Dα	4.49		3.33	2.22
DNH	8.08	Dα	4.50		3.70	2.46
DNH	8.07	Dβ	2.86		3.13	2.09
**A1**						
A1NH	8.05	A1α	4.00		3.30	2.20
A1NH	8.06	A1β	1.42		3.13	2.09
**A3**						
A3β	1.43	A3α	4.10		2.84	1.89
A3NH	7.94	A3α	4.09		3.51	2.34
A3NH	7.93	A3β	1.43		2.97	1.98
**L2**						
L2δ	0.89	L2β2	1.71		3.67	2.45
L2β1	1.74	L2α	4.10		3.84	2.56
L2γ	1.57	L2α	4.08		3.51	2.34
L2α	4.09	L2β2	1.71		4.15	2.77
**L3**						
L3β1	1.71	L3γ	1.53		2.68	1.79
L3δ2	0.82	L3β2	1.66		4.24	2.83
L3δ1	0.81	L3α	4.02		4.52	3.01
L3δ2	0.82	L3α	4.02		3.92	2.62
L3γ	1.53	L3α	4.02		3.35	2.24
L3NH	7.75	L3δ2	0.82		4.57	3.05
**A4**						
A4NH	7.83	A4α	4.02		3.16	2.11
A4NH	7.83	A4β	1.42		2.98	1.99
**A5**						
**A2**						
A2α	3.90	A2β	1.38		2.84	1.89
A2NH	7.96	A2α	3.89		3.43	2.28
A2NH	7.96	A2β	1.36		3.15	2.10
**K**						
Kβ1	1.76	Kγ1	1.38		3.22	2.15
Kβ2	1.79	Kγ2	1.45		3.62	2.41

Kβ1	1.77	Kα	4.02		3.16	2.10
**A6**						
A6α	4.15	A6β	1.24		3.50	2.33
A6NH	7.67	A6α	4.15		3.49	2.33
A6NH	7.67	A6β	1.25		3.20	2.13
**W**						
aro-e	7.40	aro-d	7.15		3.08	2.06
aro-b	7.61	aro-c	7.06		3.93	2.62
aro-a	7.17	Wβ2	3.31		4.89	3.26
aro-a	7.17	Wβ1	3.22		5.12	3.41
aro-b	7.60	Wα	4.58		5.00	3.34
aro-b	7.61	Wβ2	3.29		4.86	3.24
WNH	7.76	Wα	4.59		3.76	2.51
WNH	7.76	Wβ2	3.32		3.81	2.54
WNH	7.76	Wβ1	3.23		3.48	2.32
aro-NH	9.84	aro-a	7.18		4.16	2.77
**C-terminus amide**						
C-N2	7.20	C-N1	6.92		2.04	1.36
**Cross residues**						
**N(i)-N(i+1)**						
WNH	7.76	A6NH	7.69		3.04	2.02
KNH	7.76	A6NH	7.65		3.29	2.19
A5NH	7.77	A4NH	7.86		2.92	1.95
WNH	7.76	C-N2	7.19		3.91	2.61
A2NH	7.96	A6NH	7.66		4.80	3.20
A4NH	7.83	L3NH	7.77		2.86	1.90
A3NH	7.94	L2NH	7.78		3.03	2.02
A2NH	7.97	A5NH	7.79		3.02	2.01
A2NH	7.96	KNH	7.74		3.45	2.30
A1NH	8.06	A3NH	7.94		2.96	1.97
**N(i)-N(i+2)**						
A2NH	7.96	A4NH	7.84		4.14	2.76
**α(i)-N(i+1)**						
A6NH	7.67	Kα	4.02		3.55	2.37
A3NH	7.93	A1α	4.00		4.43	2.95
DNH	8.07	L1α	4.16		3.38	2.25
A1NH	8.05	Dα	4.50		3.44	2.29
L1NH	8.00	Aca	2.01		3.37	2.25
**α(i)-N(i+2)**						
A6NH	7.67	A2α	3.91		4.83	3.22
A3NH	7.94	Dα	4.50		5.47	3.65
**α(i)-N(i+3)**						
WNH	7.76	A2α	3.90		3.55	2.36
A4NH	7.83	A3α	4.10		3.61	2.41

A2NH	7.96	L3α	4.01		3.62	2.41
**β(i)-N(i+1)**						
A1NH	8.04	Dβ	2.84		3.94	2.63
A4NH	7.84	L3β1	1.71		3.68	2.46
DNH	8.07	L1β	1.55		3.67	2.45
A6NH	7.67	Kβ1	1.77		3.98	2.66
WNH	7.76	A6β	1.25		3.76	2.50
**α(i)-β(i+3)**						
L3β1	1.70	A1α	4.01		3.64	2.43
A6β	1.25	A5A	4.03		3.74	2.49
A2α	3.90	Wβ1	3.21		4.28	2.85
**Cap-part**						
L2δ2	0.89	L3δ1	0.81		3.34	2.23
L1δ1	0.85	L3β2	1.70		3.42	2.28
L1δ1	0.85	L3α	4.02		4.26	2.84
L3δ2	0.82	L2α	4.10		4.25	2.84
Dβ	2.86	L2β2	1.72		4.39	2.93
Dβ	2.87	L2γ	1.57		4.11	2.74
Dβ	2.86	L1δ1	0.84		4.75	3.17
L3α	4.04	L2δ2	0.90		5.14	3.43
A3NH	7.94	Dβ	2.85		5.17	3.44
L3NH	7.75	Dβ	2.85		5.00	3.34
L2NH	7.78	Dβ	2.85		4.52	3.01
A3NH	7.94	L2β2	1.72		5.58	3.72
A1NH	8.05	L2β2	1.72		5.50	3.67
L2NH	7.78	L3δ2	0.82		4.56	3.04
A4NH	7.83	L3δ1	0.81		5.62	3.74
A3NH	7.95	L3δ1	0.81		5.70	3.80
A1NH	8.06	L3δ1	0.82		5.44	3.62
**K-sidechain**						
A6NH	7.67	Kγ2	1.41		4.80	3.20
A6NH	7.67	Kγ1	1.35		4.94	3.29

Supplementary Table 3 Coupling constants and corresponding Φ calculated from equation[5] $\{J(\theta) = 6.98 * (\cos(\theta))^2 - (1.38*\cos(\theta)) + 1.72$, where $\theta = |\Phi-60|\}$.

Residue	$^3J_{NH\text{-}\alpha H}$ (Hz)	Φ (°)
L1	5.8	-72 ± 30
D	6.2	-75 ± 30
A1	4.2	-60 ± 30
A3	5.4	-70 ± 30
L2		
L3		
A4	4.8	-65 ± 30
A5		
A2	5.4	-69 ± 30
K		
A6	6.6	-78 ± 30
W	7.2	*inconclusive*

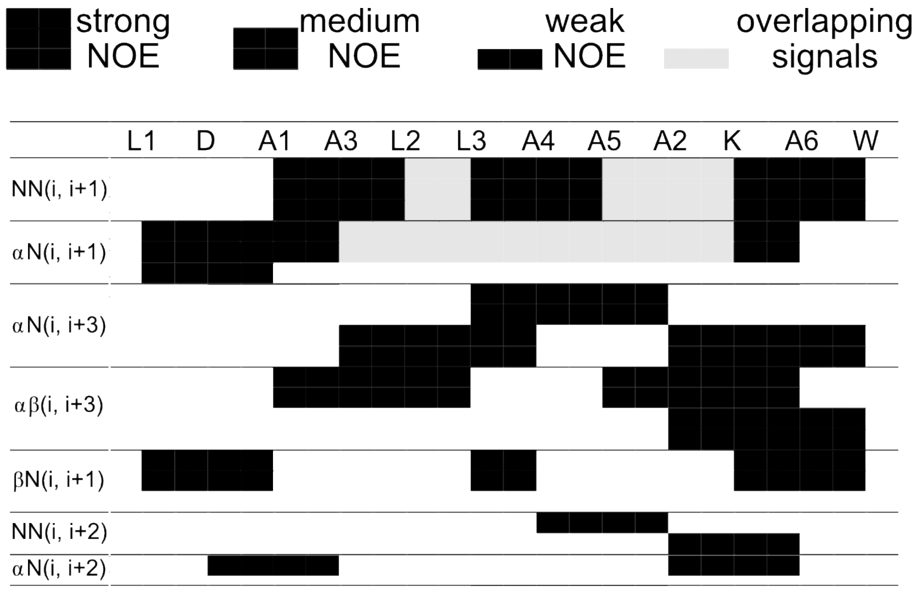

Supplementary Figure 30 Characteristic cross-residue ROE in LDLL 12-mer.

Ac-L1-D-A1-A3-L2-L3-A4-A5-A2-K-A6-W-NH_2

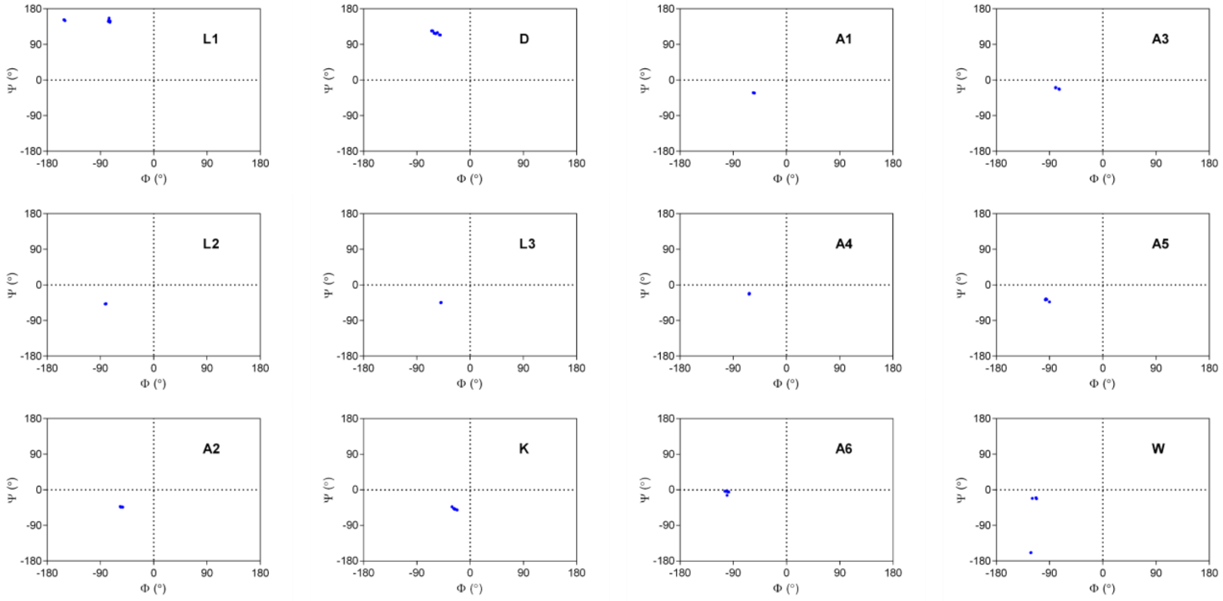

Supplementary Figure 31 Dihedral angles (Φ,ψ) of residues in low energy conformers of LDLL 12-mer.

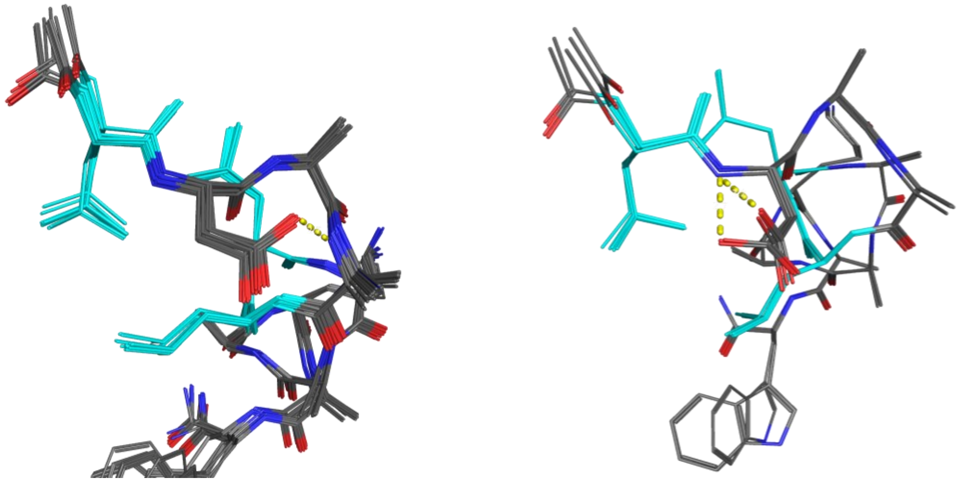

Supplementary Figure 32 Cluster 1 (left) and 2 (right) are different from χ angle in *Ncap* Asp sidechain. Cluster 1 (major cluster, 18/23): ASX turn; Cluster 2 (minor cluster, 5/23): Sidechain of Asp has hydrogen bond to its amide.

W-[C2-D-A1-A4-C3-C1]$_{cyclo}$-A2-A5-A3-K-A6

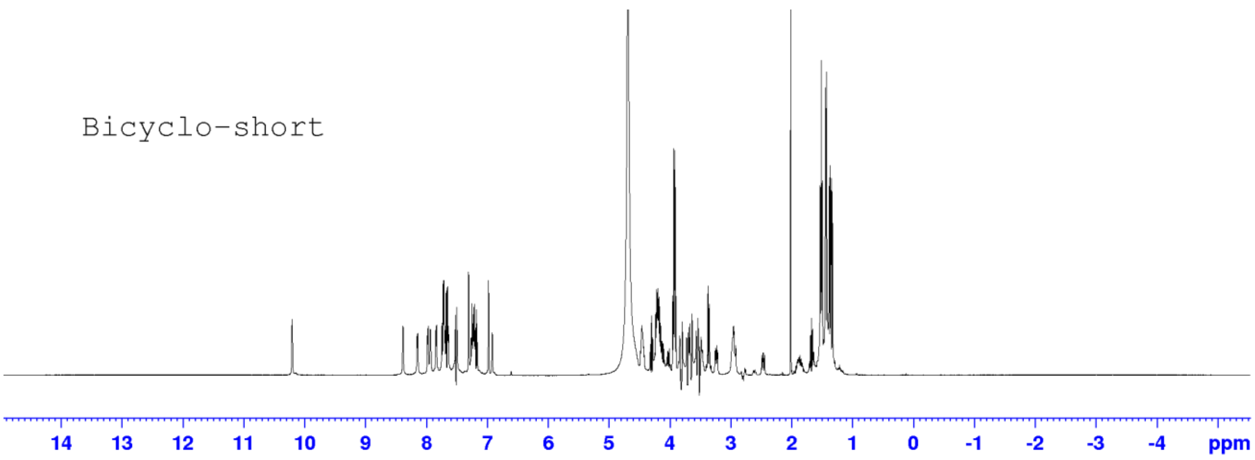

Supplementary Figure 33 1D H[1] NMR spectrum of bicyclo 12-mer.

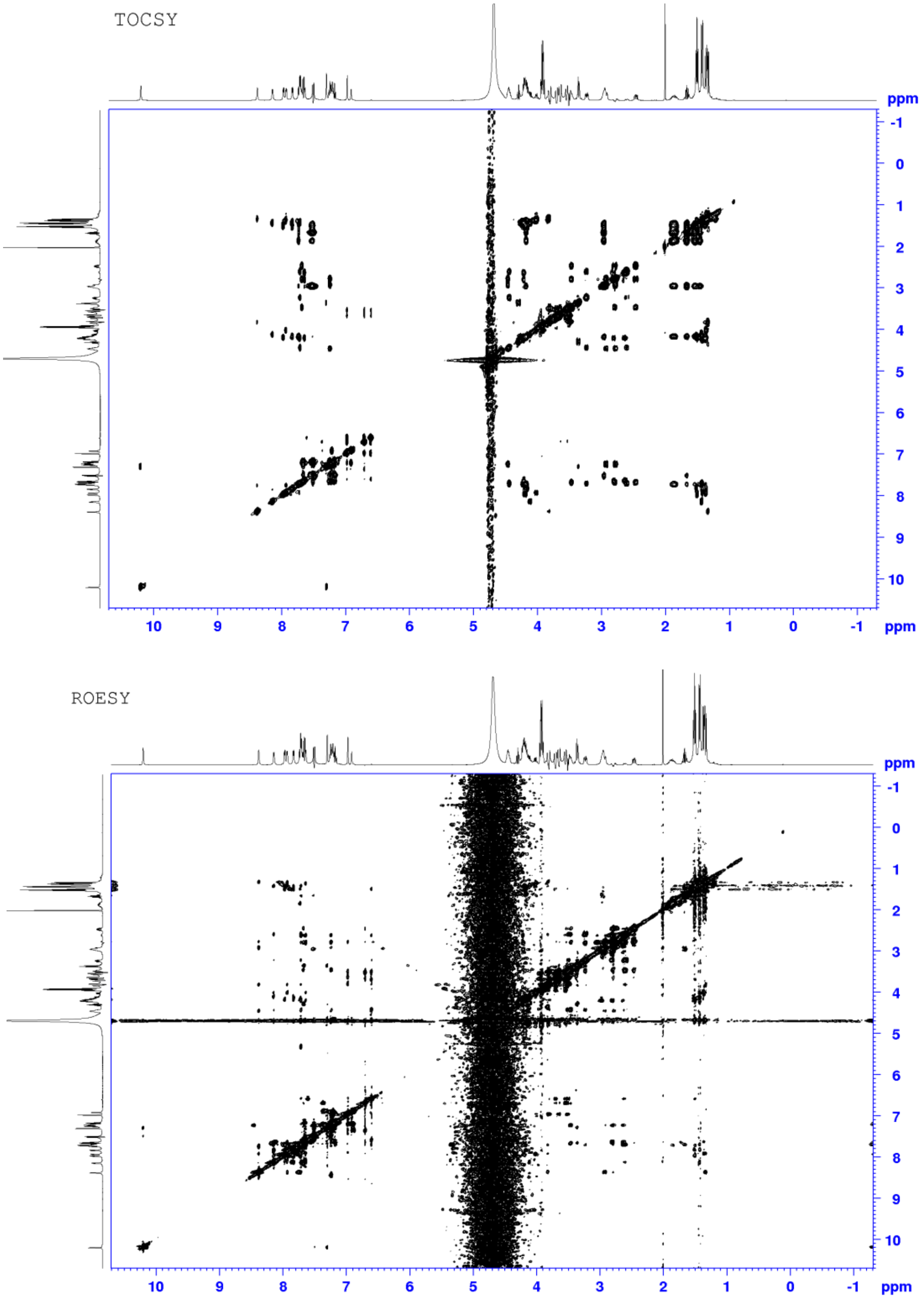

Supplementary Figure 34 2D TOCSY (top) spectrum and ROESY (bottom) spectrum of bicyclo 12-mer

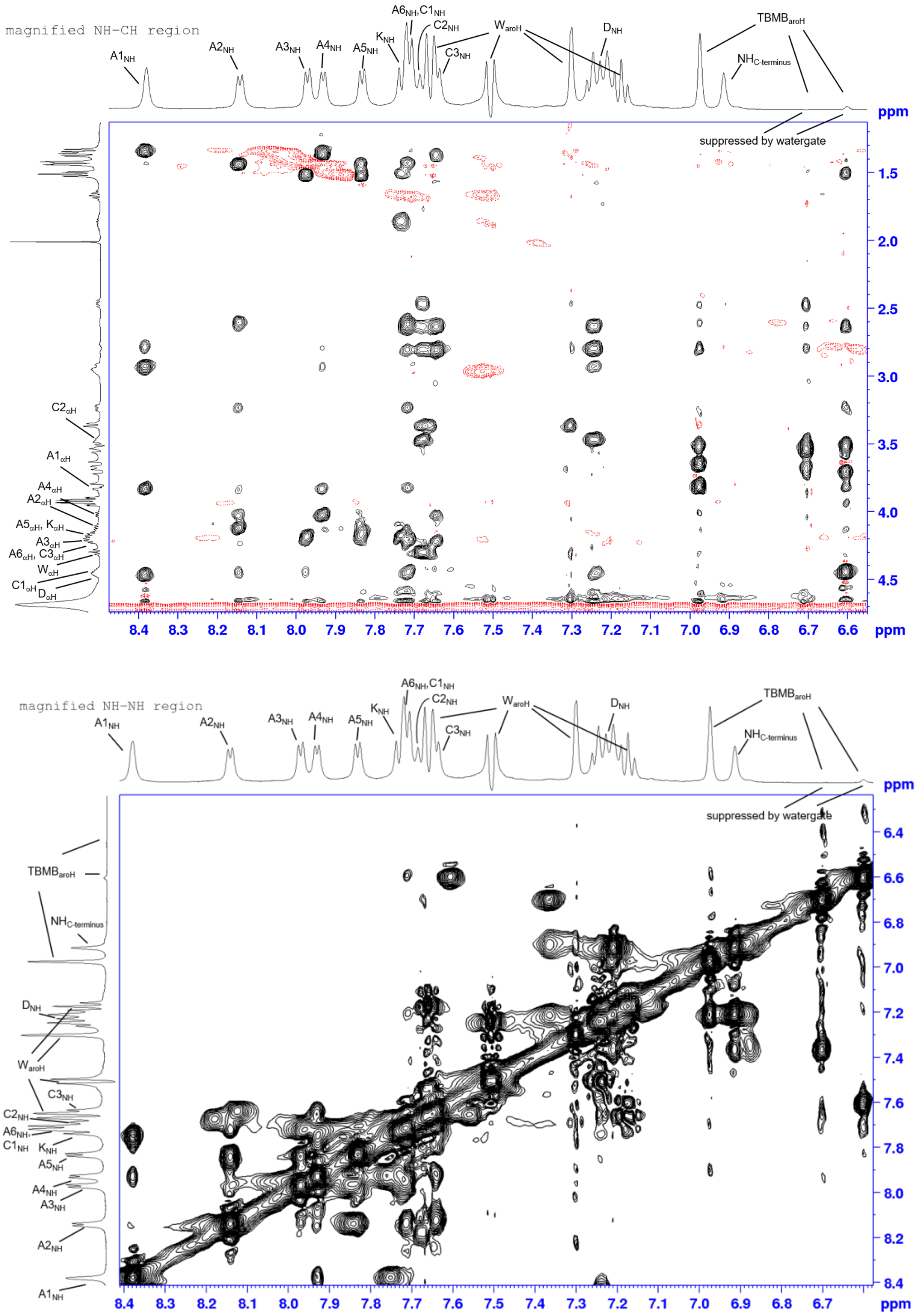

Supplementary Figure 35 magnified fingerprints regions of bicyclo 12-mer from the ROESY spectrum.

W-[C2-D-A1-A4-C3-C1]$_{cyclo}$-A2-A5-A3-K-A6-NH_2

Supplementary Table 4 Peak assignments for all *H* in bicyclo 12-mer and coupling constants between *N<u>H</u>* and α*<u>H</u>*

Residue	αH	βH	γH	δH	εH	εNH	NH	Aromatic	$^3J_{NH\text{-}\alpha H}$ (Hz)
W	4.29	3.36						7.17, 7.25, 7.30, 7.51, 7.66	
C2	3.47	2.47, 2.79					7.68		6.8
D	4.46	2.78, 2.93					7.24		8.8
A1	3.82	1.33					8.38		
A4	4.02	1.37					7.93		4.8
C3	4.21	2.62, 2.80					7.64		5.2
C1	4.44	2.60, 3.23					7.71		5.2
A2	4.11	1.43					8.14		5.2
A5	4.16	1.52					7.83		5.5
A3	4.19	1.5					7.97		5.3
K	4.17	1.87	1.46, 1.54	1.66	2.96	7.52	7.73		5.2
A6	4.23	1.43					7.71		
NH_2							6.92, 7.21		
TMB	αH: 3.52, 3.64; 3.55, 3.70; 3.51, 3.81							aromatic: 6.60, 6.70, 6.97	

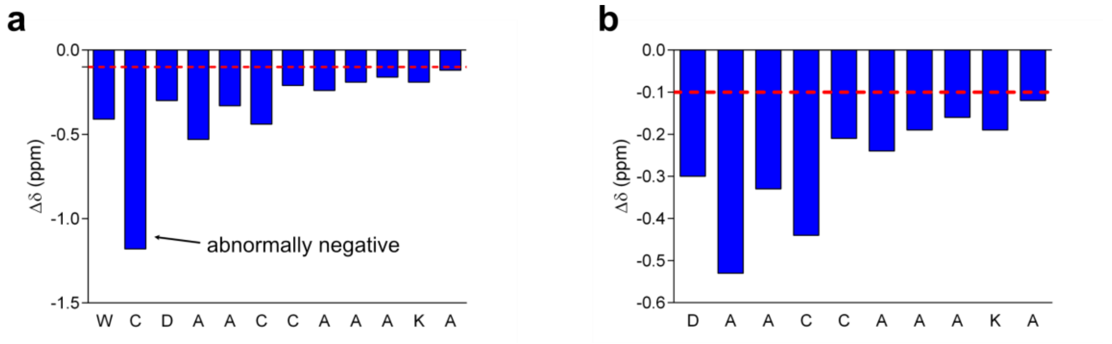

Supplementary Figure 36 a. complete CSI graph of bicyclo 12-mer. **b.** highlighted CSI graph starting at Asp *Ncap*. The abnormally negative CSI of Cys at *N'* is due to shielding effect by TMB group. Corresponding analysis is in Supplementary Figure 40. CSI after that were more negative than -0.1 ppm, which indicates a potential α-helical structure. $\Delta\delta = \delta_{\alpha H}$ (experimental) - $\delta_{\alpha H}$ (random).

Supplementary Table 5 Distance constraints from ROESY spectrum of bicyclo 12-mer

Atom1	Chemical shift	Atom2	Chemical shift		Upper limit	Lower limit
**Within Residue**						
**W**						
Wβ	3.36	Wα	4.28		3.22	2.15
WN	10.20	W-aro-c	7.50		4.55	3.03
WN	10.20	W-aro-a	7.30		3.58	2.38
W-aro-a	7.30	Wα	4.30		5.04	3.36
W-aro-a	7.31	Wβ	3.35		3.58	2.38
**C2**						
C2β2	2.79	C2β1	2.46		2.20	1.47
C2β1	2.46	C2α	3.46		3.50	2.34
C2β2	2.80	C2α	3.45		3.09	2.06
C2N	7.67	C2α	3.47		3.30	2.20
C2N	7.68	C2β2	2.80		3.54	2.36
C2N	7.68	C2β1	2.46		3.73	2.49
**D**						
Dβ2	2.93	Dβ1	2.77		2.08	1.39
Dβ1	2.78	Dα	4.45		3.68	2.45
Dβ2	2.93	Dα	4.46		3.91	2.61
DN	7.23	Dα	4.44		3.80	2.53
DN	7.24	Dβ2	2.92		3.95	2.63
DN	7.24	Dβ1	2.78		3.23	2.15
**A1**						
A1α	3.82	A1β	1.33		2.95	1.97
A1N	8.38	A1α	3.82		3.45	2.30
A1N	8.38	A1β	1.32		3.13	2.09
**A4**						
A4α	4.02	A4β	1.32		3.01	2.01
A4N	7.94	A4α	4.01		3.37	2.25
**C3**						
C3β2	2.81	C3β1	2.61		2.23	1.48
C3β2	2.80	C3α	4.21		3.03	2.02
C3β1	2.64	C3α	4.21		3.70	2.47
C3N	7.64	C3α	4.20		3.80	2.53
C3N	7.64	C3β2	2.80		2.98	1.99
C3N	7.64	C3β1	2.62		3.26	2.17
**C1**						
C1β2	3.24	C1β1	2.60		2.27	1.51
C1β1	2.60	C1α	4.43		3.82	2.55
C1β2	3.23	C1α	4.43		3.52	2.35
C1N	7.72	C1β1	2.57		3.23	2.16
C1N	7.72	C1β2	3.23		4.15	2.76

C1N	7.72	C1α	4.44		3.58	2.39
**A2**						
A2α	4.11	A2β	1.42		2.85	1.90
A2N	8.14	A2α	4.11		3.39	2.26
A2N	8.15	A2β	1.43		3.19	2.13
**A5**						
A5α	4.16	A5β	1.51		2.86	1.91
A5N	7.83	A5α	4.18		3.23	2.16
A5N	7.83	A5β	1.52		3.35	2.23
**A3**						
A3α	4.19	A3β	1.51		3.01	2.01
A3N	7.97	A3α	4.18		3.34	2.23
A3N	7.97	A3β	1.51		3.33	2.22
**K**						
KN	7.74	Kα	4.16		3.30	2.20
KN	7.73	Kβ	1.86		3.36	2.24
**A6**						
A6α	4.21	A6β	1.41		3.23	2.15
A6N	7.72	A6β	1.42		3.78	2.52
**Cross residues**						
**N(i)-N(i+1)**						
A4N	7.93	A1N	8.38		3.60	2.40
C3N	7.64	A4N	7.92		3.28	2.19
A2N	8.15	C1N	7.71		3.03	2.02
A5N	7.85	A2N	8.15		2.68	1.78
A3N	7.98	A5N	7.83		2.84	1.89
A3N	7.98	KN	7.72		2.64	1.76
A6N	7.71	C-N2	7.21		4.16	2.77
**N(i)-N(i+2)**						
A3N	7.97	A2N	8.14		4.28	2.86
**α(i)-N(i+1)**						
C2N	7.68	Wα	4.29		2.96	1.97
DN	7.24	C2α	3.45		3.12	2.08
A1N	8.38	Dα	4.45		3.35	2.24
A4N	7.93	A1α	3.82		4.09	2.73
C3N	7.64	A4α	4.01		4.11	2.74
A2N	8.14	C1α	4.44		4.39	2.93
**α(i)-N(i+3)**						
C1N	7.71	A1α	3.81		4.18	2.78
A2N	8.14	A4α	4.01		4.05	2.70
A3N	7.98	C1α	4.44		4.64	3.09
**α(i)-N(i+2)**						
A2N	8.14	C3α	4.24		5.42	3.61
**α(i)-N(i+4)**						

A2N	8.14	A1α	3.82		5.17	3.44
A5N	7.84	A4α	4.01		5.19	3.46
A6N	7.71	A2α	4.11		5.14	3.43
**β(i)-N(i+1)**						
C2N	7.67	Wβ	3.36		3.36	2.24
A1N	8.38	Dβ2	2.94		3.15	2.10
A1N	8.38	Dβ1	2.77		4.00	2.67
A4N	7.93	A1β	1.34		3.13	2.09
C3N	7.64	A4β	1.35		3.82	2.55
C1N	7.72	C3β1	2.67		3.31	2.21
C1N	7.72	C3β2	2.80		3.97	2.64
A2N	8.14	C1β2	3.22		4.58	3.06
A2N	8.14	C1β1	2.61		3.61	2.41
A5N	7.83	A2β	1.43		3.81	2.54
KN	7.73	A3β	1.51		3.81	2.54
**α(i)-β(i+3)**						
A1α	3.82	C1β1	2.61		3.95	2.63
A4α	4.02	A2β	1.45		2.94	1.96
A3β	1.50	C1α	4.45		4.50	3.00
A5α	4.16	A6β	1.40		4.06	2.71
C3α	4.22	A5β	1.54		3.75	2.50
**C-terminal-amide**						
C-N1	6.91	C-N2	7.20		2.02	1.35
**TMB related**						
C2β2	2.79	TMB-aro1	6.97		4.16	2.77
C2β1	2.46	TMB-aro3	6.69		4.32	2.88
C3β1	2.63	TMB-aro2	6.60		3.71	2.47
TMB-α1-2	3.81	TMB-aro1	6.96		2.77	1.84
TMB-α3-2	3.64	TMB-aro1	6.96		3.03	2.02
TMB-α2-2	3.71	TMB-aro3	6.70		3.79	2.53
TMB-α3-2	3.64	TMB-aro3	6.71		4.05	2.70
TMB-α2-1	3.56	TMB-aro3	6.69		3.51	2.34
TMB-α3-1	3.51	TMB-aro3	6.70		3.01	2.01
C2α	3.46	TMB-aro3	6.70		4.18	2.79
TMB-α1-2	3.81	TMB-aro2	6.60		4.50	3.00
TMB-α2-2	3.71	TMB-aro2	6.59		3.26	2.17
TMB-α2-1	3.56	TMB-aro2	6.59		4.11	2.74
TMB-α1-1	3.50	TMB-aro2	6.58		3.33	2.22
TMB-α1-1	3.50	C1β2	3.22		3.46	2.31
TMB-α2-2	3.70	C3β1	2.62		4.50	3.00
TMB-α1-1	3.51	C1β1	2.61		3.13	2.09
TMB-α2-1	3.56	C3β1	2.62		4.61	3.08
TMB-α3-2	3.65	C2β2	2.78		4.26	2.84
TMB-α3-1	3.51	C2β2	2.80		4.14	2.76
TMB-α3-2	3.63	C2β1	2.46		5.17	3.44

TMB-α3-1	3.52	C2β1	2.46		4.10	2.73
C1N	7.71	TMB-aro2	6.59		4.47	2.98
C2N	7.67	TMB-aro3	6.70		4.52	3.02
TMB-aro2	6.60	C1α	4.44		2.70	1.80
TMB-aro2	6.60	C1β2	3.23		4.33	2.89
TMB-aro2	6.60	A3β	1.50		3.85	2.56
TMB-aro3	6.70	C2β2	2.79		4.61	3.08
TMB-aro3	6.70	C3β1	2.63		5.17	3.44
TMB-aro1	6.97	C1β1	2.60		5.02	3.34
TMB-aro1	6.97	C2β1	2.47		5.27	3.51

Supplementary Table 6 Coupling constants and corresponding Φ of bicyclo 12-mer calculated from equation[5] {$J(\theta) = 6.98 * (\cos(\theta))^2 - (1.38*\cos(\theta)) + 1.72$, where $\theta = |\Phi-60|$}.

**Residue**	$^3J_{NH\text{-}aH}$ **(Hz)**	**Φ (°)**
W		
C2	6.8	*inconclusive*
D	8.8	-96 ± 30
A1		
A4	4.8	-65 ± 30
C3	5.2	-68 ± 30
C1	5.2	-68 ± 30
A2	5.2	-68 ± 30
A5	5.5	-70 ± 30
A3	5.3	-69 ± 30
K	5.2	-68 ± 30
A6		

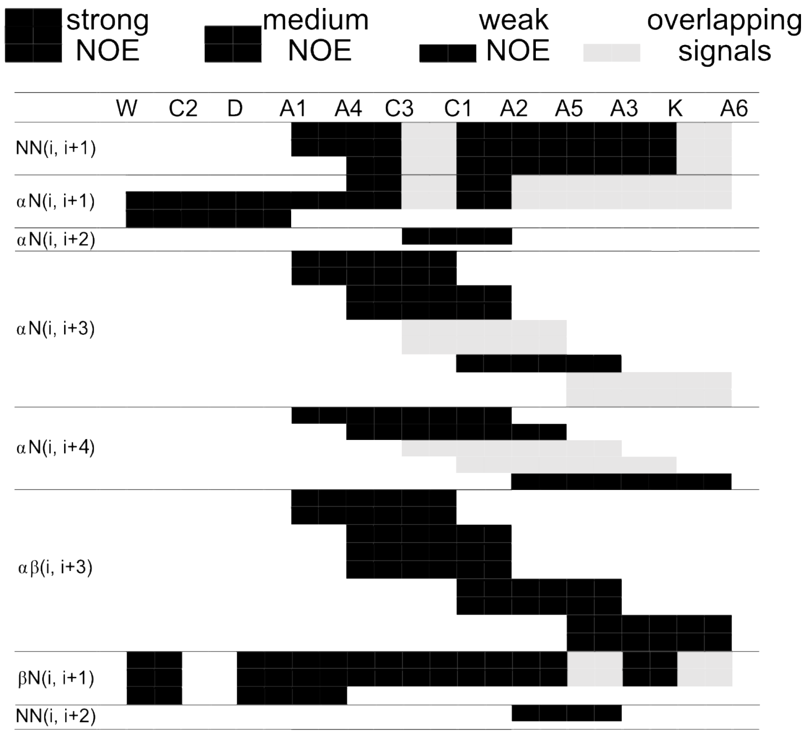

Supplementary Figure 37 Characteristic cross-residue ROE in bicyclo 12-mer.

W-[C2-D-A1-A4-C3-C1]$_{cyclo}$-A2-A5-A3-K-A6-NH$_2$

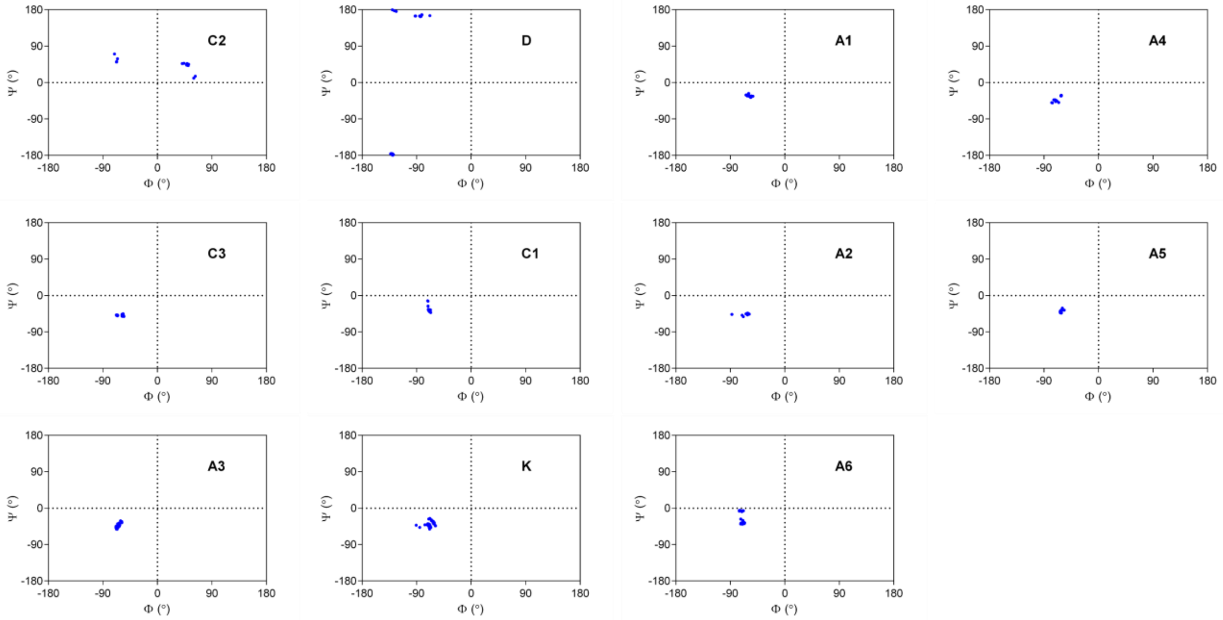

Supplementary Figure 38 Dihedral angles (Φ,ψ) of residues in low energy conformers of bicyclo 12-mer.

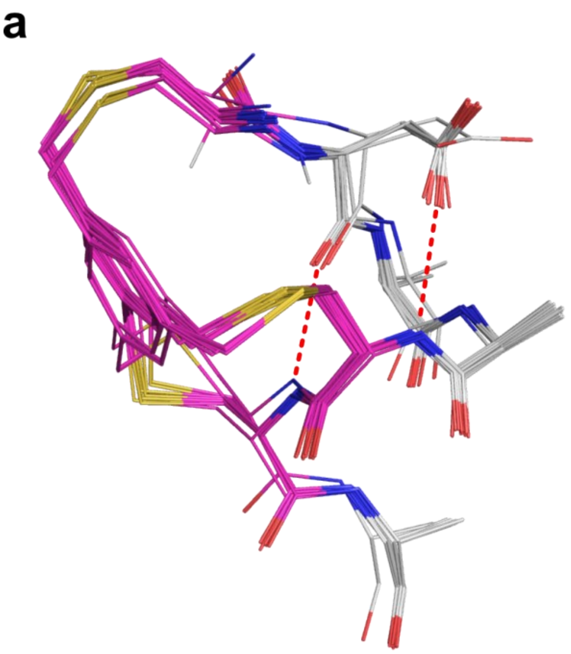

Supplementary Figure 39 Cluster 1 (left) and 2 (right) are different from χ angle in *Ncap* Asp sidechain. **a.** Cluster 1 (29/50) : ASX motif; **b**. Cluster 2 (21/50): Sidechain of Asp points outward helical backbone, potentially forming *H*-bond to its amide.

W-[C2-D-A1-A4-C3-C1]$_{cyclo}$-A2-A5-A3-K-A6-NH_2

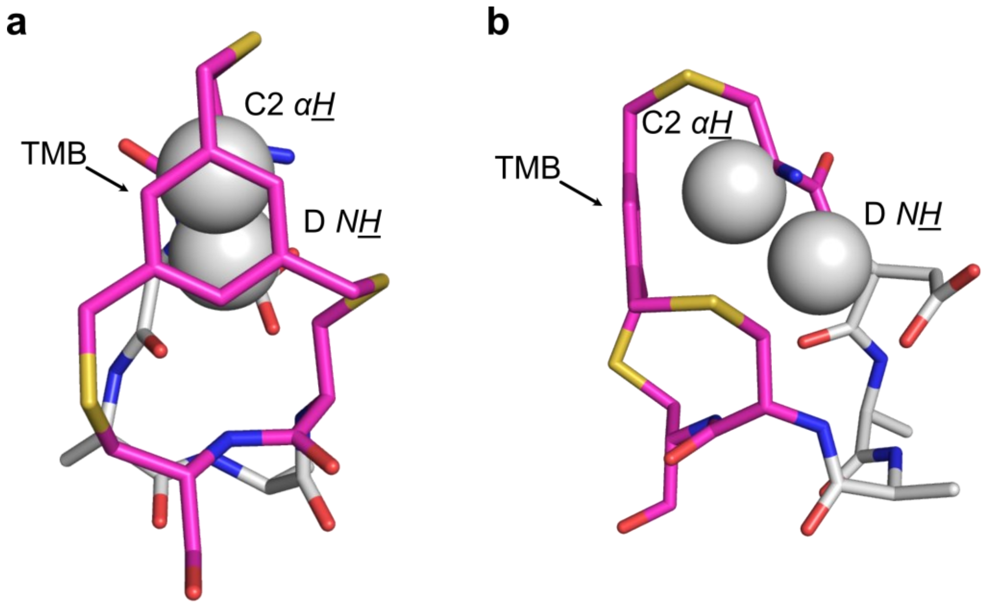

Supplementary Figure 40 The Impacts of Shielding Effect from TMB to Nearby Hs a. Front view of benzene ring and the two affected *H*s. **b.** Side view of benzene ring and the two affected *H*s. Two *H*s were found to have 'abnormally' smaller chemical shifts than their colleagues. One is α*H* of C2 (3.47 ppm). The chemical shifts of α*H*s of the other two Cys is 4.21 and 4.44 ppm, respectively. An upfield shift of around 0.8 ppm indicates this α*H* is likely shielded by the TMB group. The other one is amide *NH* of D (7.24 ppm). The average chemical shift of the other amide *NH*s (except terminal amide) is 7.87 ppm. An upfield shift of 0.6 ppm suggests this amide *NH* is also likely shielded by the TMB group, but probably by a smaller impact compared to the α*H* of C2. These observations matches the 3D conformations derived from NMR constraints. Supplementary Figure 40a shows the front view of the lowest energy conformer, both *H*s are right behind the TMB group. In the side view at Supplementary Figure 40b, α*H* of C2 is closer to the TMB group than *NH* of D hence have stronger shielding effect and more upfield shifts in NMR spectrum.

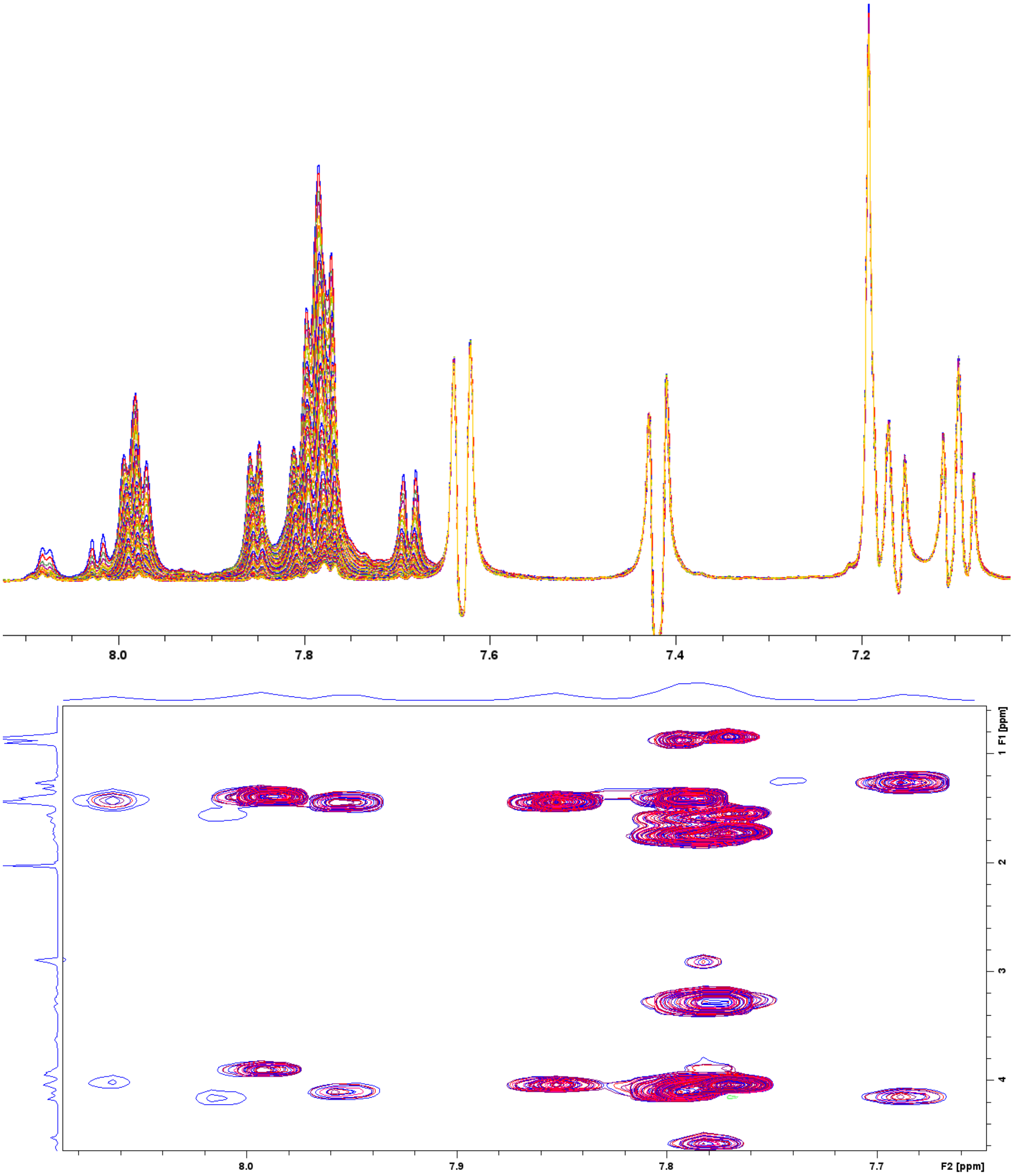

Supplementary Figure 41 1D H^1 (top) and 2D H^1-H^1 TOCSY (bottom) spectra of LDLL 12-mer for 12 hours in *H-D* exchange experiments.

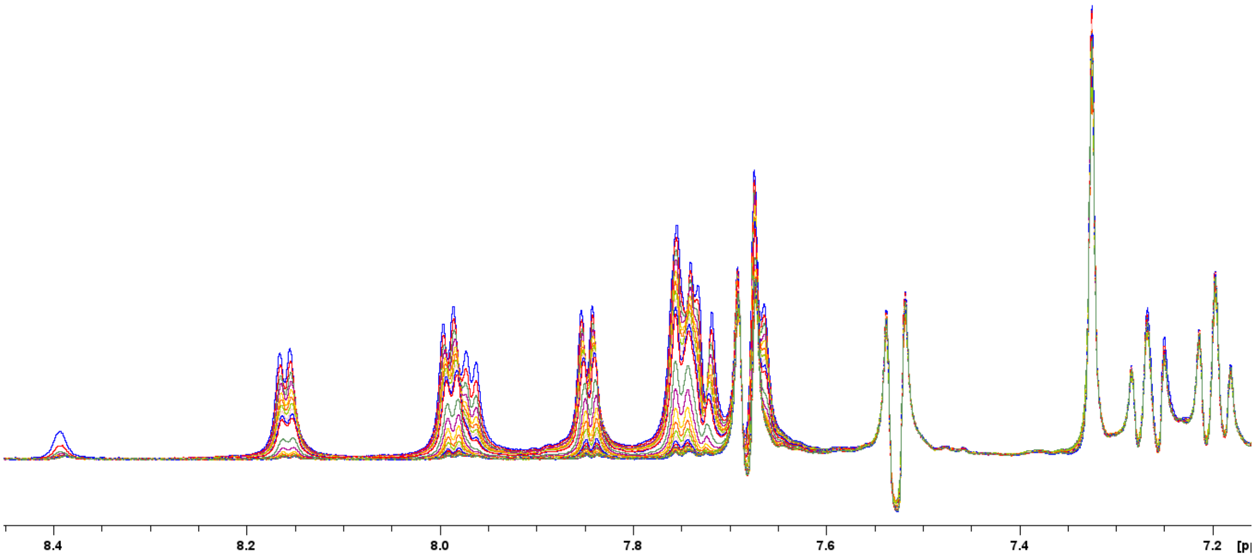

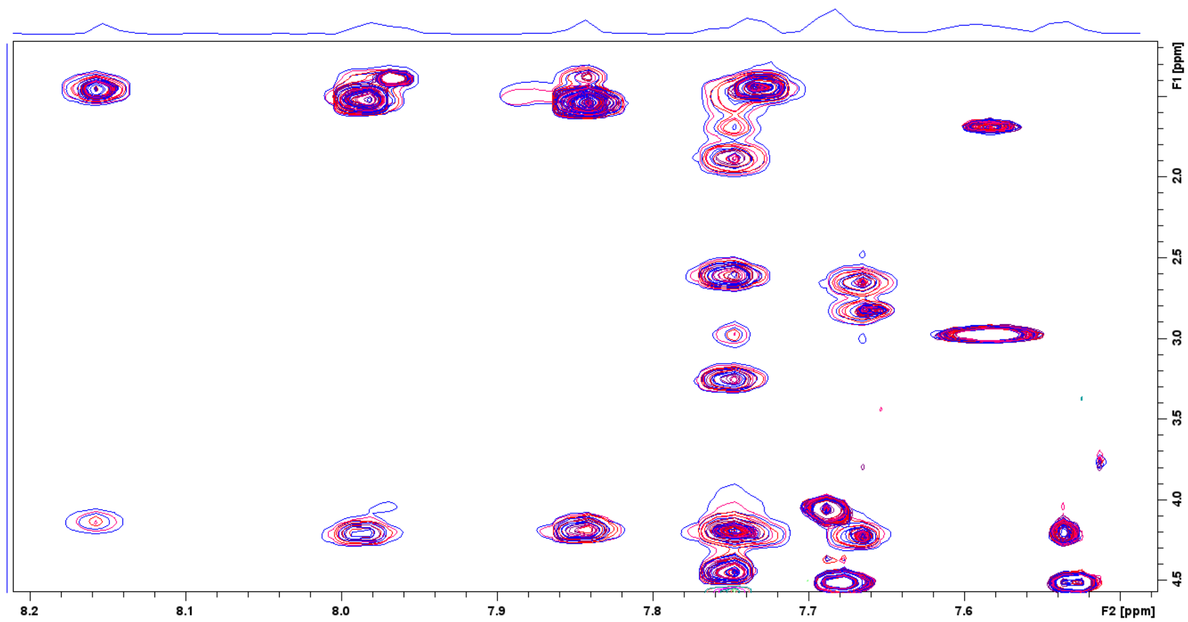

Supplementary Figure 42 1D H[1] (top) and 2D H[1]-H[1] TOCSY (bottom) spectra of bicyclo 12-mer for 12 hours in *H-D* exchange experiments.

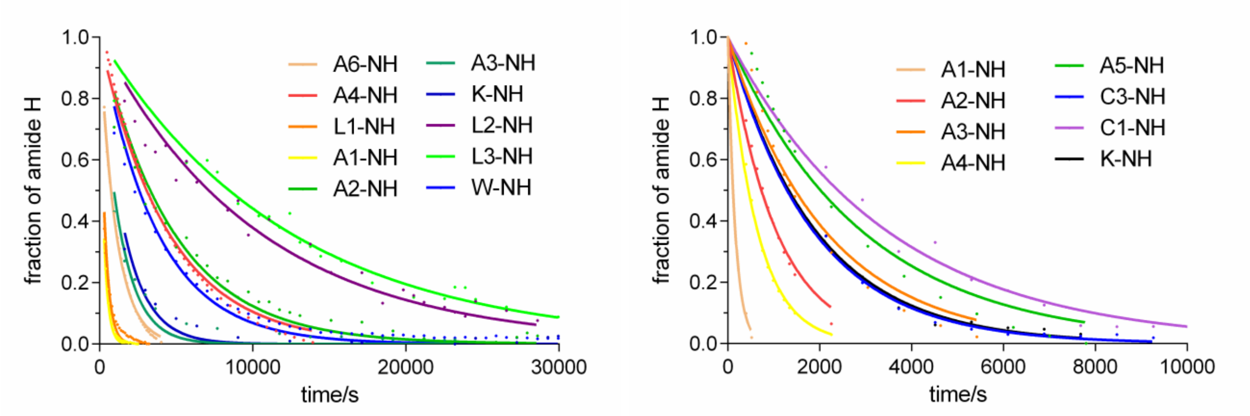

Supplementary Figure 43 ***H-D*** **exchange plots for backbone amide protons in LDLL 12-mer (left) and bicyclo 12-mer (right).**

Supplementary Table 7 Summary of ***H-D*** **exchange data[6] for LDLL 12-mer and bicyclo 12-mer.**

	N'	*Ncap*	*N1*	*N2*	*N3*	*N4*	*N5*	*N6*	*N7*	*N8*	*N9*
LDLL 12-mer	L	D	A	A	L	L	A	A	A	K	A
H/D rate constant × 10^{-4} (s^{-1})	28.27		35.67	7.38	0.97	0.82	2.25		2.09	6.2	9.29
protection factor (log k_{ch}/k_{ex})	0.30		1.30	1.66	1.96	1.83	1.96		2.20	1.69	1.68
stabilization, -ΔG (kcal/mol)	0.01		1.79	2.30	2.73	2.54	2.73		3.07	2.35	2.32
Bicyclo 12-mer	C	D	A	A	C	C	A	A	A	K	A
H/D rate constant × 10^{-4} (s^{-1})			61.98	15.45	5.38	2.89	9.49	3.43	4.72	5.22	
protection factor (log k_{ch}/k_{ex})			1.33	1.81	2.88	3.7	2.57	2.46	2.32	2.24	
stabilization, -ΔG (kcal/mol)			1.81	2.51	4.02	5.17	3.58	3.43	3.24	3.12	

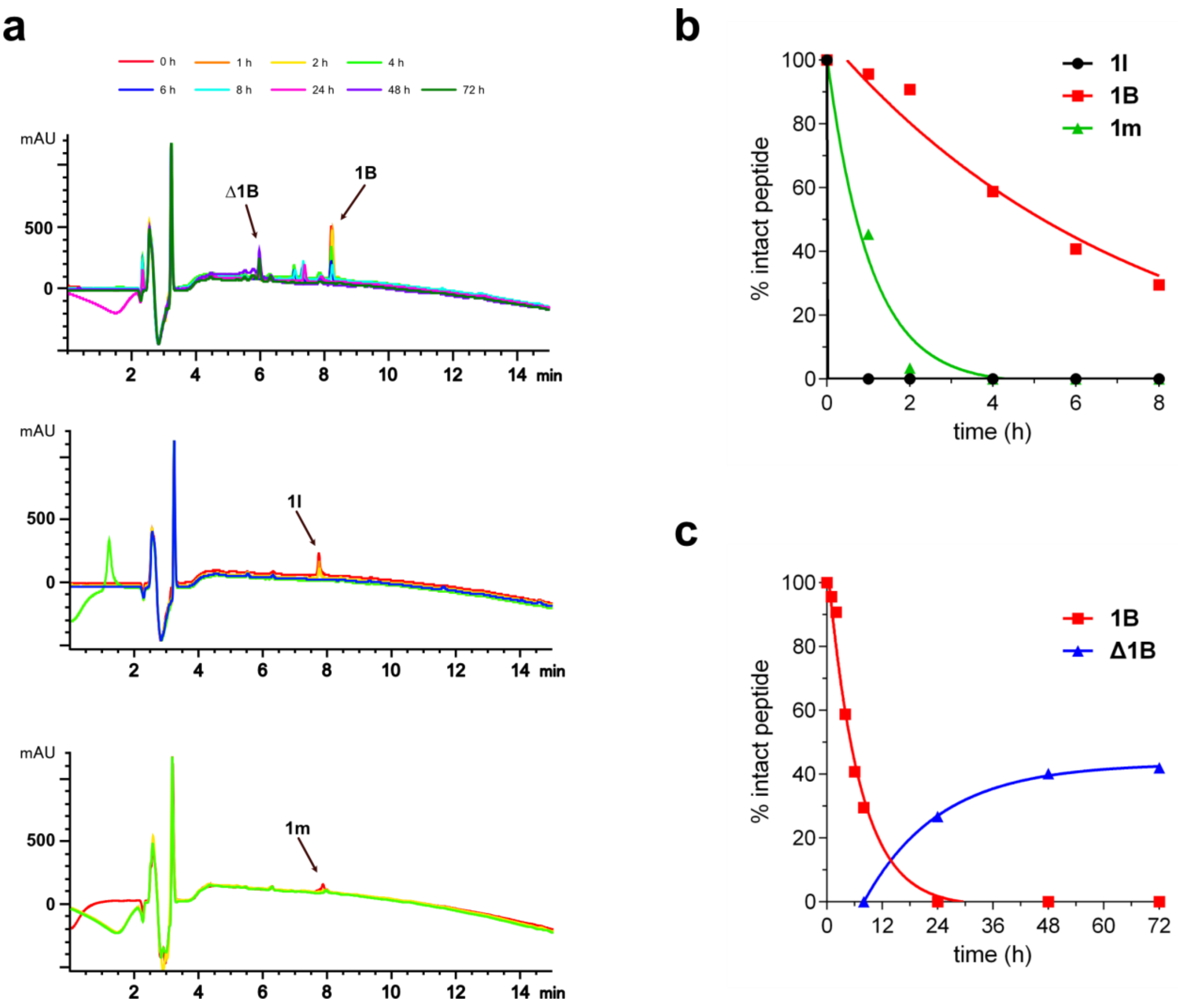

Supplementary Figure 44 Degradation Kinetic Curves and RP-HPLC Spectra of Series 1 Peptides (n=1). a. Overlaid RP-HPLC traces of **1B** from 0 to 72 hours, **1l** from 0 to 6 hours, and **1m** from 0 to 4 hours. **b**. Kinetic curves of series **1** peptides in 8 hours. **c**. Δ**1B** formed as **1B** was exposed to 25% serum solution. The raw data of kinetic curves showed how fast tested peptides degraded over time, and also presented the increase of final degradation product of **1B**, which we named as Δ**1B**. The percentage of Δ**1B** was calculated by comparing its peak areas with initial peak areas (0 h) of **1B**. This is not necessarily accurate because the cleavage product should have a smaller absorbance coefficient than the initial peptide, but it is probably enough to qualitatively show the kinetics and stability of the cleavage product. For linear peptides **1l** and **1m**, especially **1m**, we observed they were unstable, and will quickly self-degrade upon dissolving in aqueous solution. This made their (0h) peaks even smaller. Nevertheless, due to the effect of self-degradation and protease degradation, both peptides disappear within 4 hours with no obvious remnants observed. mAU refers to mini Arbitrary Unit.

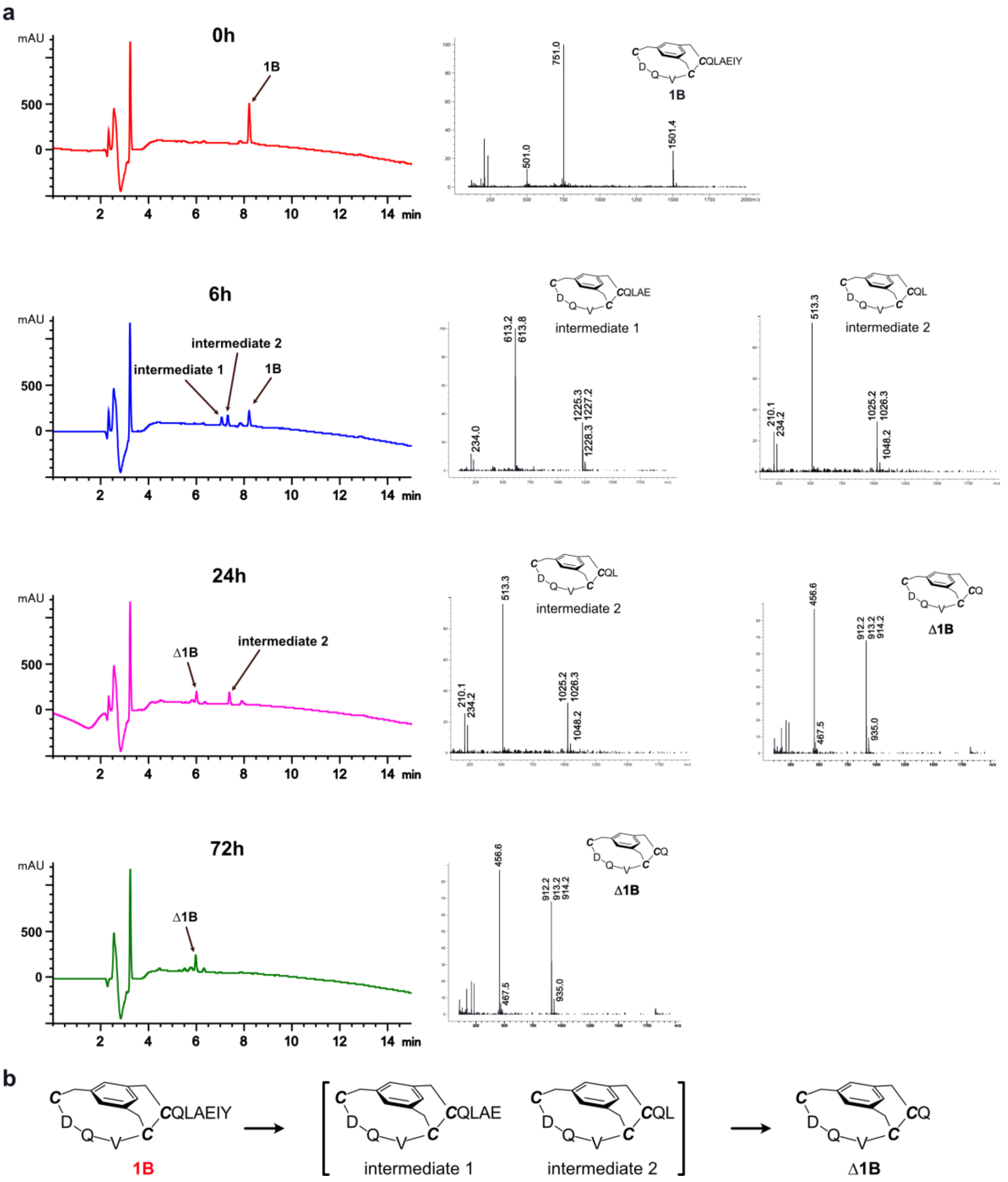

Supplementary Figure 45 Degradation products of 1B. For **1B**, several intermediates were observed and finally converted to Δ**1B**. Δ**1B** did not show obvious degradation from 48 to 72 hours indicating high serum stability of the bicyclic *N*-cap fragment. Chemical formula of degradation product can be speculated by mass spectra obtained from LCMS. **a**. Speculated chemical structures, MS spectra (from LCMS) and RP-HPLC traces of intermediates and Δ**1B** at 0, 6, 24 and 72 h. **b**. Speculated degradation process of **1B**. mAU refers to mini Arbitrary Unit.

.

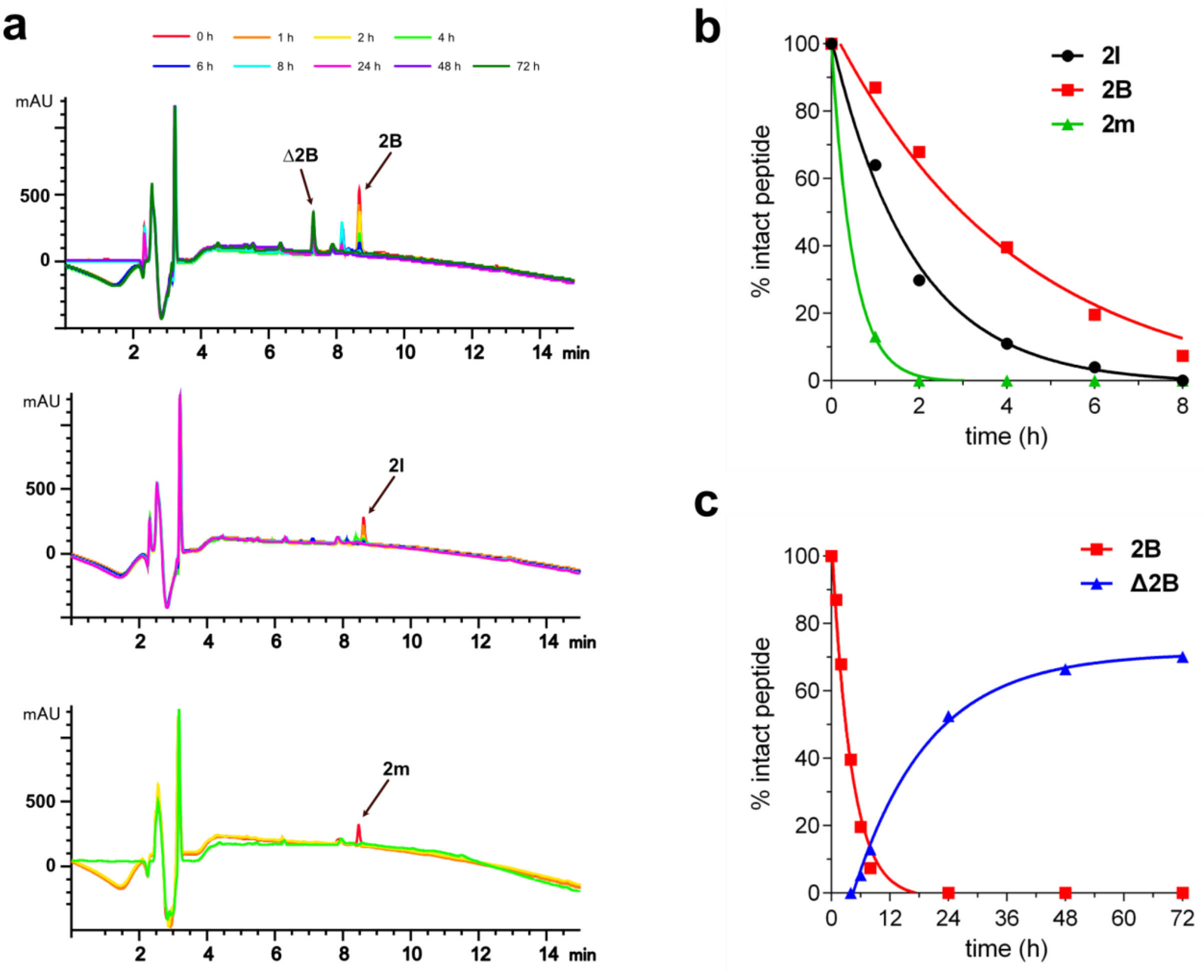

Supplementary Figure 46 Degradation Kinetic Curves and RP-HPLC Spectra of Series 2 Peptides (n=1). a. Overlaid RP-HPLC traces of **2B** from 0 to 72 hours, **2l** from 0 to 24 hours and **2m** from 0 to 4 hours. **b**. Kinetic curves of series **2** peptides in 8 hours. **c**. ∆**2B** formed as **2B** was exposed to 25% serum solution. The raw data of kinetic curves showed how fast tested peptides degraded over time, and also presented the increase of final degradation product of **2B**, which we named as ∆**2B**. The percentage of ∆**2B** was calculated by comparing its peak areas with initial peak areas (0 h) of **2B**. This is not necessarily accurate because the cleavage product should have a smaller absorbance coefficient than the initial peptide, but it is probably enough to qualitatively show the kinetics and stability of the cleavage product. For the linear peptides **2l** and **2m**, no obvious remnants were found and it showed plain curves with several minor background peaks after 6 hours. mAU refers to mini Arbitrary Unit.

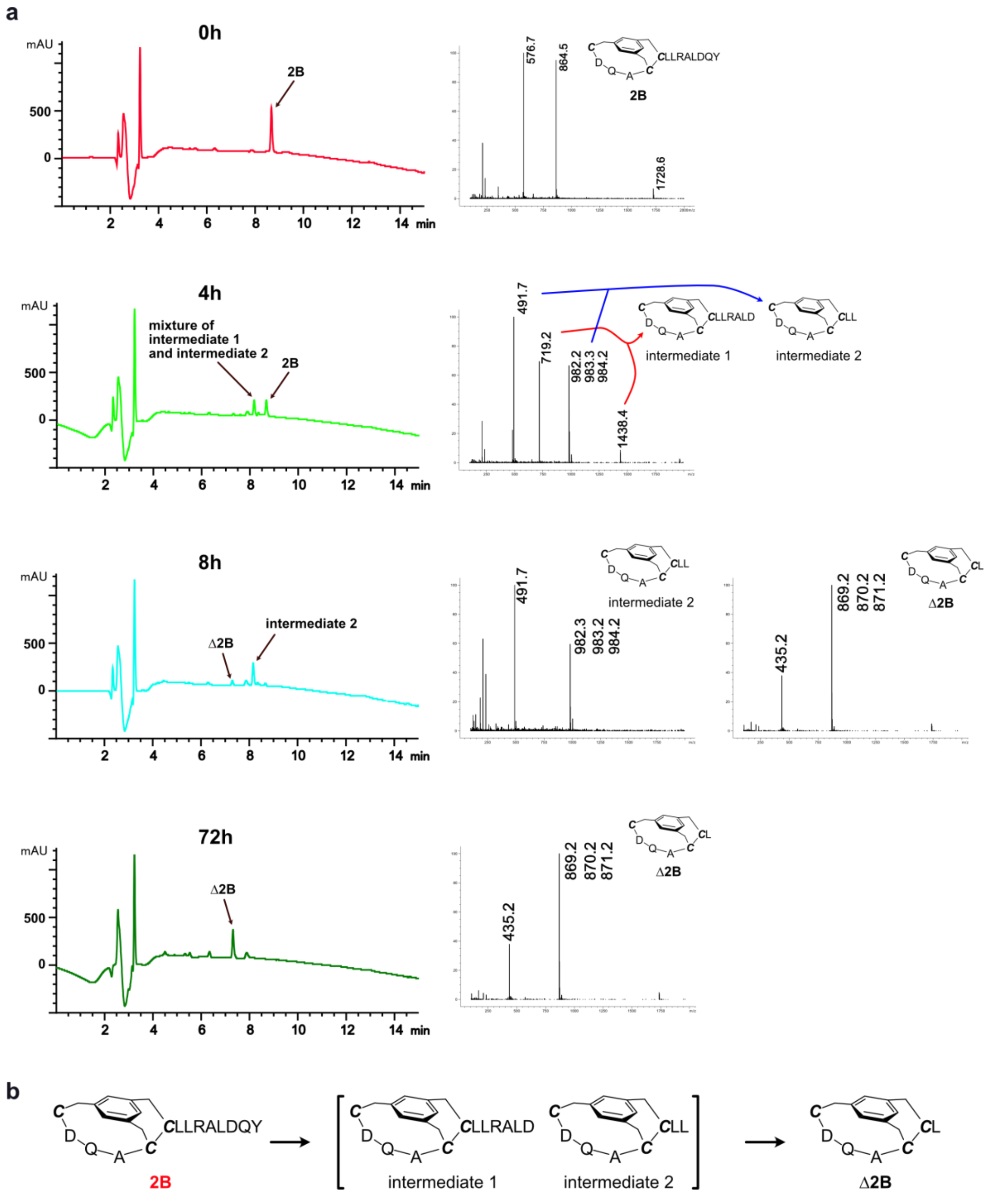

Supplementary Figure 47 Degradation products of 2B. For **2B**, several intermediates were observed and finally converted to **Δ2B**. **Δ2B** did not show obvious degradation from 48 to 72 hours indicating high serum stability of the bicyclic *N*-cap fragment. Chemical formula of degradation product can be speculated by mass spectra obtained from LCMS. **a.** Speculated chemical structures, MS spectra (from LCMS) and RP-HPLC traces of intermediates and **Δ2B** at 0, 4, 8 and 72 h. **b.** Speculated degradation process of **2B**. mAU refers to mini Arbitrary Unit.

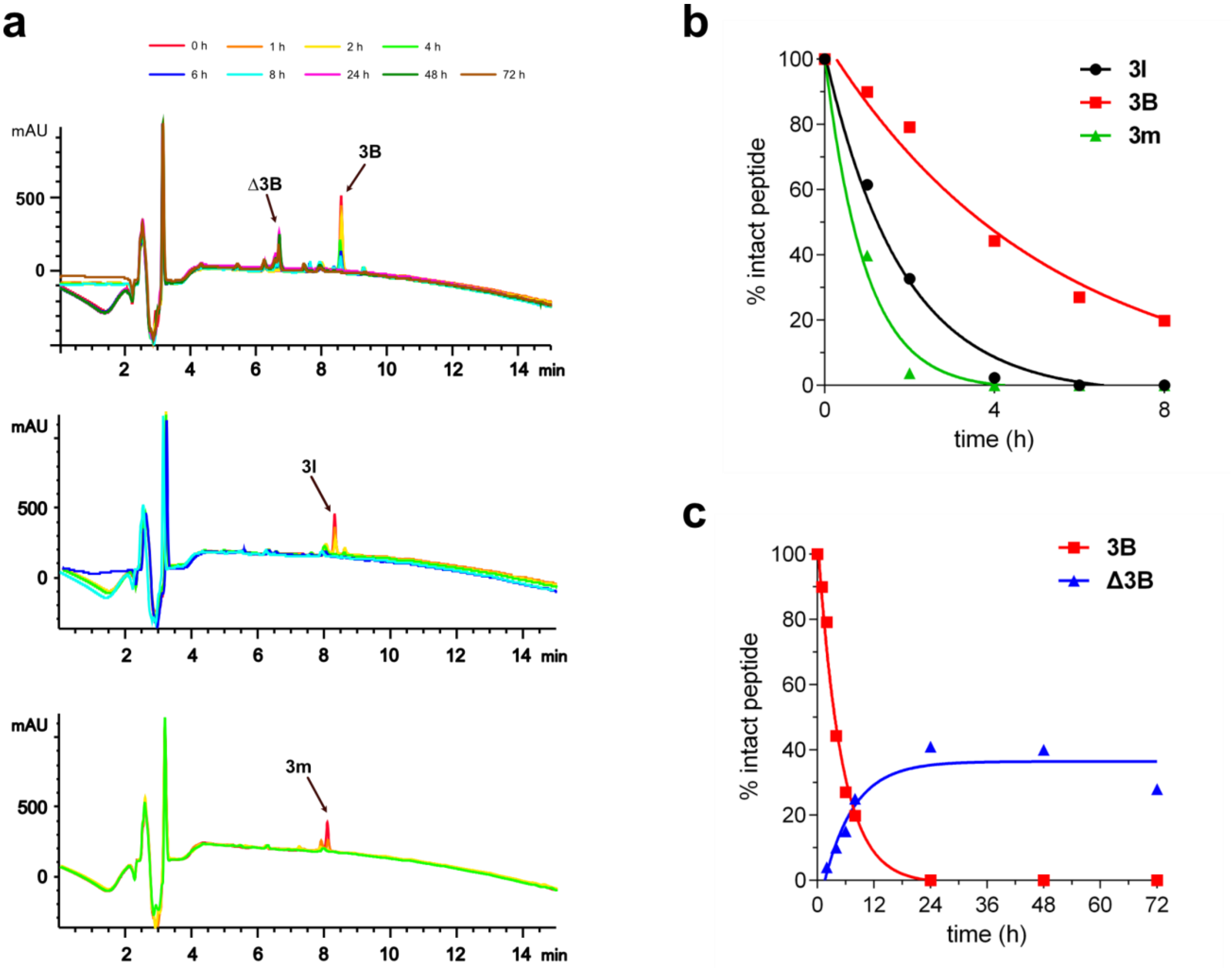

Supplementary Figure 48 Degradation Kinetic Curves and RP-HPLC Spectra of Series 3 Peptides (n=1). a. Overlaid RP-HPLC traces of **3B** from 0 to 72 hours, **3l** from 0 to 8 hours and **3m** from 0 to 4 hours. **b**. Kinetic curves of series **3** peptides in 8 hours. **c**. Δ**3B** formed as **3B** was exposed to 25% serum solution. The raw data of kinetic curves showed how fast tested peptides degraded over time, and also presented the increase of degradation products of **3B**. Contrary to **1B** and **2B**, **3B** showed a stable intermediate, Δ**3B**, which then slowly degraded to other smaller peptides, for example, Δ**3B'**. The percentage of degradation products was calculated by comparing its peak areas with initial peak areas (0 h) of **3B**. This is not necessarily accurate because the cleavage product should have a smaller absorbance coefficient than the initial peptide, but it is probably enough to qualitatively show the kinetics and stability of the cleavage product. For linear peptides **3l** and **3m**, no obvious remnants were found and it showed plain curves with several minor background peaks after 8 hours. mAU refers to mini Arbitrary Unit.

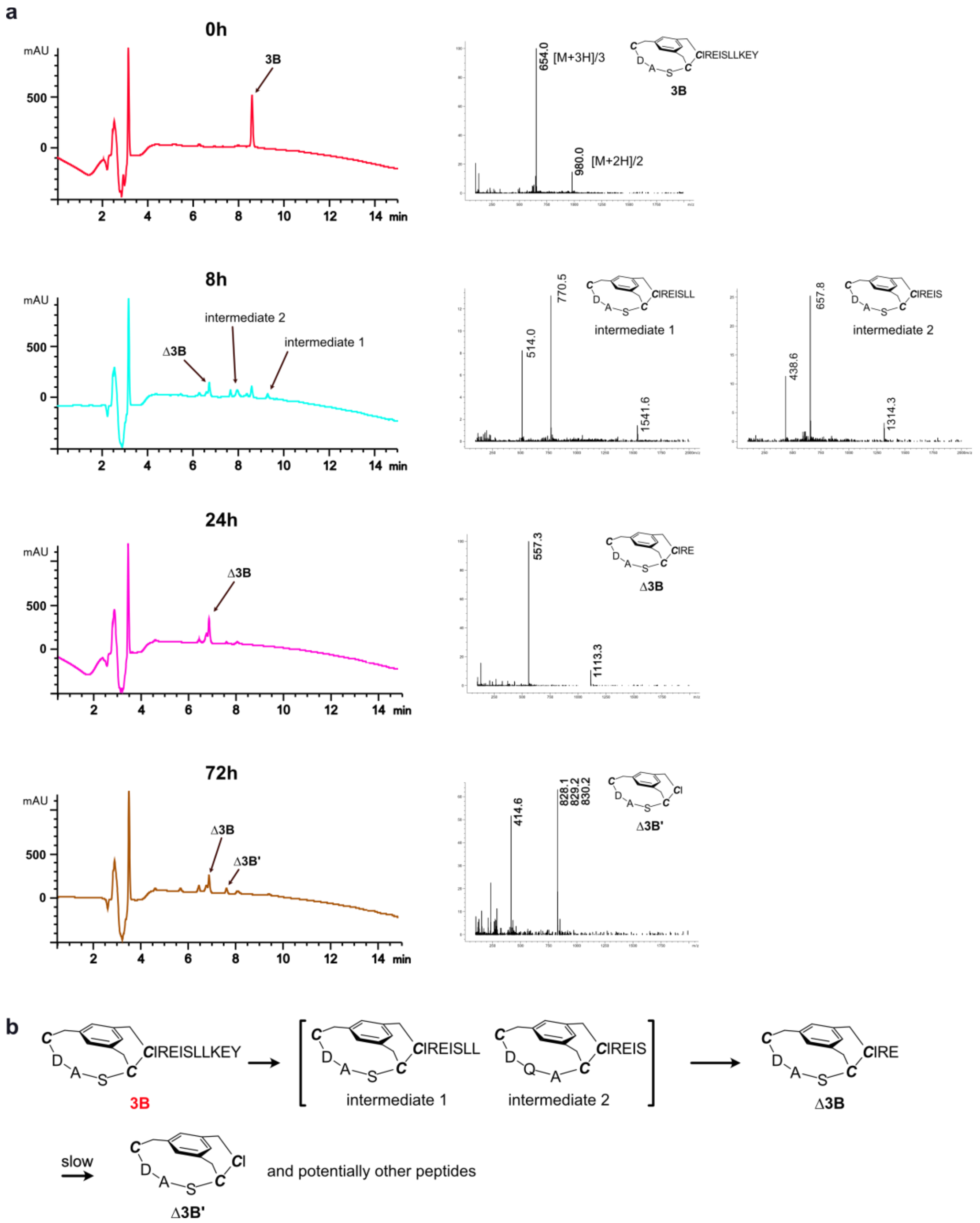

Supplementary Figure 49 Degradation products of 3B. For **3B**, it first primarily degraded to a stable intermediate, **Δ3B**, within 24 hours. **Δ3B** is highly stable in 25% human serum and then slowly degraded to other smaller peptides since 48 hours. All degradation products contained bicyclic fragments indicating high serum stability of the *N*-cap fragment. Chemical formula of degradation product can be speculated by mass spectra obtained from LCMS. **a.** Speculated chemical structures, MS spectra (from LCMS) and RP-HPLC traces of intermediates, **Δ3B** and **Δ3B'** at 0, 8, 24 and 72 h. **b.** Speculated degradation process of **3B**. mAU refers to mini Arbitrary Unit.

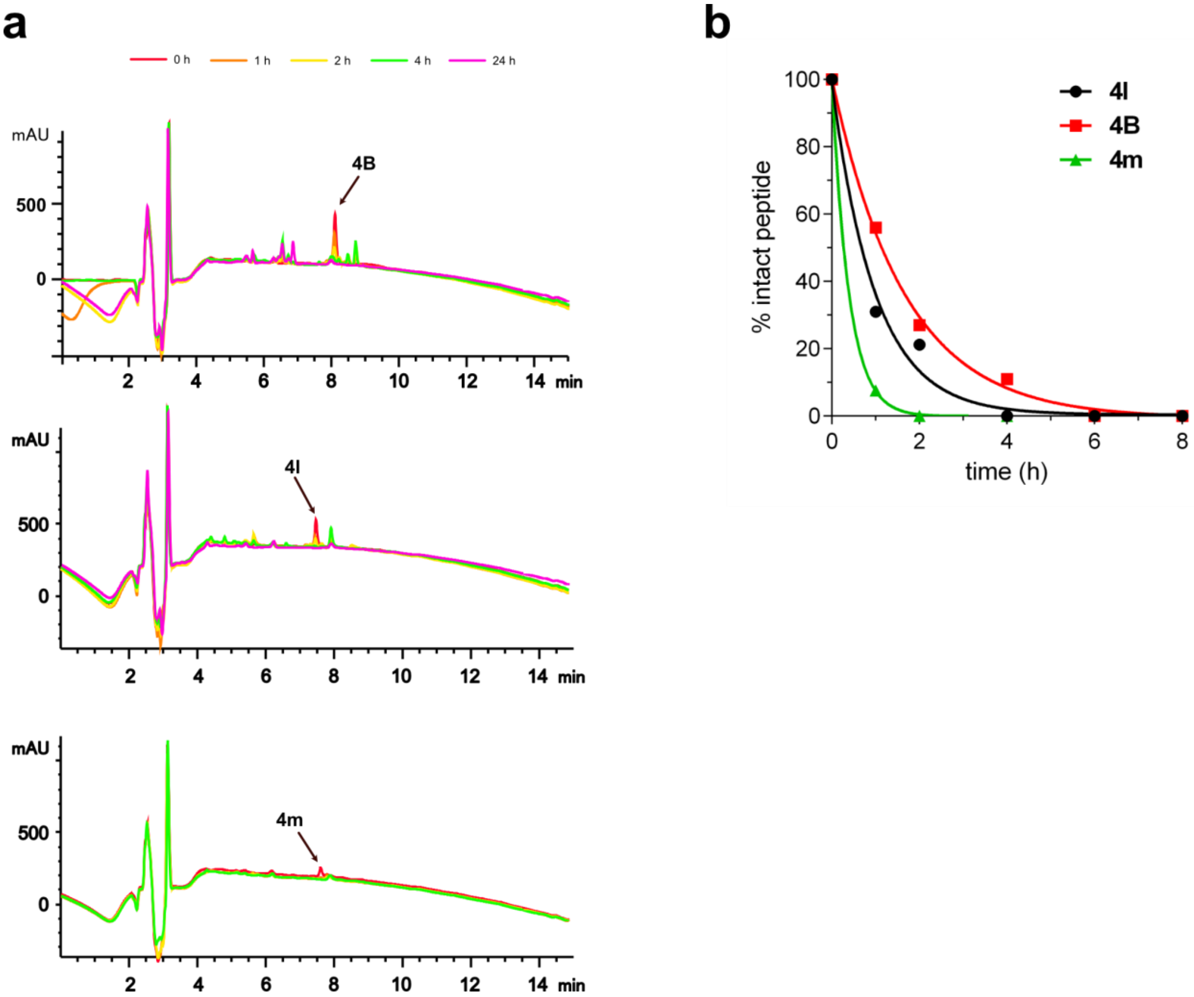

Supplementary Figure 50 Degradation Kinetic Curves and RP-HPLC Spectra of Series 4 Peptides (n=1). a. Overlaid RP-HPLC traces of **4B** from 0 to 24 hours, **4l** from 0 to 24 hours and **4m** from 0 to 4 hours. **b**. Kinetic curves of series **4** peptides in 8 hours. The raw data of kinetic curves showed how fast tested peptides degraded over time, and also presented the increase of degradation products of **4B**. **4B** was a long peptides with 25 amino acids. It yielded plenty of degradation intermediates without a dominant one, so it was complicated to track them and conclude a potential process of degradation as we have done for previous shorter peptides. Therefore, for **4B** only the degradation products in 24 hours were carefully analyzed by LCMS**.** For linear peptides **4l** and **4m**, no obvious remnants were found and it showed plain curves with several minor background peaks after 24 hours. mAU refers to mini Arbitrary Unit.

a

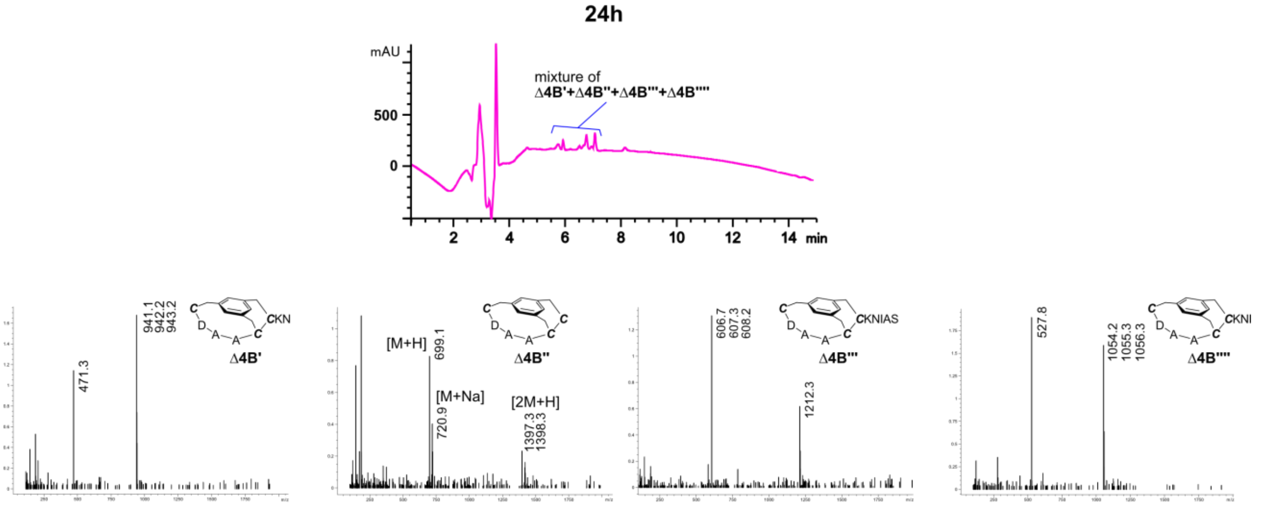

b

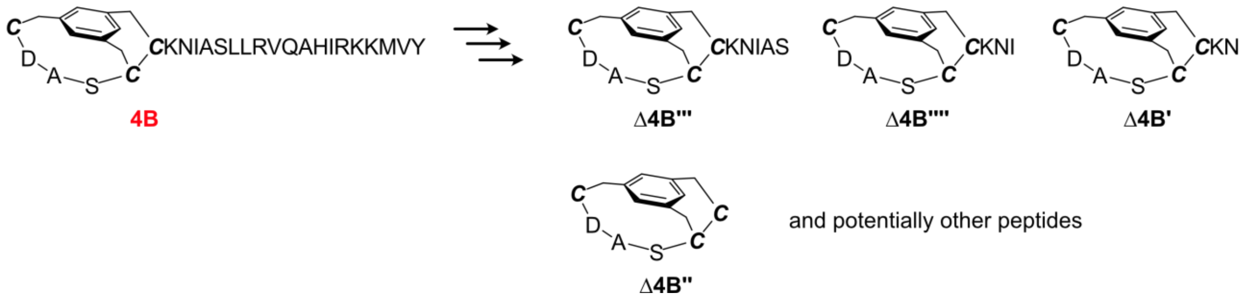

Supplementary Figure 51 Degradation products of 4B For **4B**, it degraded to plenty of intermediates, and after 24 hours a few short peptides were more abundant than the others. It was not accurate to assign shorter peptides from peaks in LCMS to those in analytical HPLC, because none was dominant. Therefore, four intermediates observed in LCMS, were presented in the order of decreased polarity (increased retention time). They should correspond to the cluster of peaks in analytical HPLC **a.** Speculated degrading intermediates at 24 h. **b.** Degrading products of **4B** at 24 h. mAU refers to mini Arbitrary Unit.

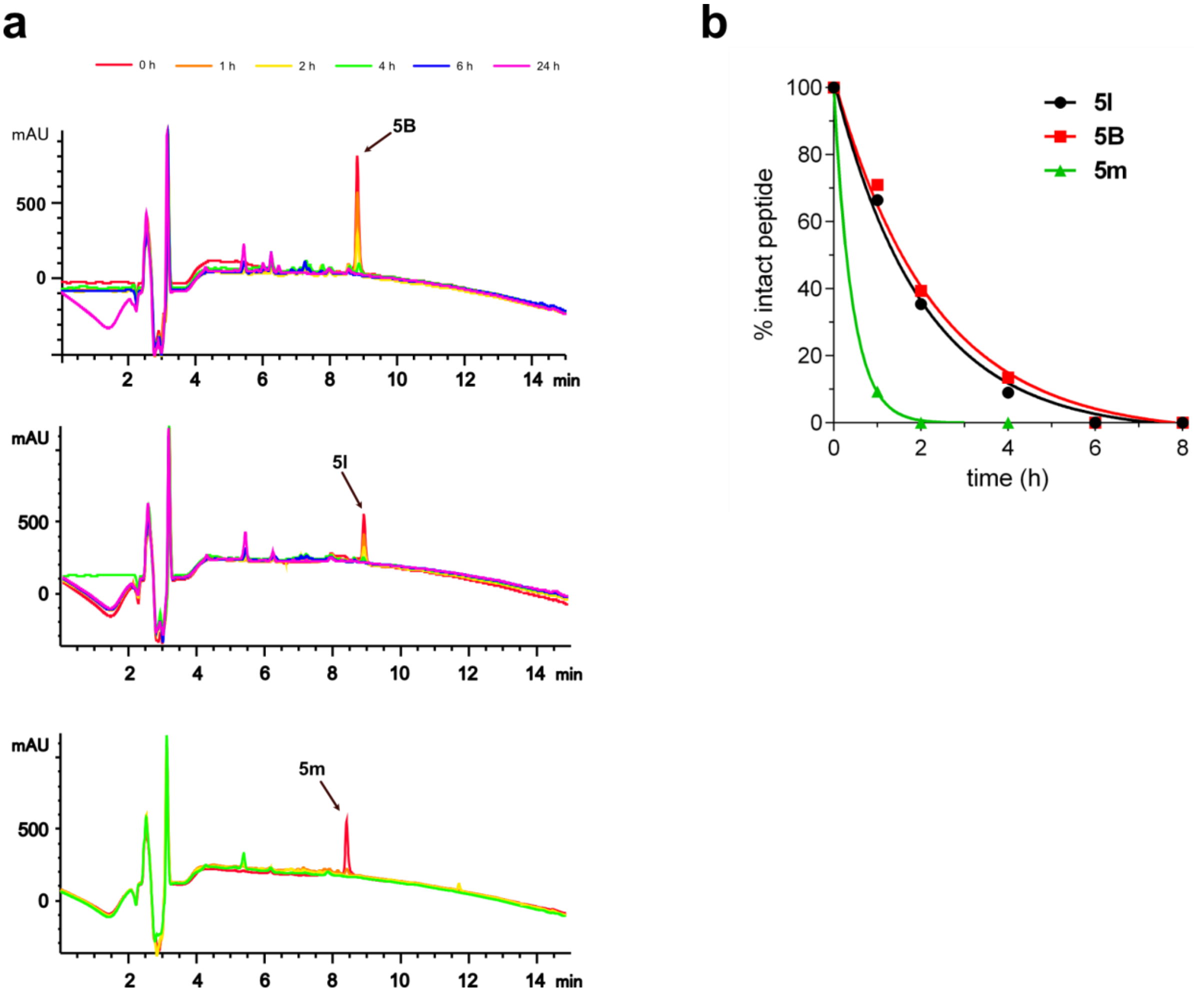

Supplementary Figure 52 Degradation Kinetic Curves and RP-HPLC Spectra of Series 5 Peptides (n=1). a. Overlaid RP-HPLC traces of **5B** from 0 to 24 hours, **5l** from 0 to 24 hours and **5m** from 0 to 4 hours. **b**. Kinetic curves of series **5** peptides in 8 hours. The raw data of kinetic curves showed how fast tested peptides degraded over time, and also presented the increase of degradation products of **5B**. **5B** was a long peptides with 28 amino acids. It yielded plenty of degradation intermediates without a dominant one, so it was complicated to track them and conclude a potential process of degradation as we have done for previous shorter peptides. Therefore, for **5B** only the degradation products in 24 hours were carefully analyzed by LCMS. For linear peptides **5l** and **5m**, after 4 hours the original peptides have been completely degraded, and only a peak at 5.3 min was observed for both peptides. It seemed stable after 24 hours. It was a random case because none of previous linear peptides showed any significant remnants after 24 hours. mAU refers to mini Arbitrary Unit.

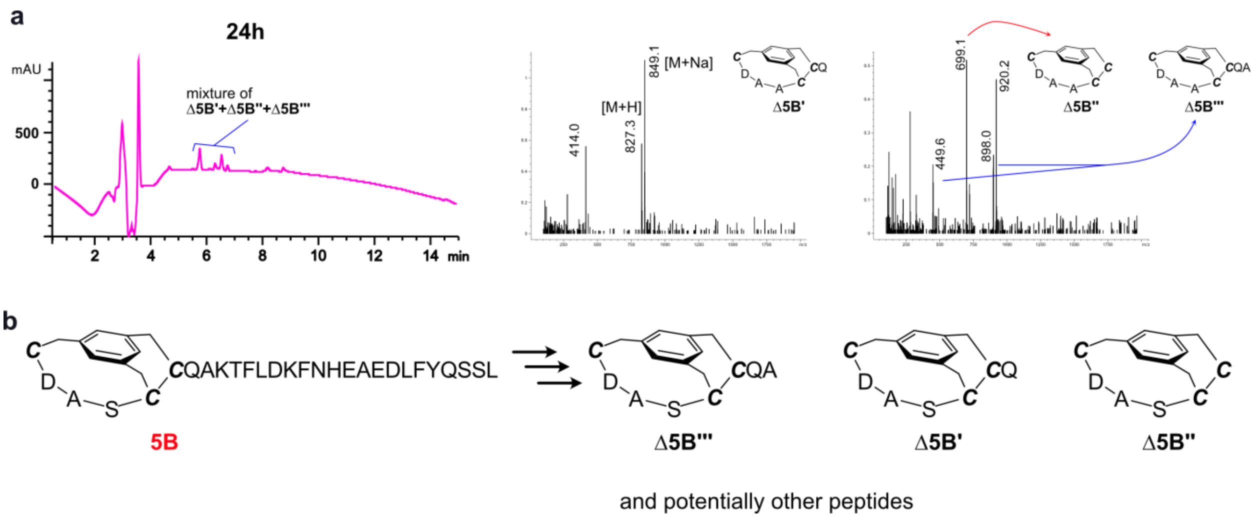

Supplementary Figure 53 Degradation products of 5B For **5B**, it degraded to plenty of intermediates, and after 24 hours a few short peptides were more abundant than the others. It was not accurate to assign shorter peptides from peaks in LCMS to those in analytical HPLC, because none was dominant. Therefore, three intermediates observed in LCMS, were presented in the order of decreased polarity (increased retention time). They should correspond to the cluster of peaks in analytical HPLC. **a.** Speculated degrading intermediates at 24 h. **b.** Degrading products of **5B** at 24 h. mAU refers to mini Arbitrary Unit.

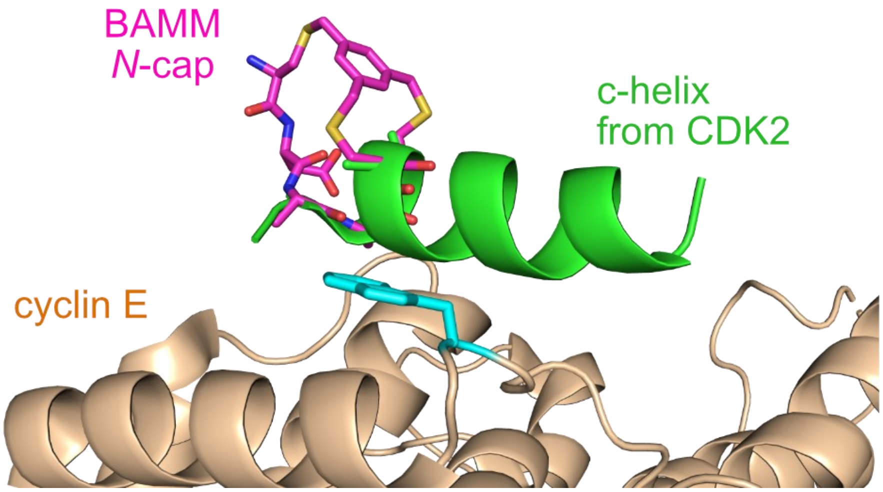

Supplementary Figure 54 The Crystal Structure of CDK2·Cyclin E CDK2•cyclin E from PDB 1W98 showing a tryptophan in cyclin E directly impacting the C-helix of CDK2. The putative position of BAMM N-cap in magenta.

Supplementary Table 8 Sequences and binding affinities of fluorescent labeled peptides.

Label	Sequence	K_d (μM)
**3lf**	FITC-(β-Ala)$_3$-ASTAIREISLLKEY	\
**3Bf**	FITC-(β-Ala)$_3$-{CDASCC}$_{cyc}$IREISLLKEY	4.6 ± 2.4
**3mf**	FITC-(β-Ala)$_3$-C*DASC*C*IREISLLKEY	\

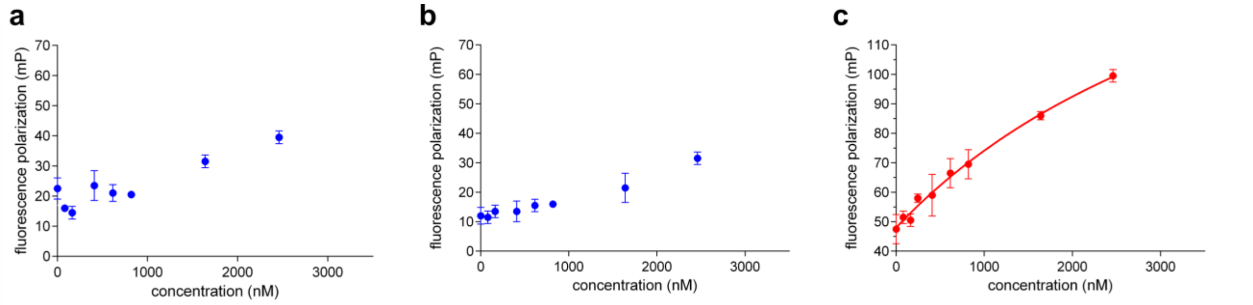

Supplementary Figure 55 Raw data of fluorescence polarization assays for **a. 3lf**, **b. 3mf**, and **c. 3Bf**. FITC labeled series **3** peptides, **3lf**, **3mf** and **3Bf** were prepared for assays associated with fluorescence polarization (n=1 independent experiment, error bars show deviations between duplicate wells of samples at the same concentration in the experiment). Their sequences and calculate binding affinities are in Supplementary Table 8. With limit amount of cyclin E, the highest final concentration of the protein was set as 2.4 μM in the assays. None of the three peptides were completely saturated. However, the BAMM mimic was more closer to the top boundary and the equation was able to give an estimate value. Both linear peptides were still far away from the top boundary, so the functions was unable to calculate K_d values for them. Nevertheless, both linear peptides showed less affinity than the BAMM capped one.

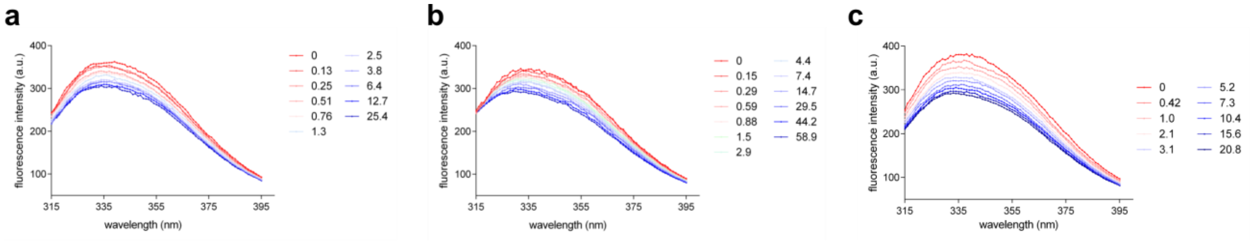

Supplementary Figure 56 Representative fluorescence spectra for cyclin E with different concentrations of **a. 3Bf**, **b. 3lf**, and **c. 3mf**. Two independent experiments were done and gave similar results. a.u. refers to arbitrary unit.

Supplementary Table 9 Comparison of HBS, peptidic strap and BAMM on mimicking natural helices (from bold proteins in PPI column). Multiple papers have published *N*-capped helical mimics using strategy such as HBS and peptidic strap.[7-12] The corresponding protein-protein interactions (PPI), capping strategy, sequences of linear and capped peptides, their K_d values, and folds of improvement are recorded below. Here for capped sequences, only ones most similar to the original linear peptides were considered; those further optimized by mutations of middle residues were not included, because the enhanced affinity in these peptides were not caused by capping. Comparing with linear, wild-type peptides, modern *N*-capping strategies generally improve binding by 1~10 folds, and in some cases the binding was slightly weaker than the linear ones. The improvement was also illustrated in $\Delta\Delta G$ where a higher value suggests a larger enhancement of binding. Overall, the K_d improvement in the illustrated case in this paper, CDK2/cyclin E, was comparable to the best case here (RAS/SOS), considering both folds of improvement and change of ΔG.

PPI	Capping Strategy	Linear Sequence	Linear Kd (μM)	Capped Sequence	Capped Kd (μM)	Folds of Improvement	$\Delta\Delta G$ (kJ·mol^{-1})
Bak-BH3/**BcL-xL**	HBS	Ac-GQVGRQLAIIGDDINR	0.15 ± 0.02	[XQVG*]$_{cyc}$RQLAIIGDDINR	0.33 ± 0.05	0.5	-2.0
**HIV**/gp41	HBS	Ac-MTWMEWDREINNYT	37.4 ± 14.8	[XMTW*]$_{cyc}$MEWDREINNYT	46.6 ± 14.6	0.8	-0.6
**HIF**/p300	HBS	Ac-TAADCEYNAR	0.83 ± 0.05	[T*AAD*]$_{cyc}$CEYNAR	0.42 ± 0.04	2	1.7
**HIF**/p300	HBS	Ac-ELARALDQ	6.1 ± 0.3	[XELA*]$_{cyc}$RALDQ	0.69 ± 0.03	9	5.4
nucleotide-free RAS/**SOS**	HBS	Ac-FEGIYRLELLKAEEANK FITC	273 ± 9	[XFEG*]$_{cyc}$IYRLELLKAEEANKFITC	28 ± 5	10	5.7
**PERM**/ER-α	peptidic strap	FITC-(β-A)-HKILHRLLQ	0.099 ± 0.01	FITC-(β-A)-R(iso-D)IL(Dap)RLLQ	0.085 ± 0.02	1.2	0.38
**CDK2**/cyclin E	BAMM	Ac-ASTAIREISLLKEY	4.0 ± 0.9	[CDASCC]$_{cyc}$IREISLLKEY	0.50 ± 0.10	8	5.2

X: pent-4-enoic acid before coupling and olefin metathesis
T*: a derivative of pent-4-enoic acid, where the side chain of Thr was at the α position of pent-4-enoic acid
G*, W*, D*, A*: *N*-allyl amino acids required in HBS strategy

Supplementary Table 10. Linear peptides in this work: label, sequence and % helicity in PBS buffer.

label		sequence	% helicity
controls	AAKA	Ac-**AAAAKA**AAAKAAAAKAW	48
	ADAA	Ac-**ADAAAA**AAAKAAAAKAW	55
variations of N3 (N',N4) as (L,L) 17-mers	LDQL	Ac-**LDAAQL**AAAKAAAAKAW	37
	LDEL	Ac-**LDAAEL**AAAKAAAAKAW	52
	LDTL	Ac-**LDAATL**AAAKAAAAKAW	63
	LDSL	Ac-**LDAASL**AAAKAAAAKAW	67
	LDAL	Ac-**LDAAAL**AAAKAAAAKAW	69
	LDFL	Ac-**LDAAFL**AAAKAAAAKAW	69
	LDLL	Ac-**LDAALL**AAAKAAAAKAW	93
variations of (N',N4) N3 as L 17-mers	**VDLL**	Ac-**VDAALL**AAAKAAAAKAW	48
	**LDLI**	Ac-**LDAALI**AAAKAAAAKAW	52
	**FDLL**	Ac-**FDAALL**AAAKAAAAKAW	53
	**IDLL**	Ac-**IDAALL**AAAKAAAAKAW	69
	**LDLV**	Ac-**LDAALV**AAAKAAAAKAW	70
	**LDLF**	Ac-**LDAALF**AAAKAAAAKAW	83
	**LDLL**	Ac-**LDAALL**AAAKAAAAKAW	93
Ala scan in LDLL 17-mers	LDLA	Ac-**LDAALA**AAAKAAAAKAW	39
	ADLL	Ac-**ADAALL**AAAKAAAAKAW	45
	LALL	Ac-**LAAALL**AAAKAAAAKAW	45
	LDAL	Ac-**LDAAAL**AAAKAAAAKAW	69
	LDLL	Ac-**LDAALL**AAAKAAAAKAW	93
length variations 12,17,22-mers	AAKA **12-mer**	Ac-**AAAAKA**AAAKAW	17
	AAKA **17-mer**	Ac-**AAAAKA**AAAKAAAAKAW	48
	AAKA **22-mer**	Ac- **AAAAKA**AAAKAAAAKAAAAKAW	64
	LDLL **12-mer**	Ac-**LDAALL**AAAKAW	28
	LDLL **17-mer**	Ac-**LDAALL**AAAKAAAAKAW	93
	LDLL **22-mer**	Ac-**LDAALL**AAAKAAAAKAAAAKAW	101

Supplementary Table 11. Percent helicity and ellipticity ratios 222/208 nm of controls and bicyclo 12-mers in PBS and in 10% TFE/PBS.

label	peptide sequence	solvent	% helicity	$\theta_{222}/\theta_{208}$
AAKA 12-mer	Ac-AAAAKAAAAKAW	PBS	17	0.26
		10% TFE/PBS	35	0.47
LDLL 12-mer	Ac-LDAALLAAAKAW	PBS	28	0.39
		10% TFE/PBS	43	0.57
{*N' – N3*} 12-mer	W{CDAAC}AAAAKA	PBS	32	0.36
		10% TFE/PBS	45	0.41
{*N' – N4*} 12-mer	W{CDAAAC}AAAKA	PBS	34	0.37
		10% TFE/PBS	47	0.49
bicyclo 12-mer	W{CDAACC}AAAKA	PBS	59	0.76
		10% TFE/PBS	72	1.21

Supplementary Table 12. Percent helicity, ellipticity ratio 222/208 nm in PBS and 10% TFE/PBS, and half-life in 25% human serum of wild-type and modified biological sequences.

label	sequence[1]	solvent	q_{222}/q_{208}[2]	%helicity[2]	half-life (h)
1**l**	Ac-QVARQLAEIY	PBS	0.48	16	<1
		10% TFE/PBS	0.64	20	
1**B**	{CDQVCC}$_{cyc}$QLAEIY	PBS	0.48	17	5.1
		10% TFE/PBS	0.86	43	
1**m**	C*DQVC*C*QLAEIY	PBS	\	\	<1
		10% TFE/PBS	\	\	
2**l**	Ac-QAEELLRALDQY	PBS	0.27	13	1.3
		10% TFE/PBS	0.53	23	
2**B**	{CDQACC}$_{cyc}$LLRALDQY	PBS	0.91	25	2.9
		10% TFE/PBS	1	42	
2**m**	C*DQAC*C*LLRALDQY	PBS	0.23	10	<1
		10% TFE/PBS	0.44	24	
3**l**	Ac-ASTAIREISLLKEY	PBS	0.18	11	1.3
		10% TFE/PBS	0.34	20	
3**B**	{CDASCC}$_{cyc}$IREISLLKEY	PBS	0.5	17	3.5
		10% TFE/PBS	0.84	34	
3**m**	C*DAAC*C*IREISLLKEY	PBS	0.3	12	<1
		10% TFE/PBS	\	\	
4**l**	Ac-KNIASLLRVQAHIRKKMVY	PBS	0.27	14	<1
		10% TFE/PBS	0.69	35	
4**B**	{CDAACC}$_{cyc}$KNIASLLRVQAHIRKKMVY	PBS	0.64	22	1.1
		10% TFE/PBS	1	45	
4**m**	C*DAAC*C*KNIASLLRVQAHIRKKMVY	PBS	0.29	11	<1
		10% TFE/PBS	0.73	35	
5**l**	STIEEQAKTFLDKFNHEAEDLFYQSSL	PBS	0.26	6	1.4
		10% TFE/PBS	0.49	12	
5**B**	{CDAACC}$_{cyc}$QAKTFLDKFNHEAEDLFYQSSL	PBS	0.36	10	1.6
		10% TFE/PBS	0.8	20	
5**m**	C*DAAC*C*QAKTFLDKFNHEAEDLFYQSSL	PBS	0.22	7	<1
		10% TFE/PBS	0.49	12	

[1]Red residues in linear sequences for residues which may be mutated to form BAMM *N*-cap; C* for Cys(Me)

[2]Helical characteristics of linear peptides (**1m**, **3m**) with CD spectra similar to β structures in 10% TFE/PBS are not calculated.

Supplementary Table 13. Statistics of NMR-derived structure LDLL 12-mer.

	Peptide
**NMR distance and dihedral constraints**	
Distance constraints	
Total NOE	91
Intra-residue	43
Inter-residue	
Sequential ($\|i - j\| = 1$)	28
Medium-range ($\|i - j\| < 4$)	18
Long-range ($\|i - j\| > 5$)	2
Intermolecular	
Hydrogen bonds	
Total dihedral angle restraints	
ϕ	7
ψ	
**Structure statistics**	
Violations (mean and s.d.)	
Distance constraints (Å)	0.12 ± 0.49
Dihedral angle constraints (º)	2.4 ± 10.5
Max. dihedral angle violation (º)	50
Max. distance constraint violation (Å)	2.7
Average pairwise r.m.s. deviation from 23 conformers (Å)	
Heavy	0.89
Backbone	0.21

Supplementary Table 14. Statistics of NMR-derived structure bicyclo 12-mer.

	Peptide
**NMR distance and dihedral constraints**	
Distance constraints	
Total NOE	116
Intra-residue	48
Inter-residue*	
Sequential (|*i* – *j*| = 1)	24
Medium-range (|*i* – *j*| < 4)	13
Long-range (|*i* – *j*| > 5)	
Intermolecular	
Hydrogen bonds	
Total dihedral angle restraints	
ϕ	8
ψ	
**Structure statistics**	
Violations (mean and s.d.)	
Distance constraints (Å)	0.027 ± 0.43
Dihedral angle constraints (º)	0.24 ± 0.99
Max. dihedral angle violation (º)	7.1
Max. distance constraint violation (Å)	1.43
Average pairwise r.m.s. deviation from 50 conformers (Å)	
Heavy	2.16
Backbone	0.41

*there are another 31 pairs of inter-residue restraints between hydrogens of TBMB and hydrogens of nearby amino acid residues.

AAKA/AAKA 17-mer retention time: 8.215 min calculated [M+H]$^+$: 1553.89 Observed [M+H]$^+$: 1553.8871

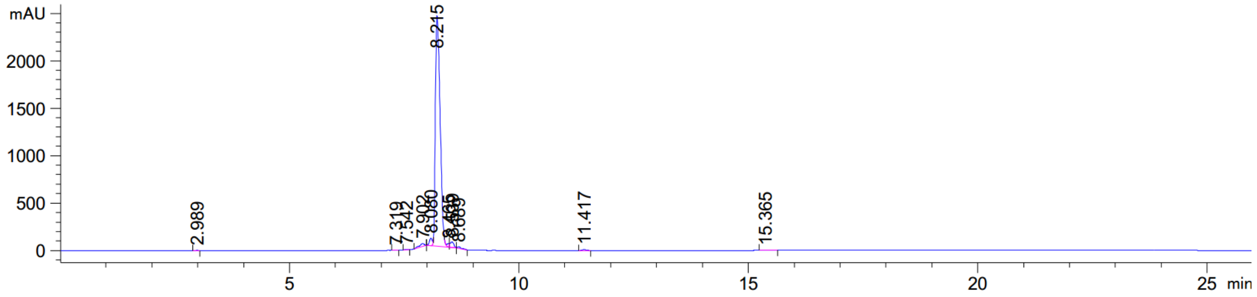

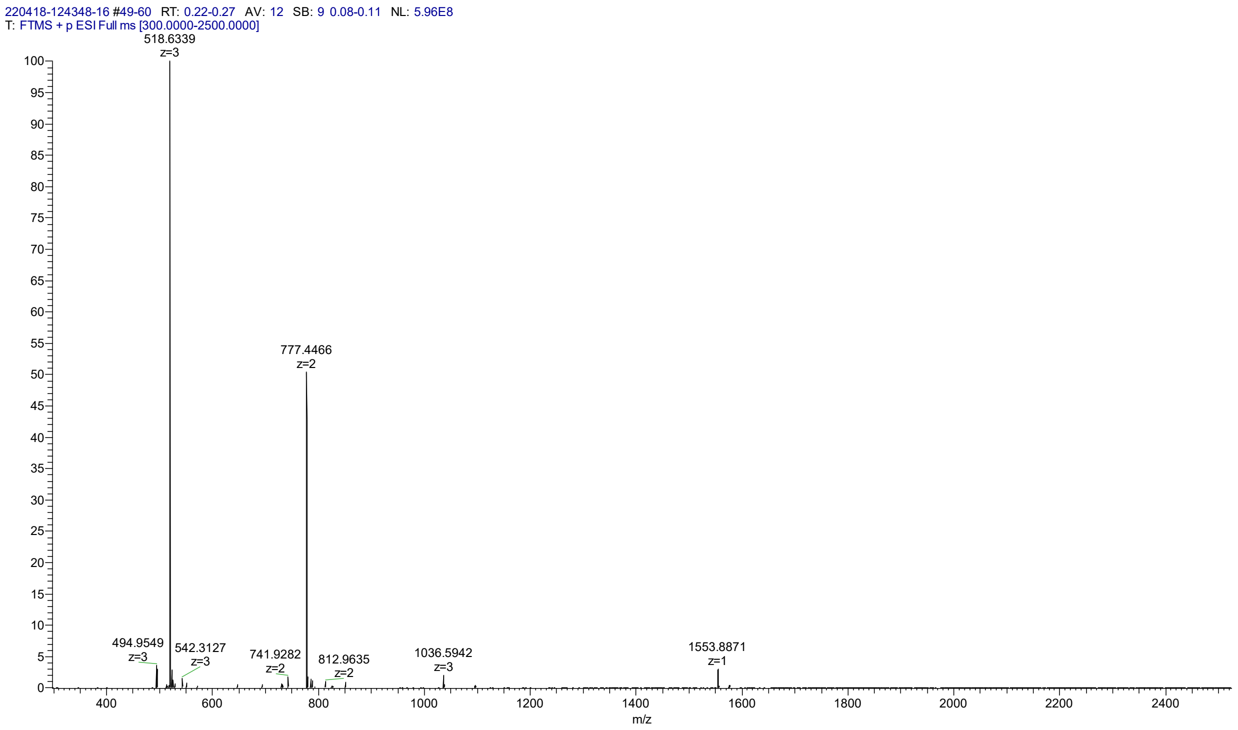

Supplementary Figure 57 Characterization of AAKA/AAKA 17-mer by analytical HPLC and high resolution MS. mAU refers to mini Arbitrary Unit.

ADAA retention time: 9.165 min calculated $[M+H]^{+}$: 1540.82 Observed $[M+H]^{+}$: 1540.8207

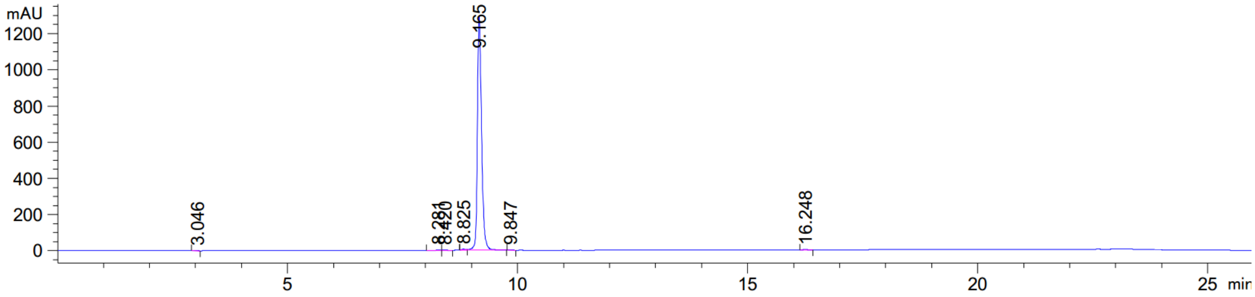

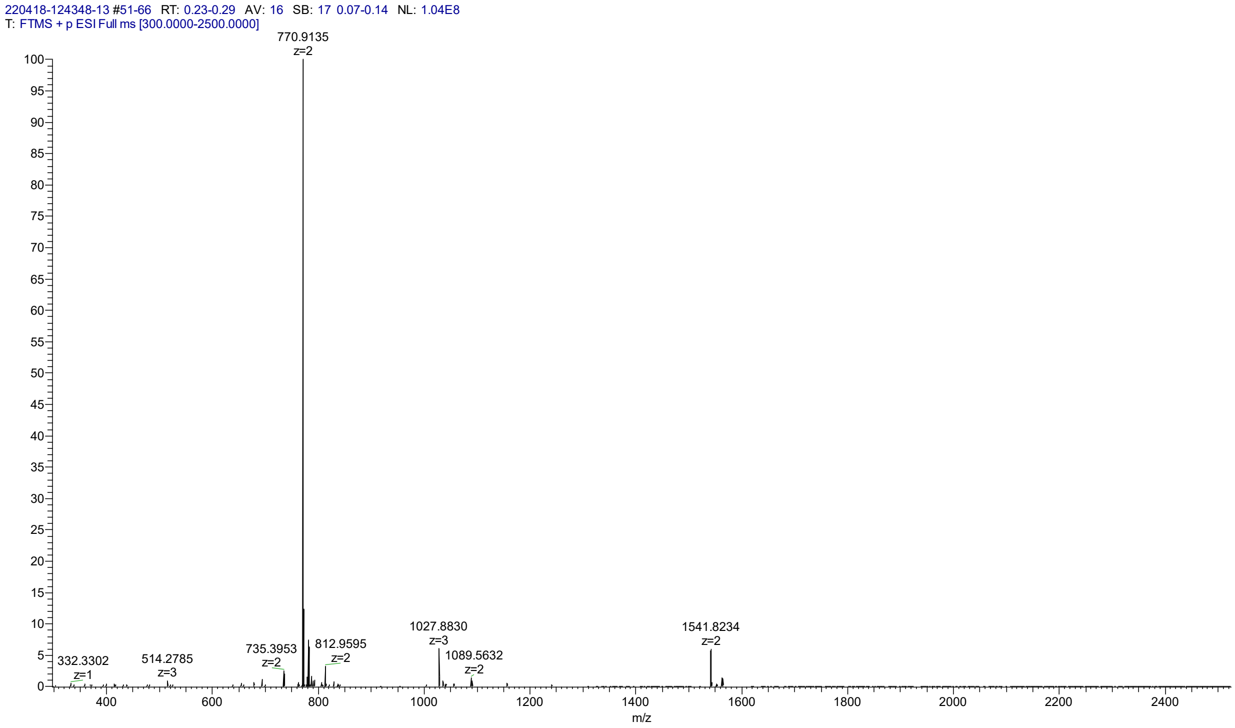

Supplementary Figure 58 Characterization of ADAA by analytical HPLC and high resolution MS. mAU refers to mini Arbitrary Unit.

LDQL retention time: 10.119 min calculated $[M+H]^+$: 1681.94 Observed $[M+H]^+$: 1681.9366

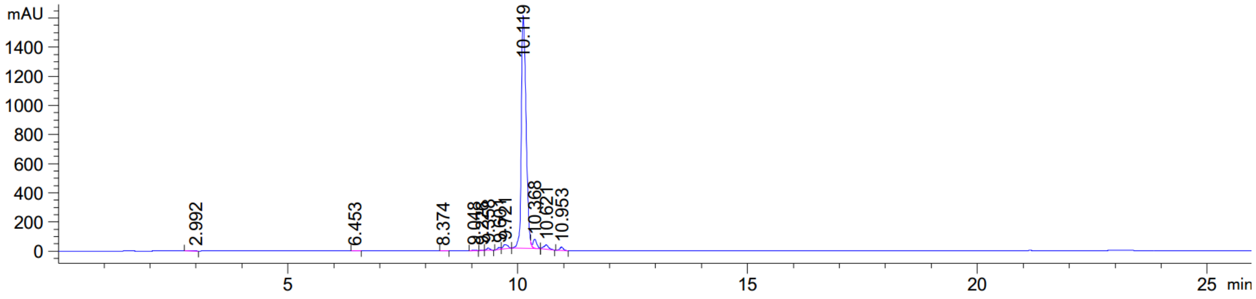

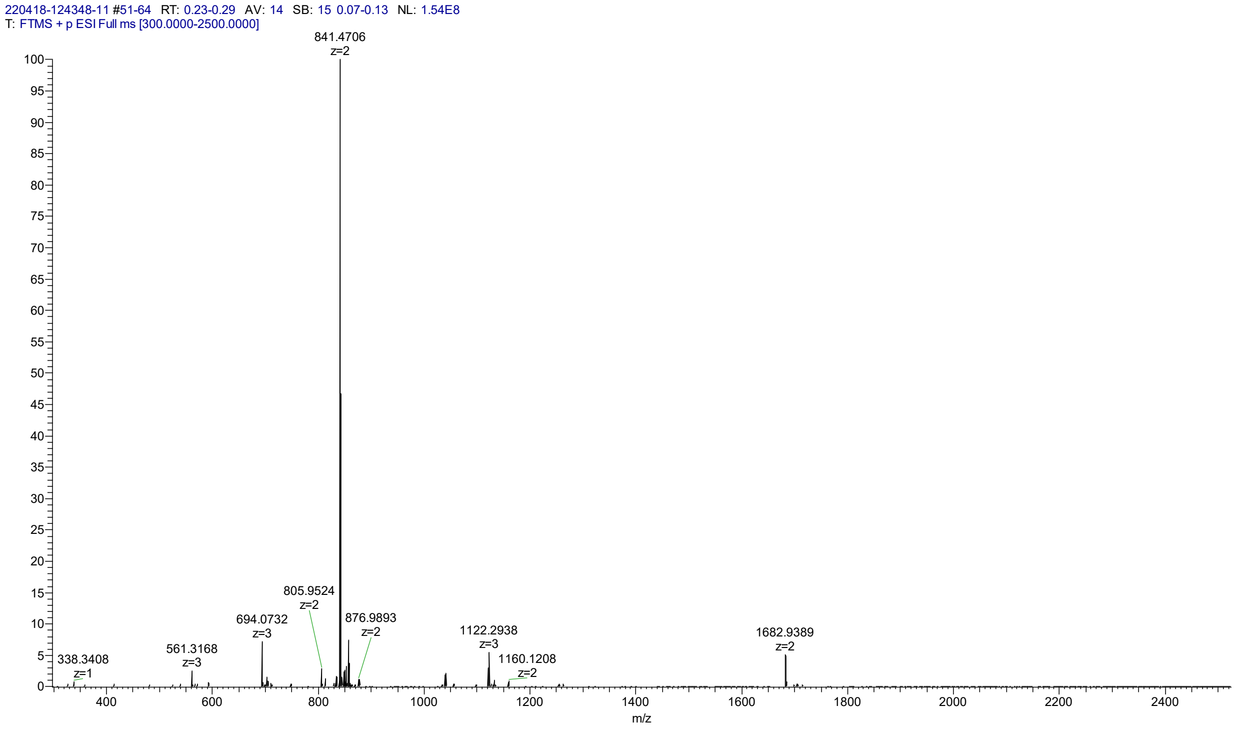

Supplementary Figure 59 Characterization of LDQL by analytical HPLC and high resolution MS. mAU refers to mini Arbitrary Unit.

LDEL retention time: 10.572 min calculated $[M+H]^+$: 1682.92 Observed $[M+H]^+$: 1682.9205

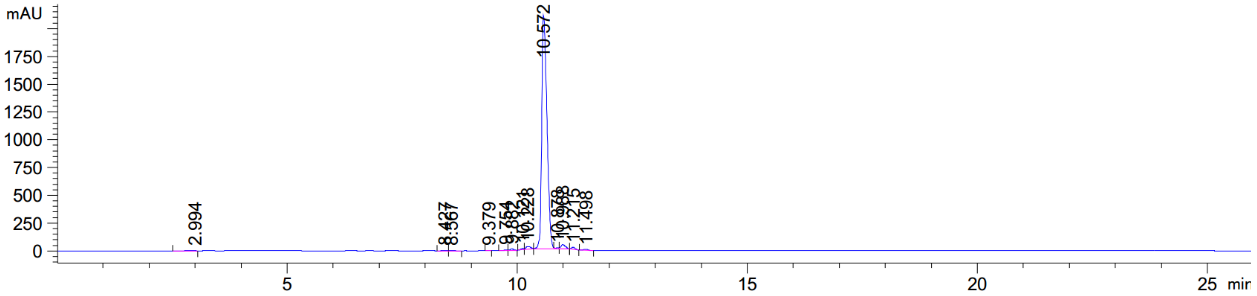

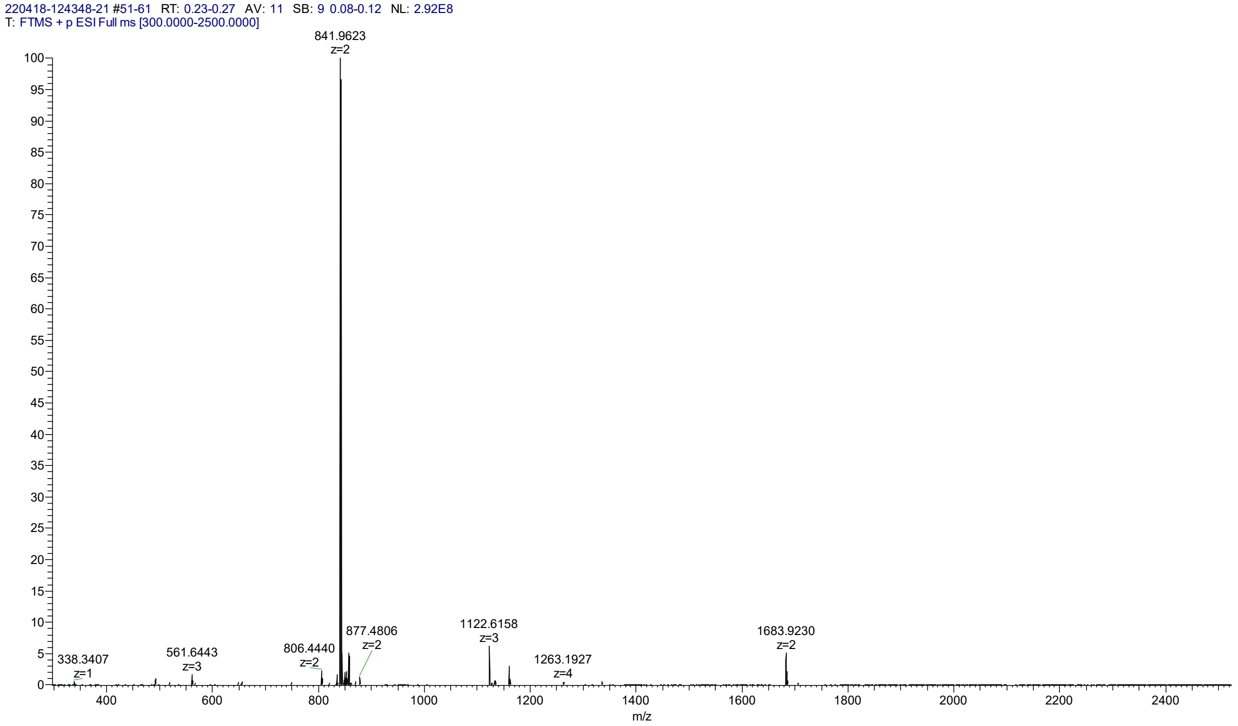

Supplementary Figure 60 Characterization of LDEL by analytical HPLC and high resolution MS. mAU refers to mini Arbitrary Unit.

LDTL retention time: 10.826 min calculated [M+H]$^+$: 1654.93 Observed [M+H]$^+$: 1654.9254

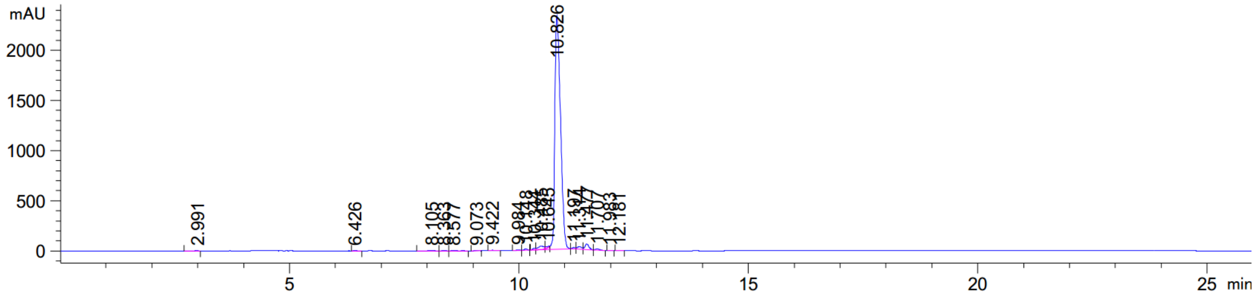

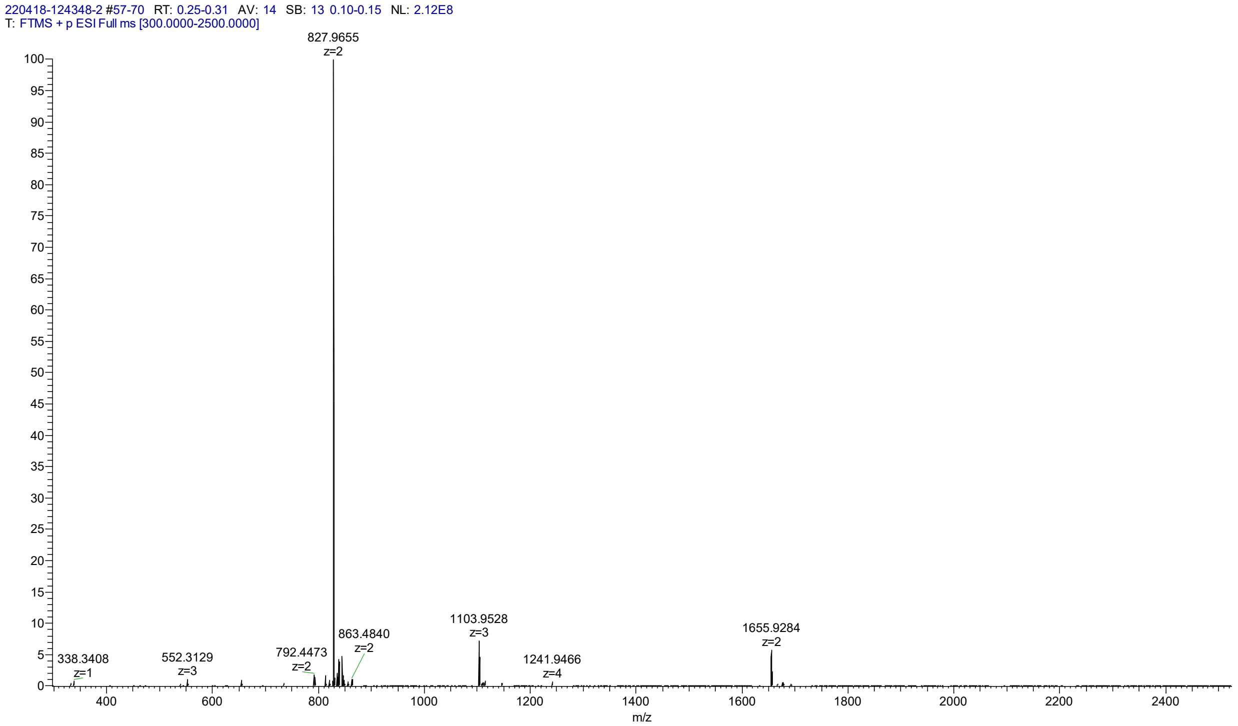

Supplementary Figure 61 Characterization of LDTL by analytical HPLC and high resolution MS. mAU refers to mini Arbitrary Unit.

LDSL retention time: 10.348 min calculated $[M+H]^+$: 1640.91 Observed $[M+H]^+$: 1640.9081

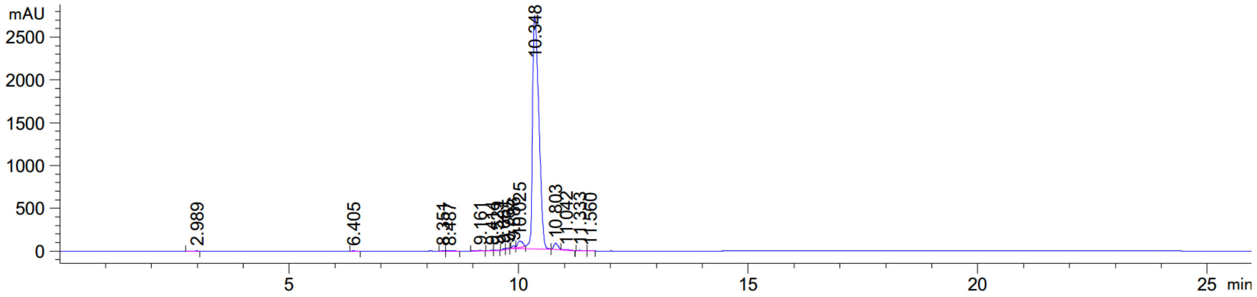

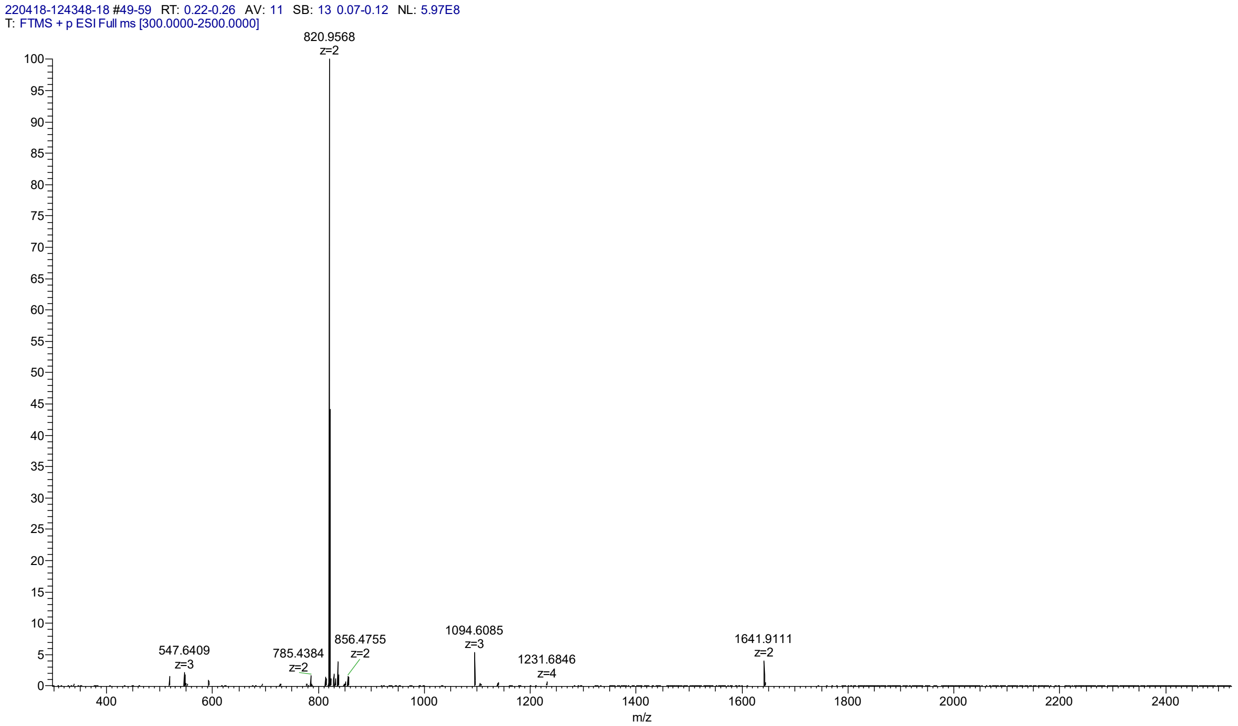

Supplementary Figure 62 Characterization of LDSL by analytical HPLC and high resolution MS. mAU refers to mini Arbitrary Unit.

LDAL retention time: 11.012 min calculated $[M+H]^+$: 1624.92 Observed $[M+H]^+$: 1624.9131

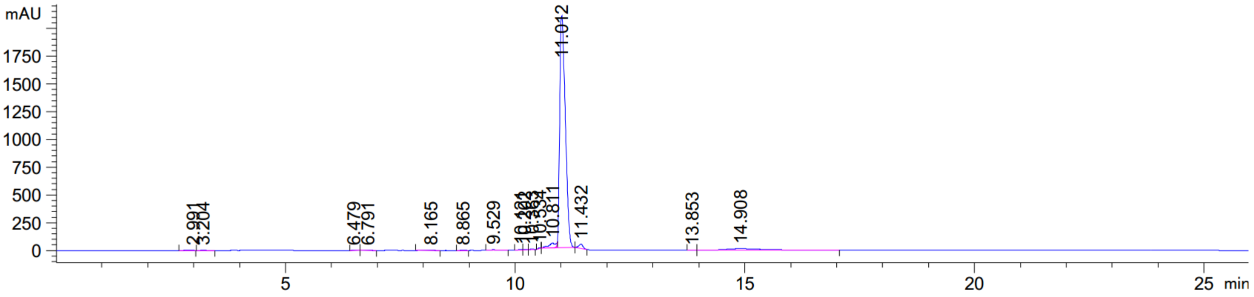

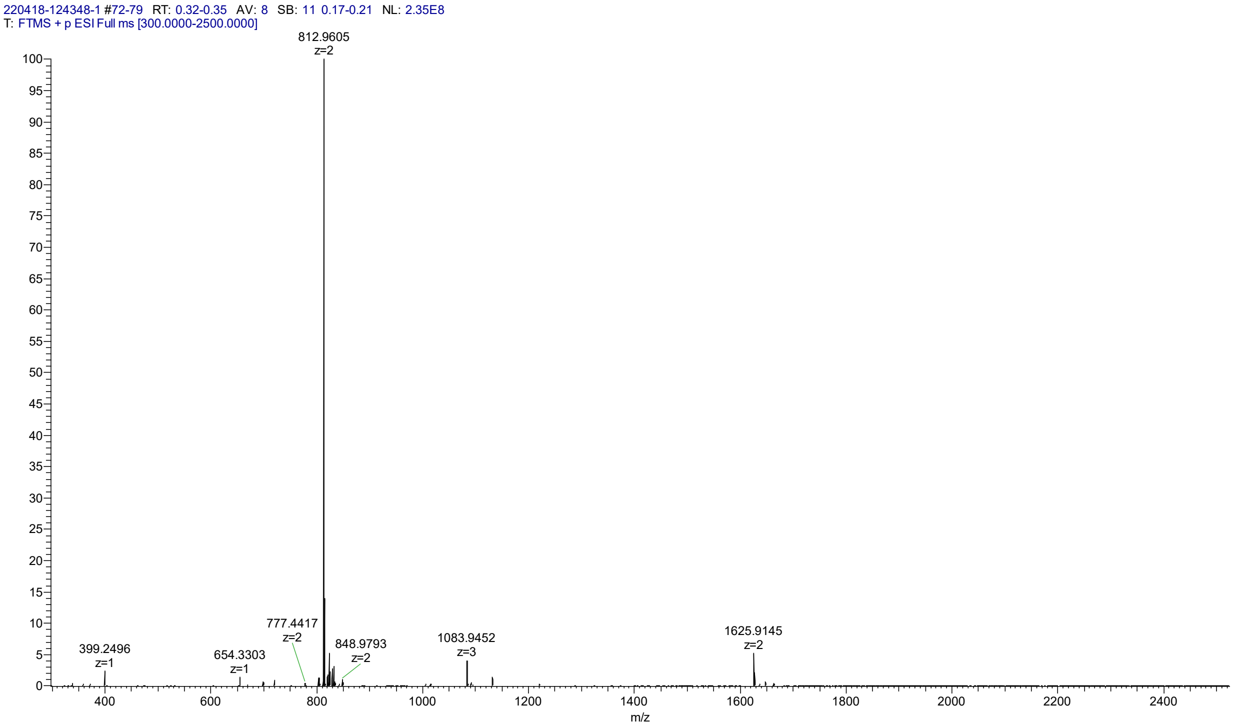

Supplementary Figure 63 Characterization of LDAL by analytical HPLC and high resolution MS. mAU refers to mini Arbitrary Unit.

LDFL retention time: 12.370 min calculated [M+H]$^+$: 1700.95 Observed [M+H]$^+$: 1700.9455

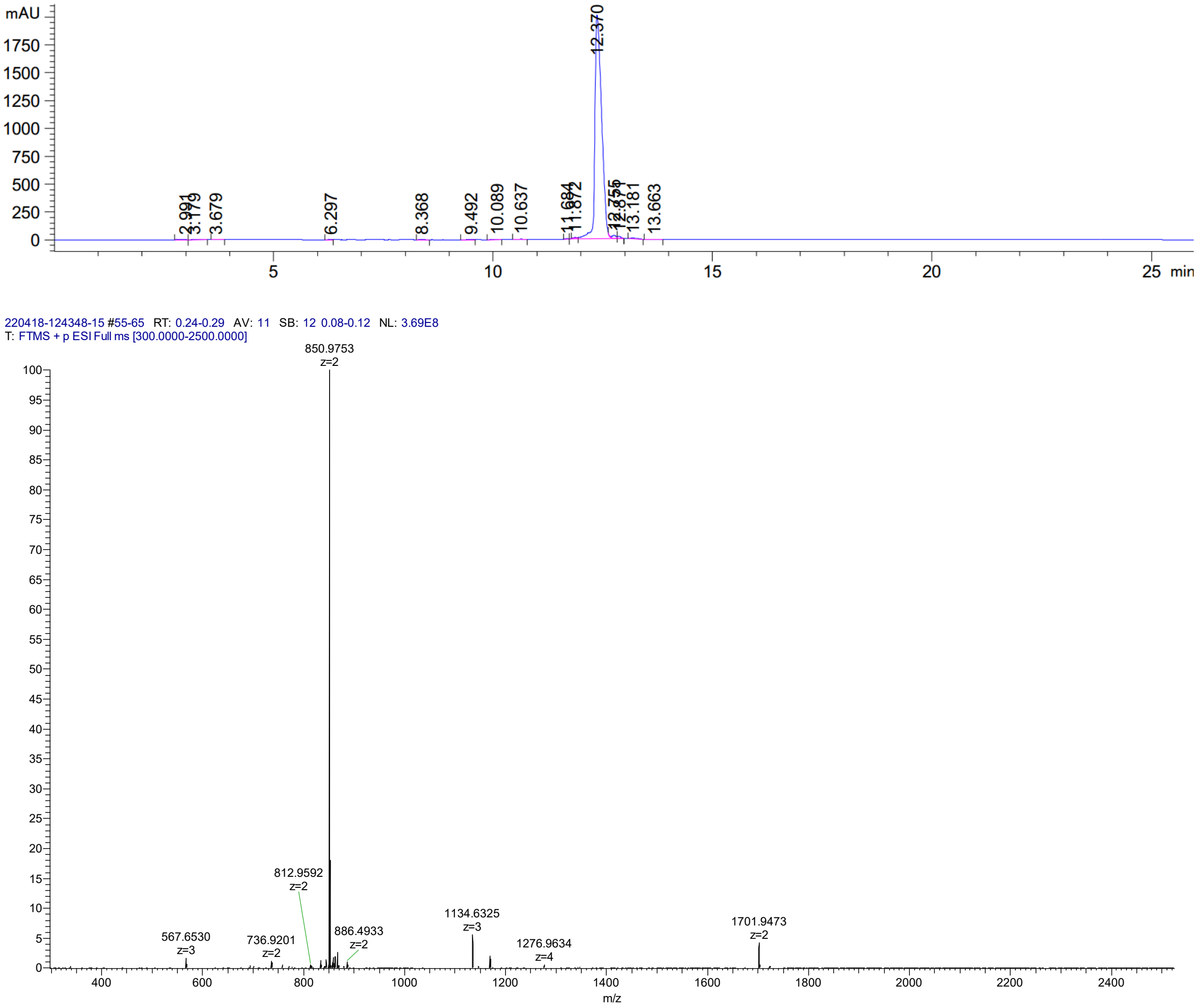

Supplementary Figure 64 Characterization of LDFL by analytical HPLC and high resolution MS. mAU refers to mini Arbitrary Unit.

LDLL/LDLL 17-mer retention time: 12.449 min calculated [M+H]$^+$: 1666.96 Observed [M+H]$^+$: 1666.9607

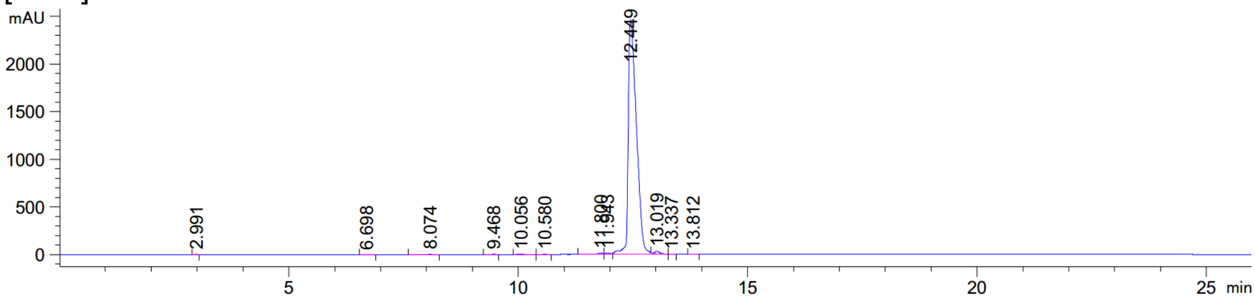

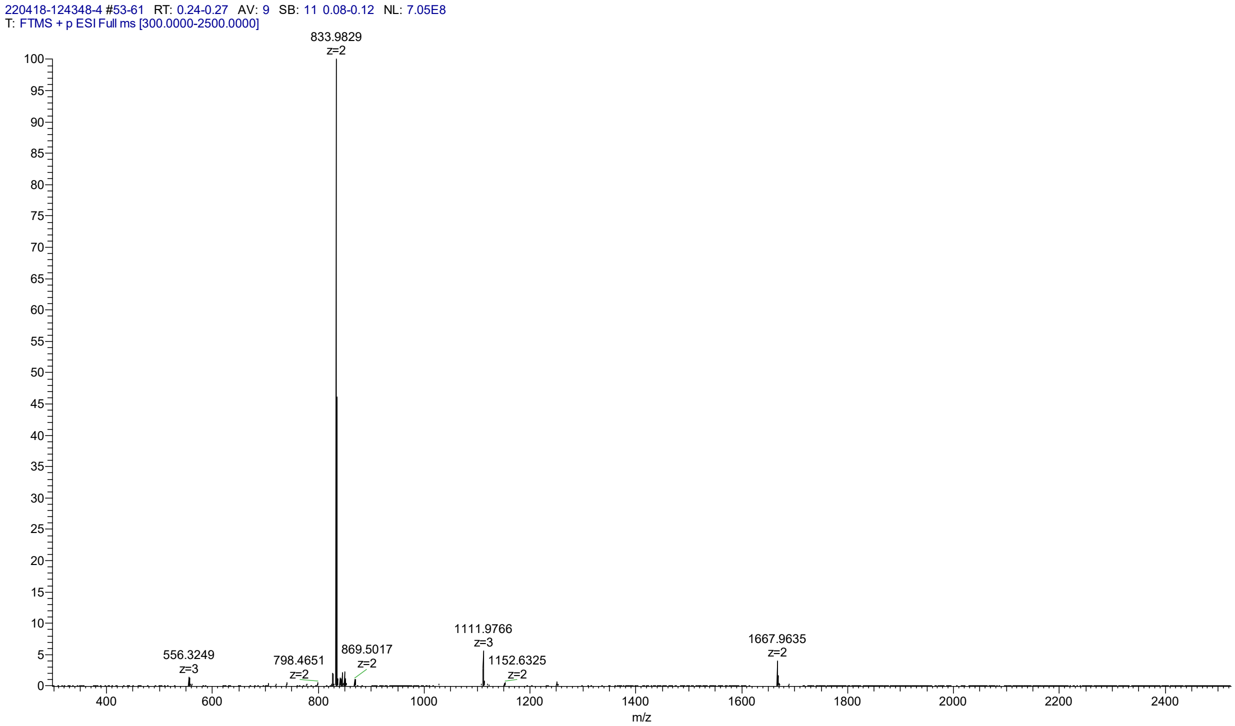

Supplementary Figure 65 Characterization of LDLL/LDLL 17-mer by analytical HPLC and high resolution MS. mAU refers to mini Arbitrary Unit.

VDLL retention time: 12.024 min calculated $[M+H]^+$: 1652.95 Observed $[M+H]^+$: 1652.9458

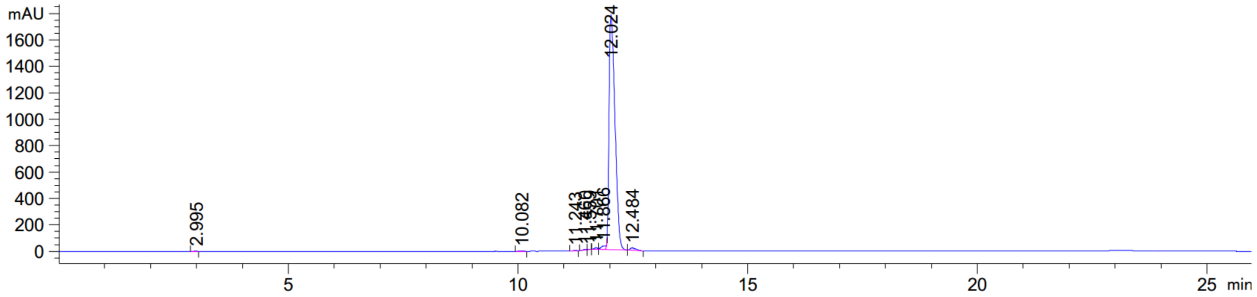

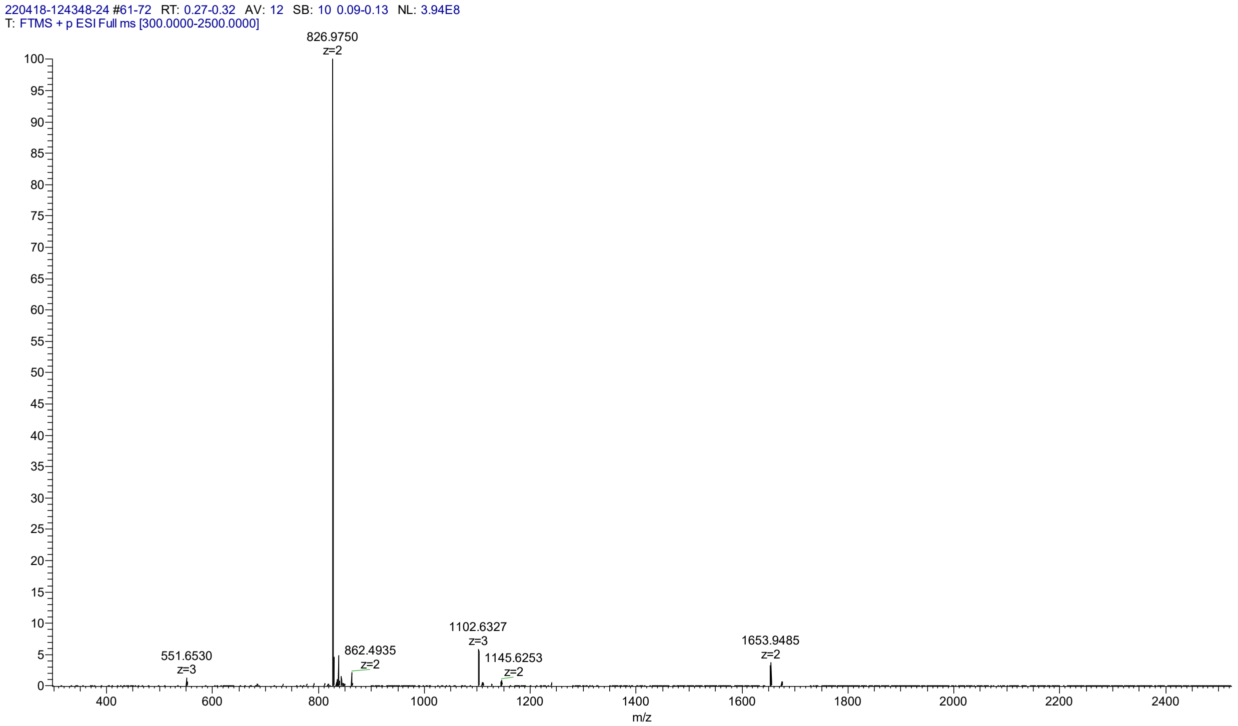

Supplementary Figure 66 Characterization of VDLL by analytical HPLC and high resolution MS. mAU refers to mini Arbitrary Unit.

LDLI retention time: 12.506 min calculated $[M+H]^+$: 1666.96 Observed $[M+H]^+$: 1666.9637

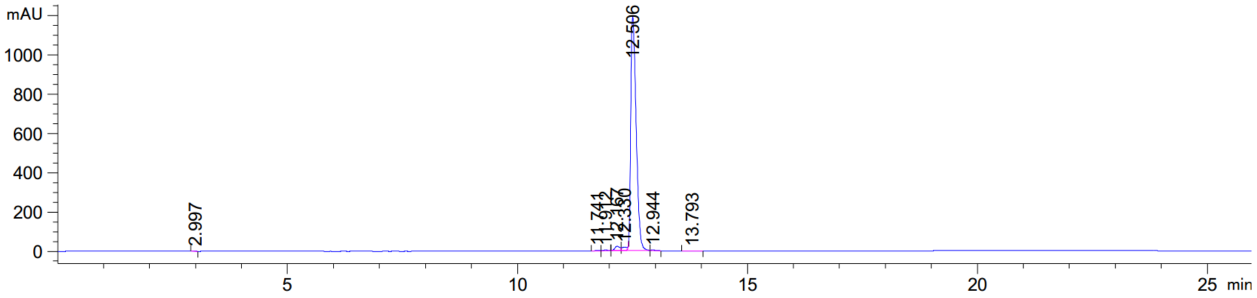

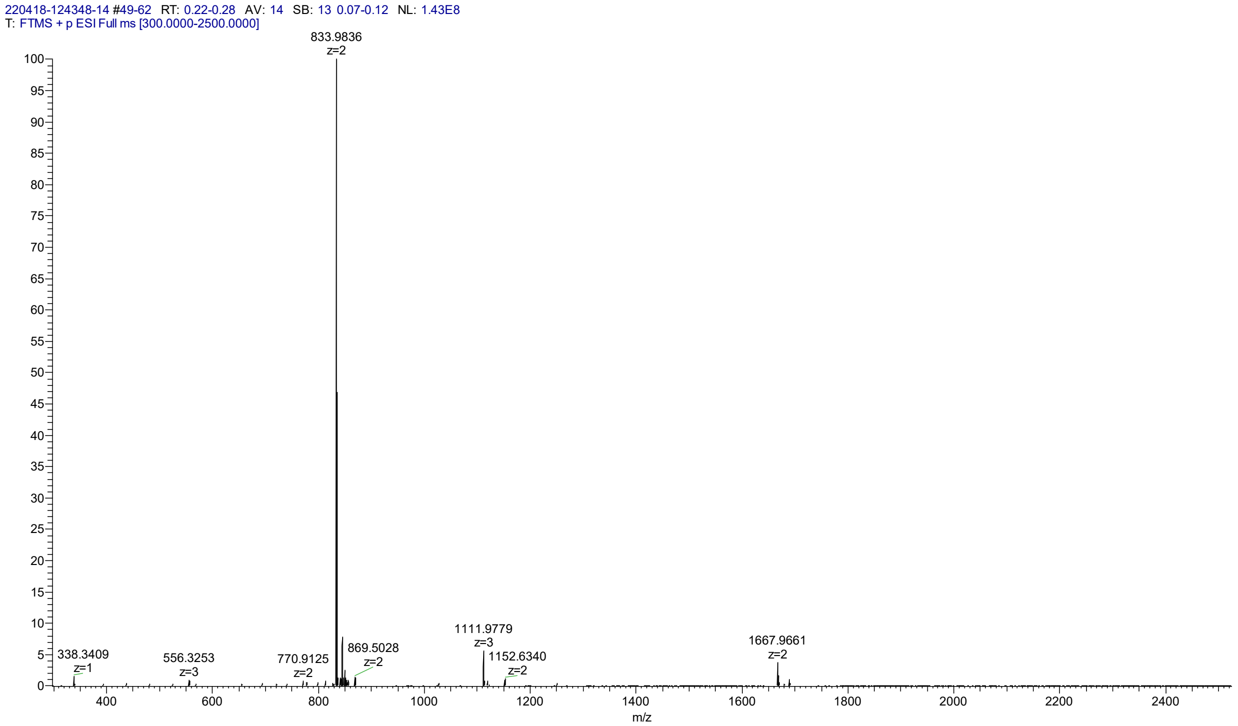

Supplementary Figure 67 Characterization of LDLI by analytical HPLC and high resolution MS. mAU refers to mini Arbitrary Unit.

FDLL retention time: 12.539 min calculated $[M+H]^+$: 1700.95 Observed $[M+H]^+$: 1700.9472

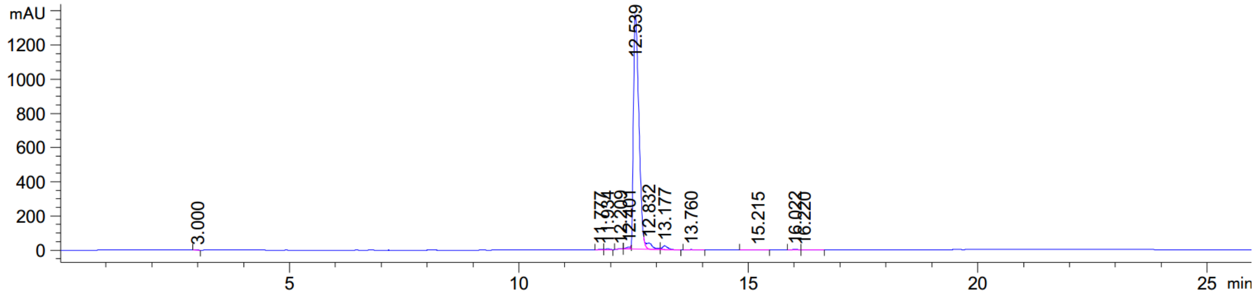

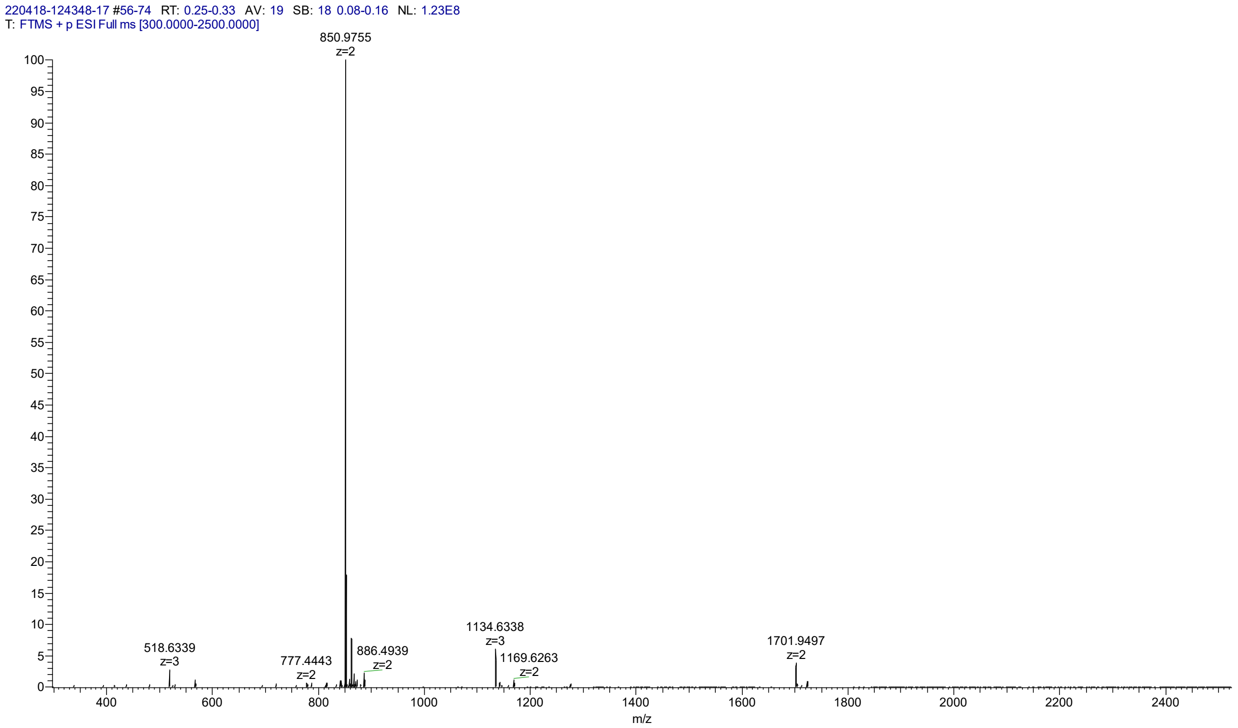

Supplementary Figure 68 Characterization of FDLL by analytical HPLC and high resolution MS. mAU refers to mini Arbitrary Unit.

IDLL retention time: 12.448 min calculated [M+H]$^+$: 1666.96 Observed [M+H]$^+$: 1666.9607

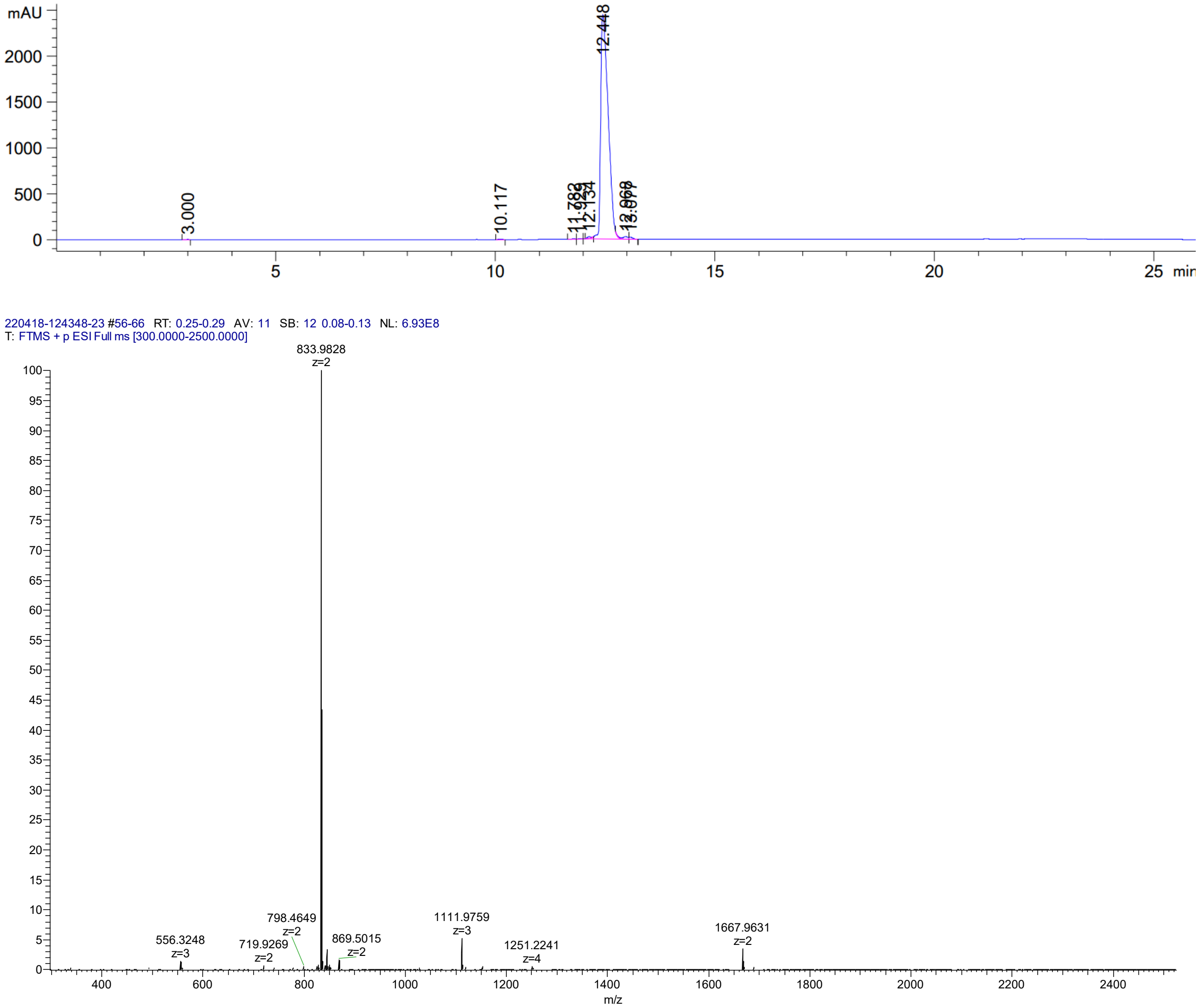

Supplementary Figure 69 Characterization of IDLL by analytical HPLC and high resolution MS. mAU refers to mini Arbitrary Unit.

LDLV retention time: 12.180 min calculated [M+H]$^+$: 1652.95 Observed [M+H]$^+$: 1652.9472

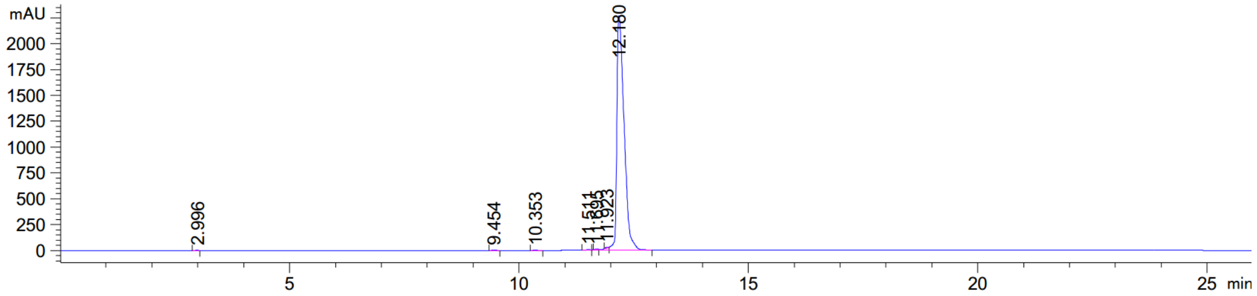

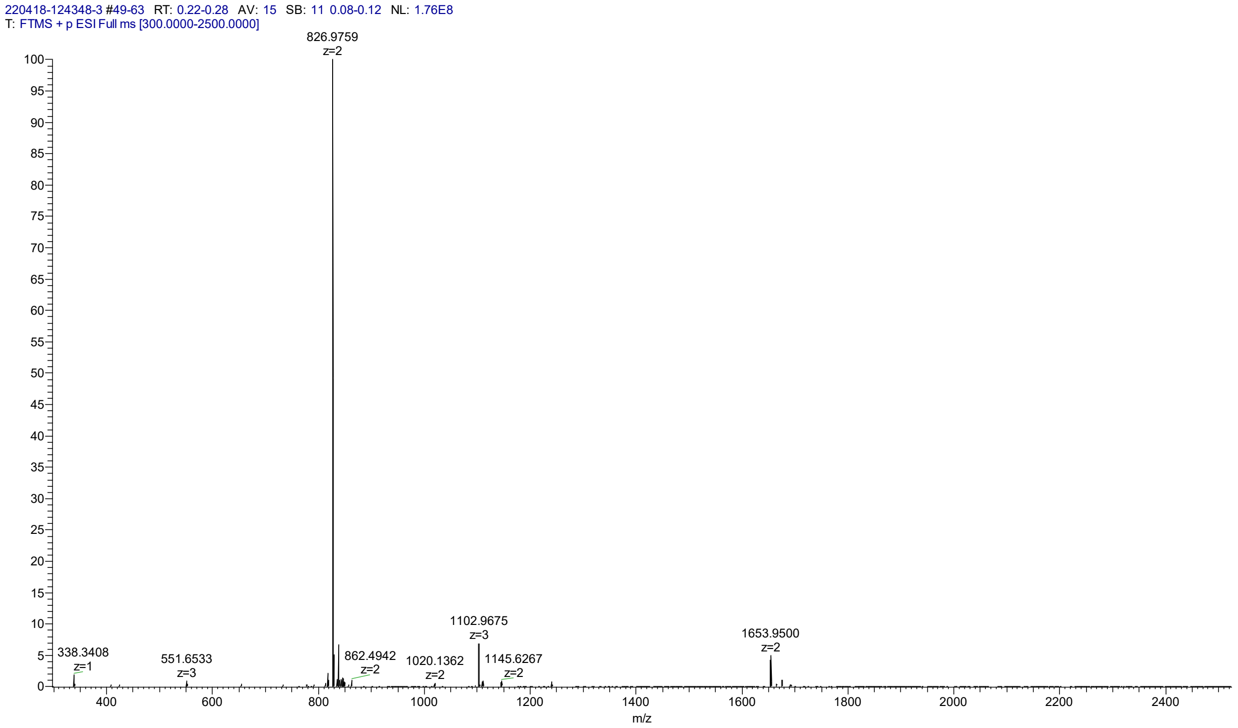

Supplementary Figure 70 Characterization of LDLV by analytical HPLC and high resolution MS. mAU refers to mini Arbitrary Unit.

LDLF retention time: 12.604 min calculated $[M+H]^+$: 1700.95 Observed $[M+H]^+$: 1700.9458

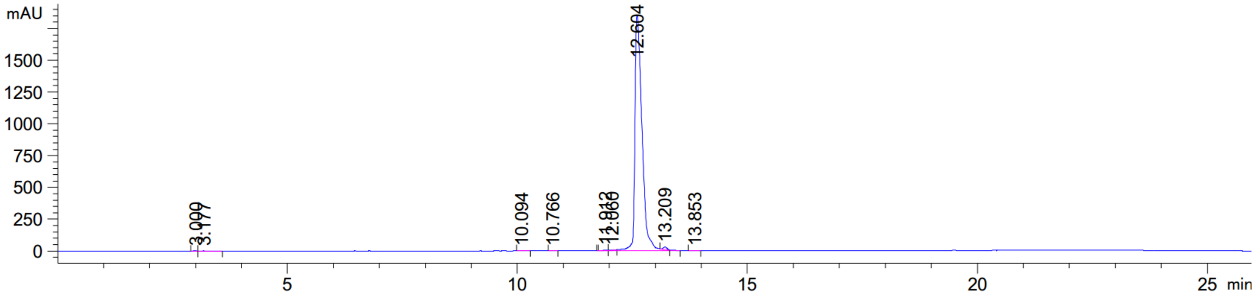

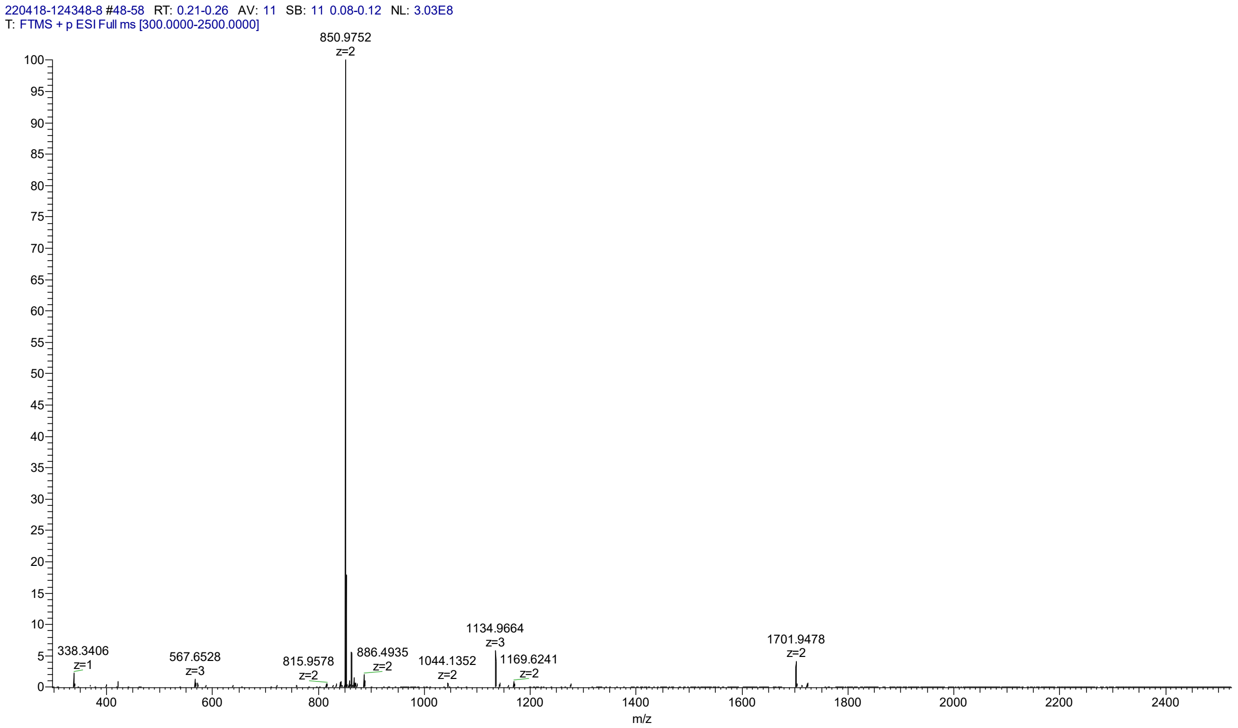

Supplementary Figure 71 Characterization of LDLF by analytical HPLC and high resolution MS. mAU refers to mini Arbitrary Unit.

LDLA retention time: 11.691 min calculated $[M+H]^+$: 1624.92 Observed $[M+H]^+$: 1624.9138

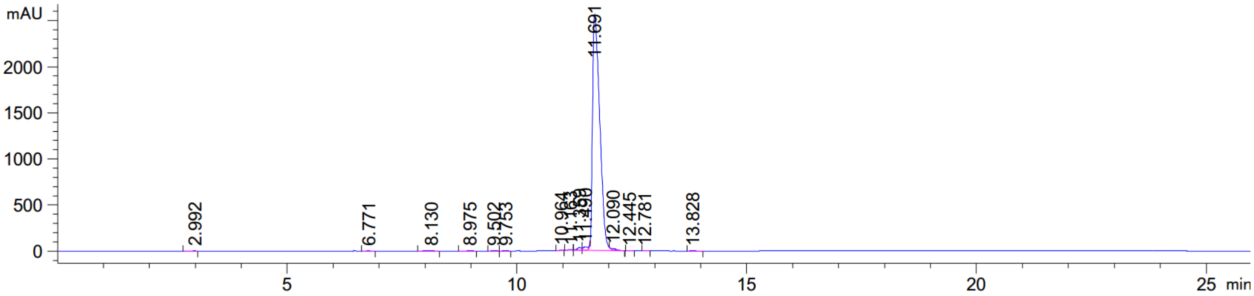

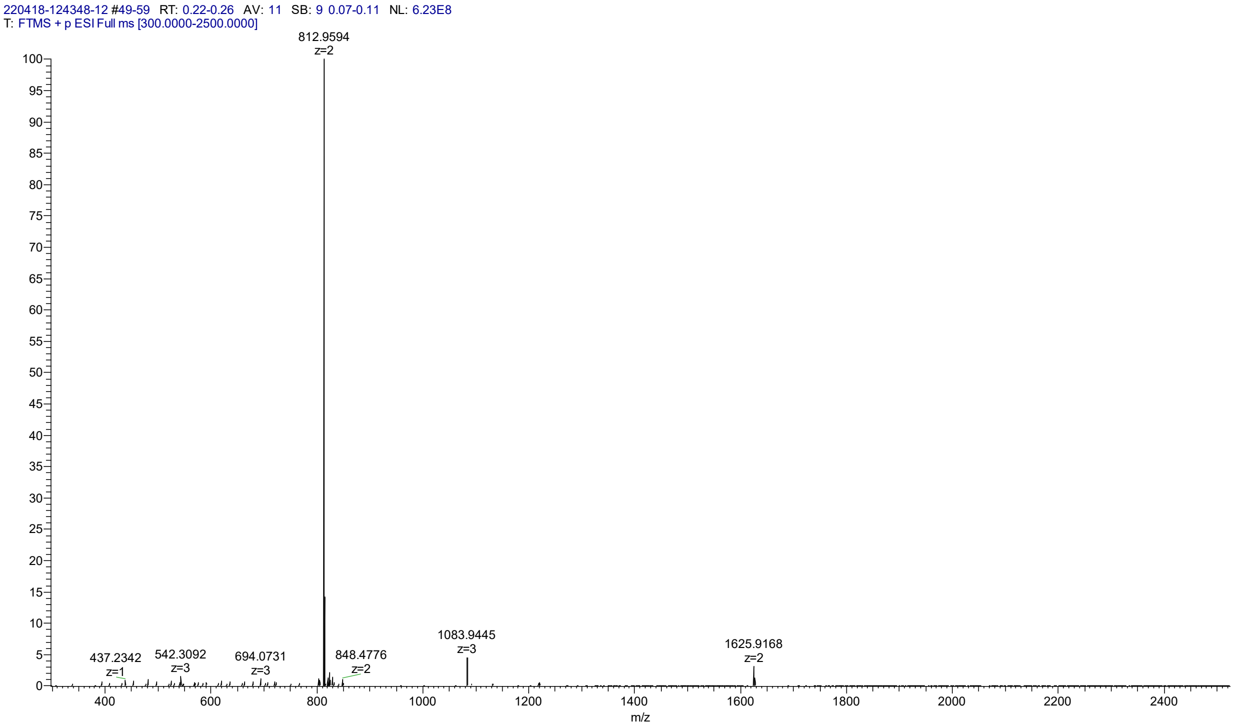

Supplementary Figure 72 Characterization of LDLA by analytical HPLC and high resolution MS. mAU refers to mini Arbitrary Unit.

ADLL retention time: 11.287 min calculated [M+H]$^+$: 1624.92 Observed [M+H]$^+$: 1624.9133

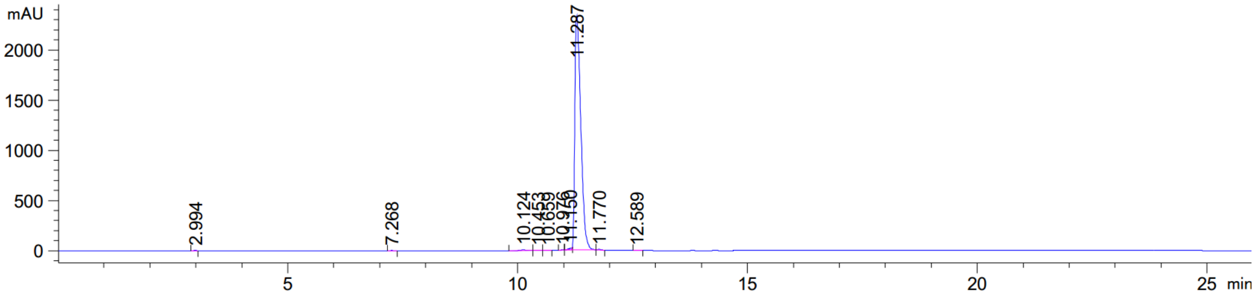

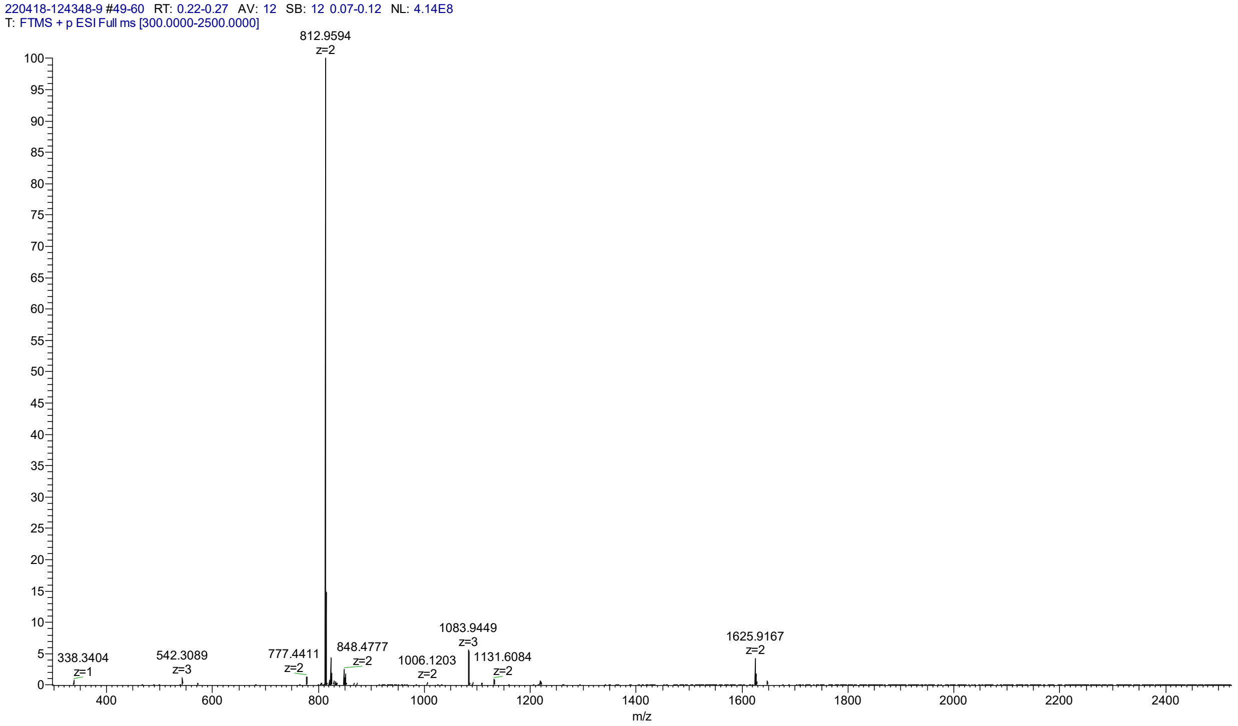

Supplementary Figure 73 Characterization of ADLL by analytical HPLC and high resolution MS. mAU refers to mini Arbitrary Unit.

LALL retention time: 14.159 min calculated $[M+H]^+$: 1622.97 Observed $[M+H]^+$: 1622.9687

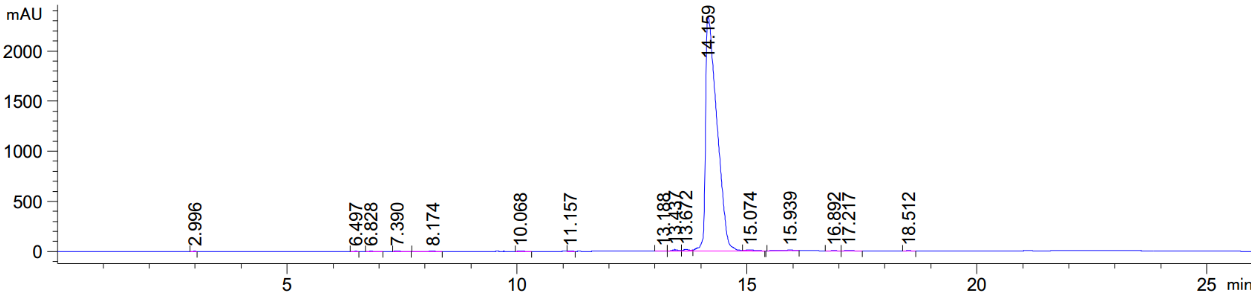

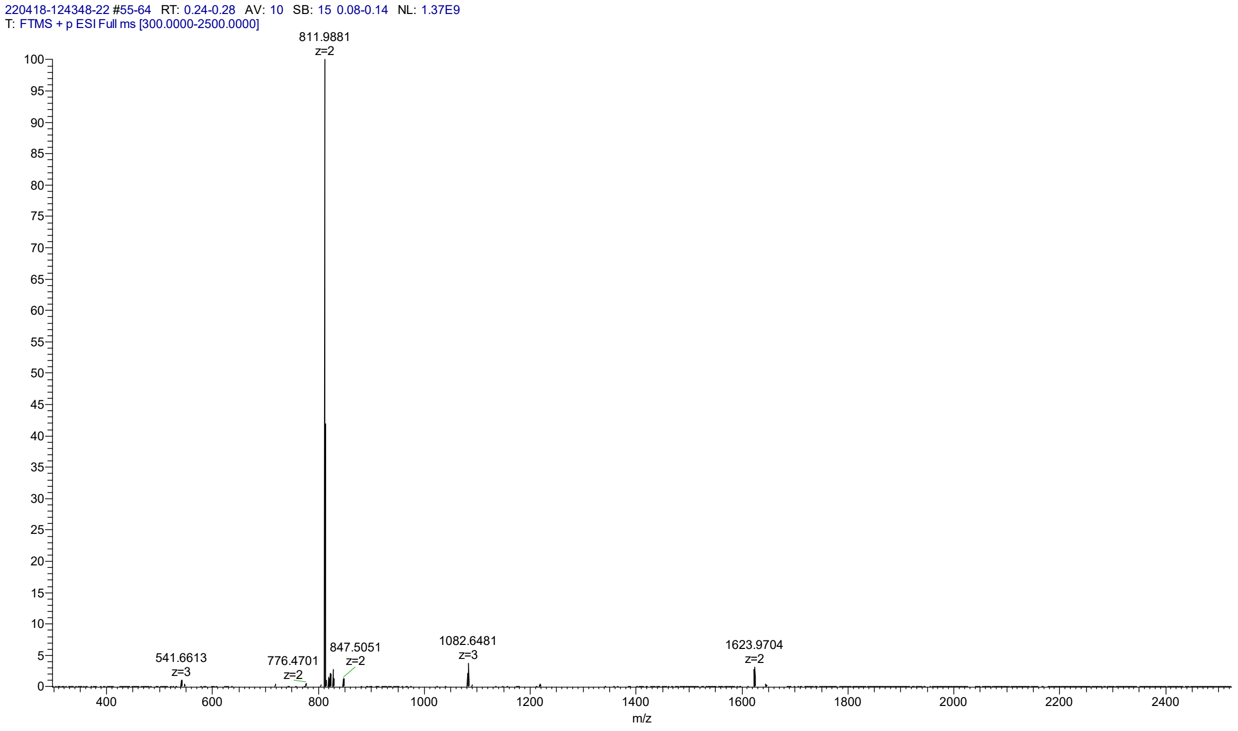

Supplementary Figure 74 Characterization of LALL by analytical HPLC and high resolution MS. mAU refers to mini Arbitrary Unit.

AAKA 12-mer retention time: 8.389 min calculated $[M+H]^+$: 1141.65 Observed $[M+H]^+$: 1141.6445

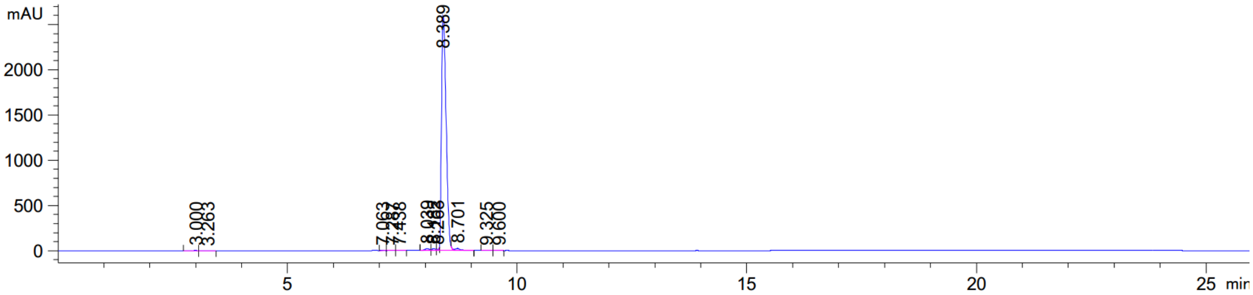

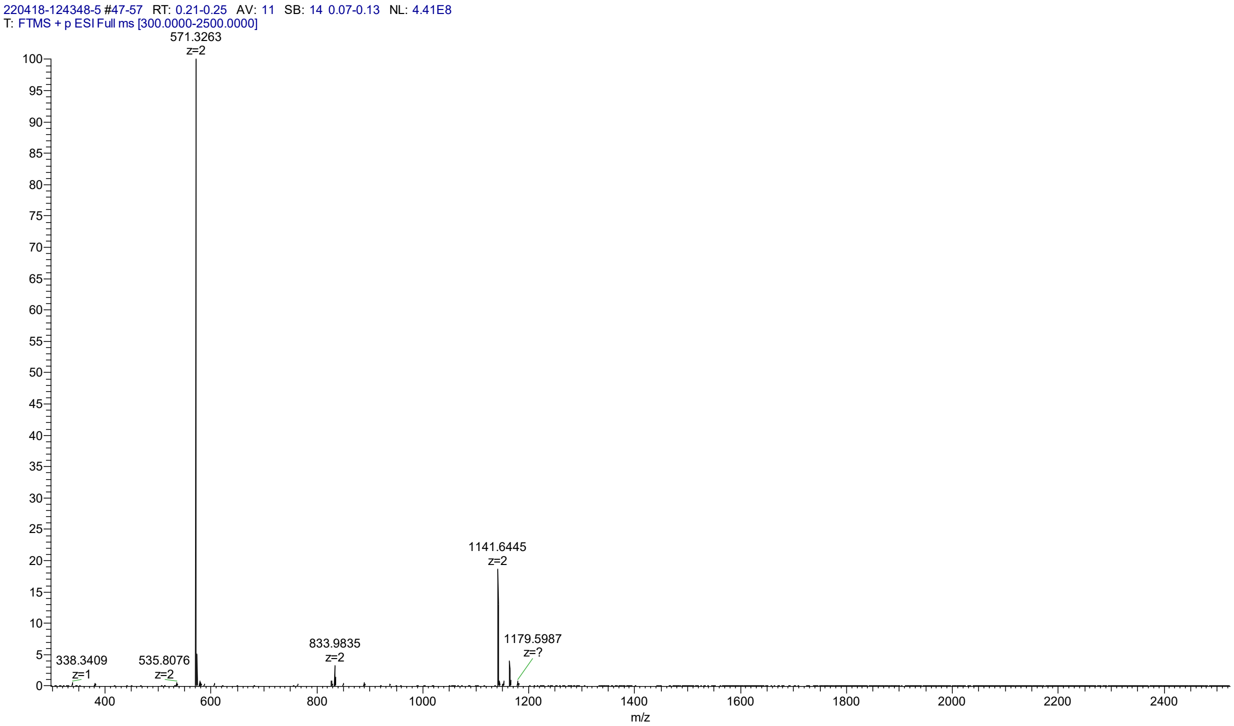

Supplementary Figure 75 Characterization of AAKA 12-mer by analytical HPLC and high resolution MS. mAU refers to mini Arbitrary Unit.

AAKA 22-mer retention time: 8.551 min calculated $[M+H]^+$: 1966.14 Observed $[M+H]^+$: 1966.1343

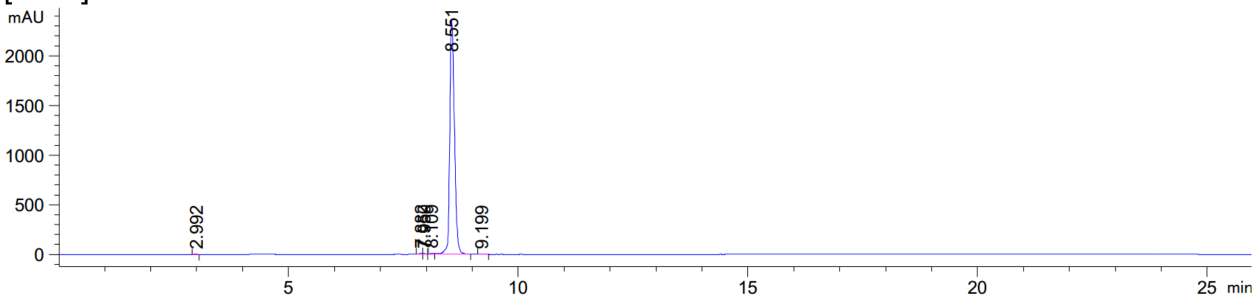

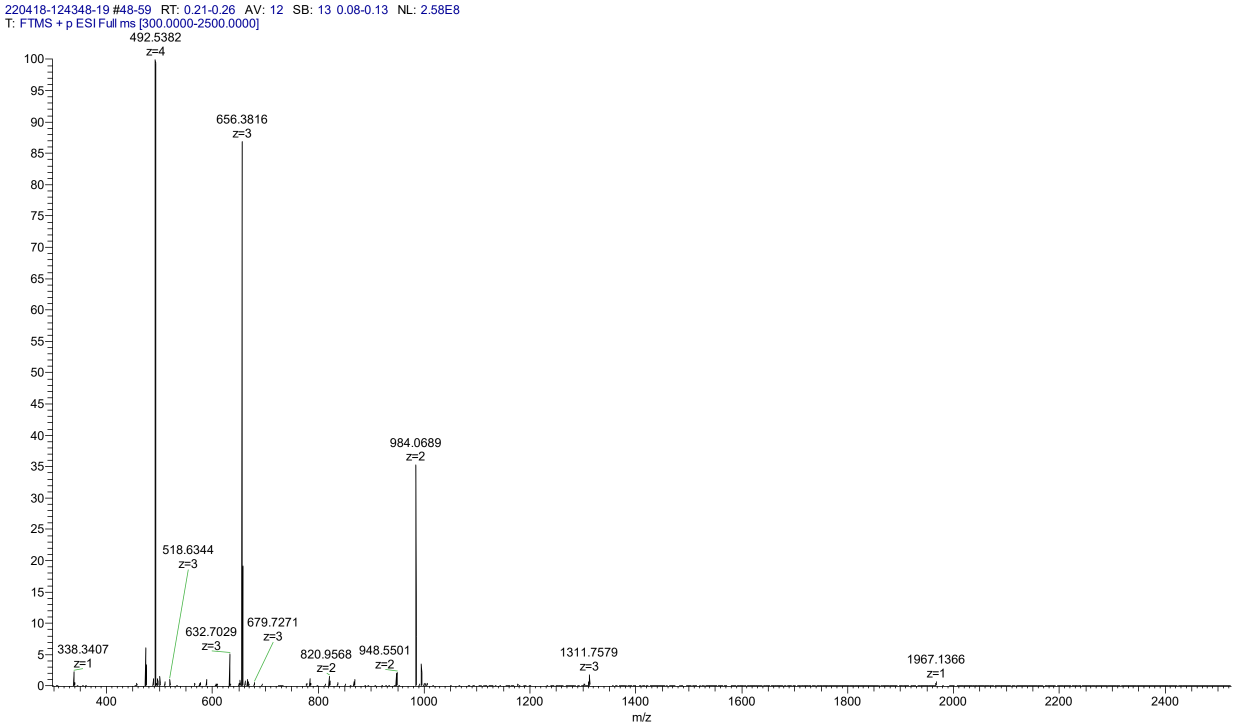

Supplementary Figure 76 Characterization of AAKA 22-mer by analytical HPLC and high resolution MS. mAU refers to mini Arbitrary Unit.

LDLL 12-mer retention time: 13.404 min calculated $[M+H]^+$: 1254.72 Observed $[M+H]^+$: 1254.7173

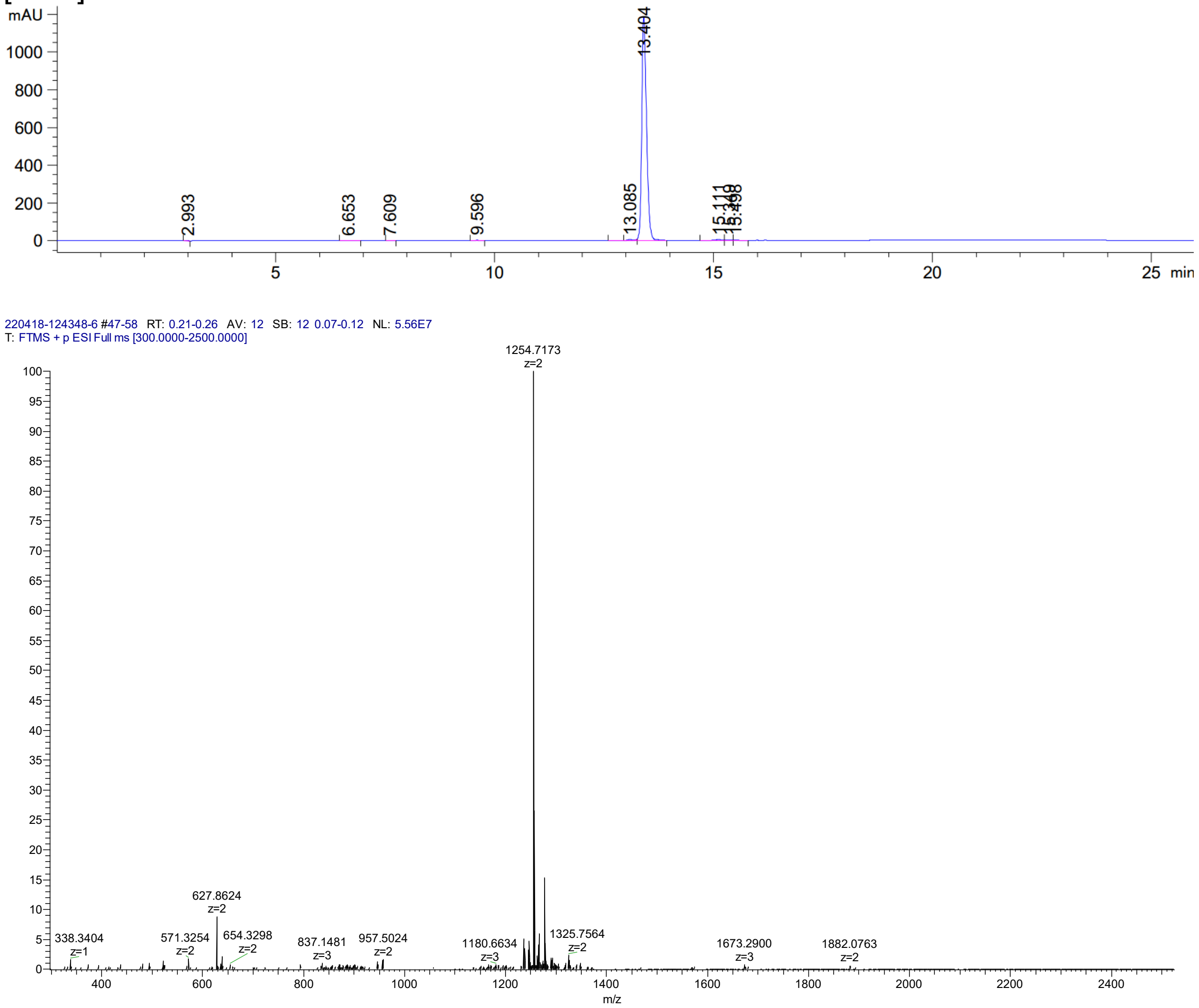

Supplementary Figure 77 Characterization of LDLL 12-mer by analytical HPLC and high resolution MS. mAU refers to mini Arbitrary Unit.

LDLL 22-mer retention time: 11.434 min calculated [M+H]$^+$: 2079.21 Observed [M+H]$^+$: 2079.2076

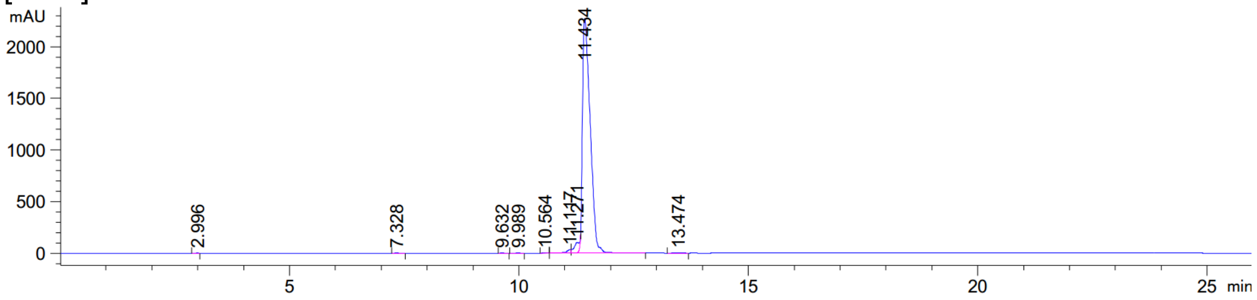

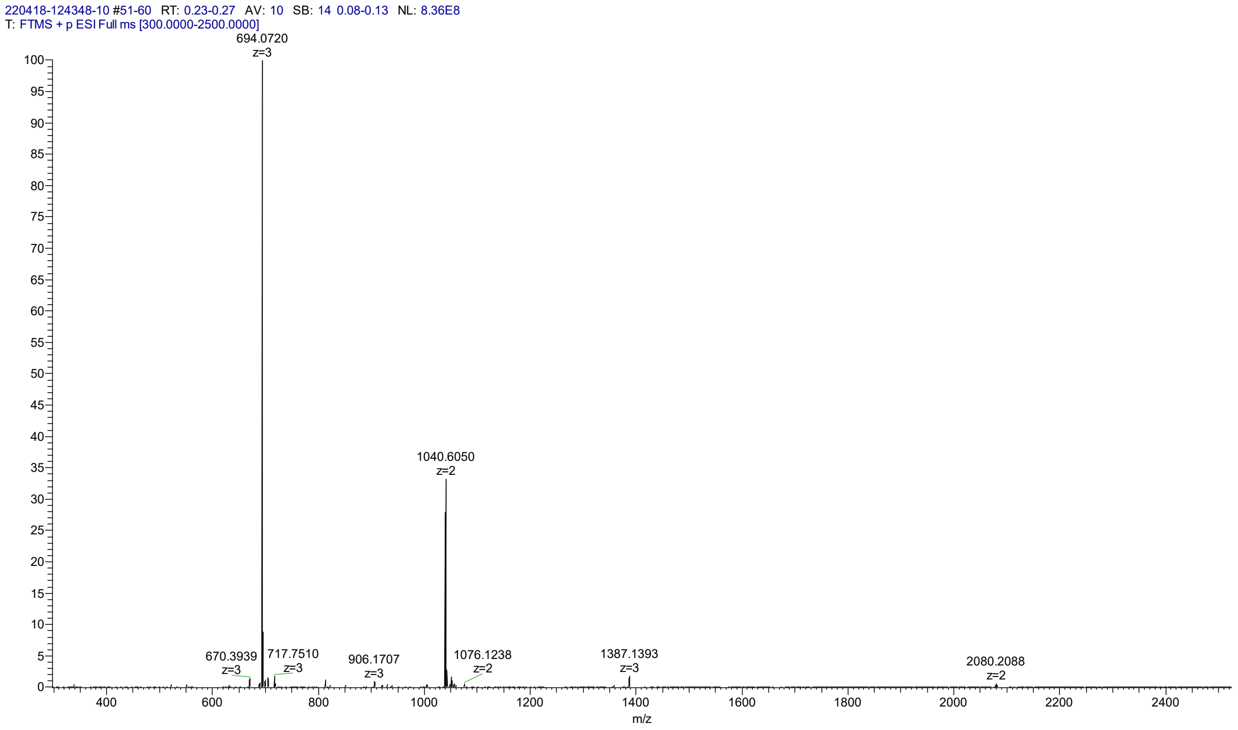

Supplementary Figure 78 Characterization of LDLL 22-mer by analytical HPLC and high resolution MS. mAU refers to mini Arbitrary Unit.

bicyclo 12-mer retention time: 9.049 min calculated $[M+H]^+$: 1296.53 Observed $[M+H]^+$: 1296.5301

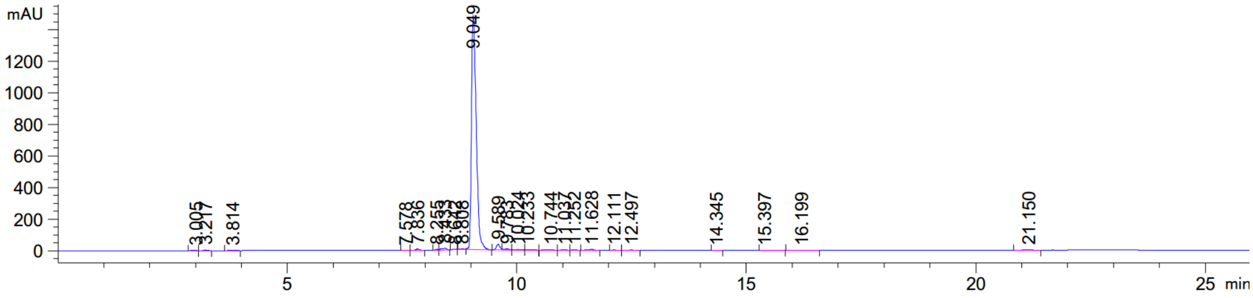

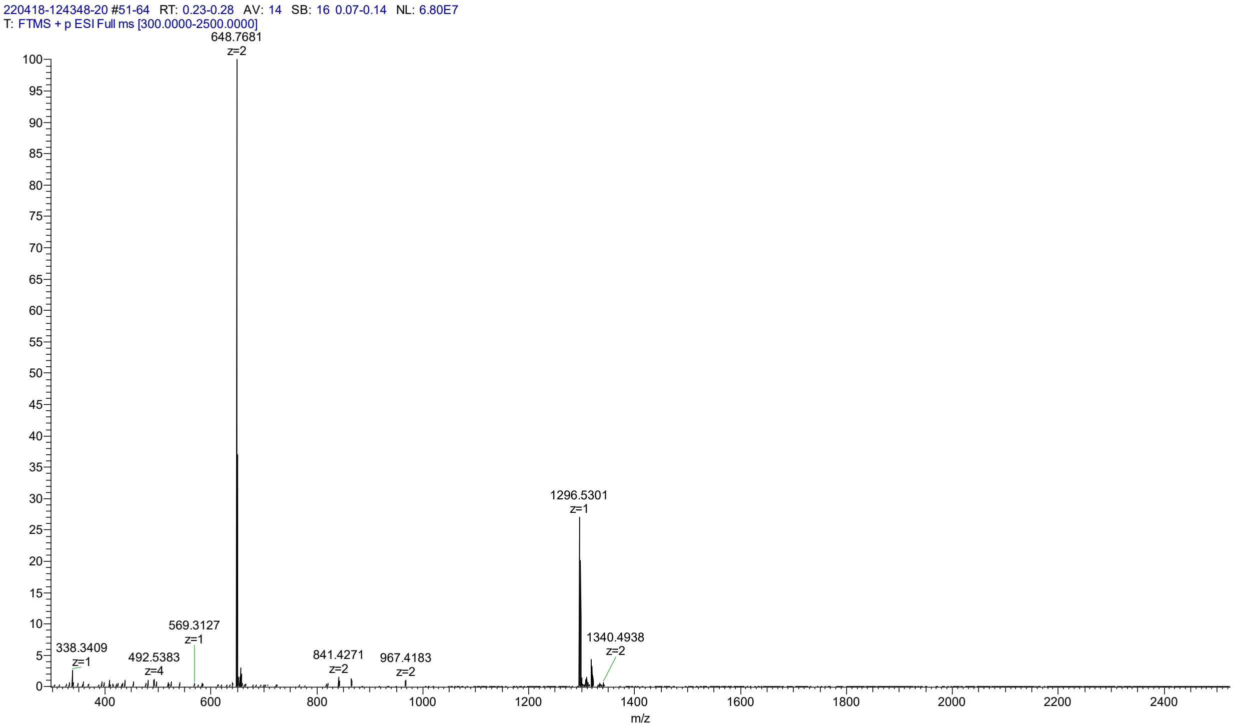

Supplementary Figure 79 Characterization of bicyclo 12-mer by analytical HPLC and high resolution MS. mAU refers to mini Arbitrary Unit.

bicyclo-D/A 12-mer retention time: 9.215 min calculated $[M+H]^+$: 1252.54 Observed $[M+H]^+$: 1252.5386

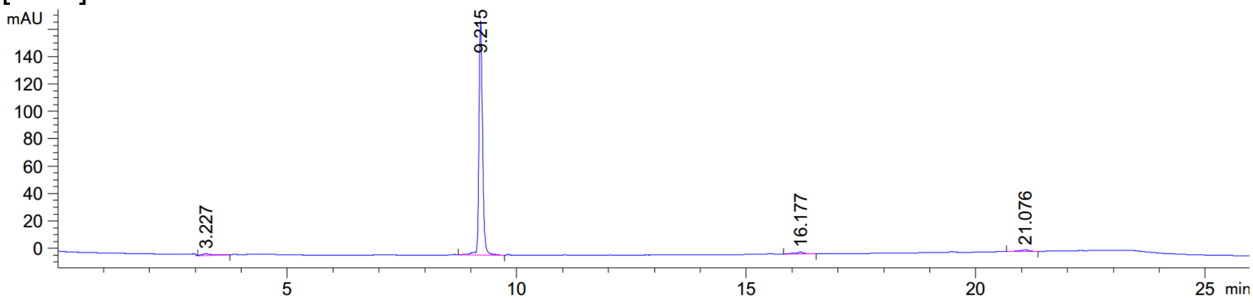

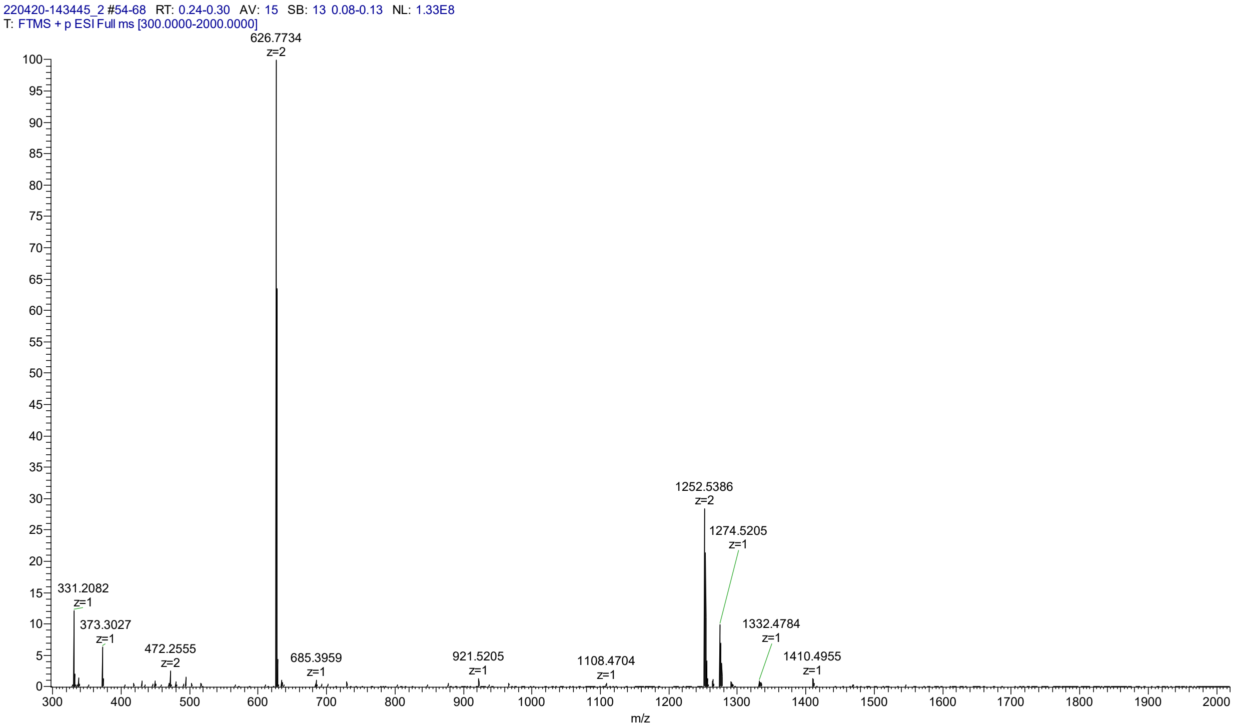

Supplementary Figure 80 Characterization of bicyclo-D/A 12-mer by analytical HPLC and high resolution MS. mAU refers to mini Arbitrary Unit.

bicyclo-D/N 12-mer retention time: 9.215 min calculated $[M+H]^+$: 1295.55 Observed $[M+H]^+$: 1295.5494

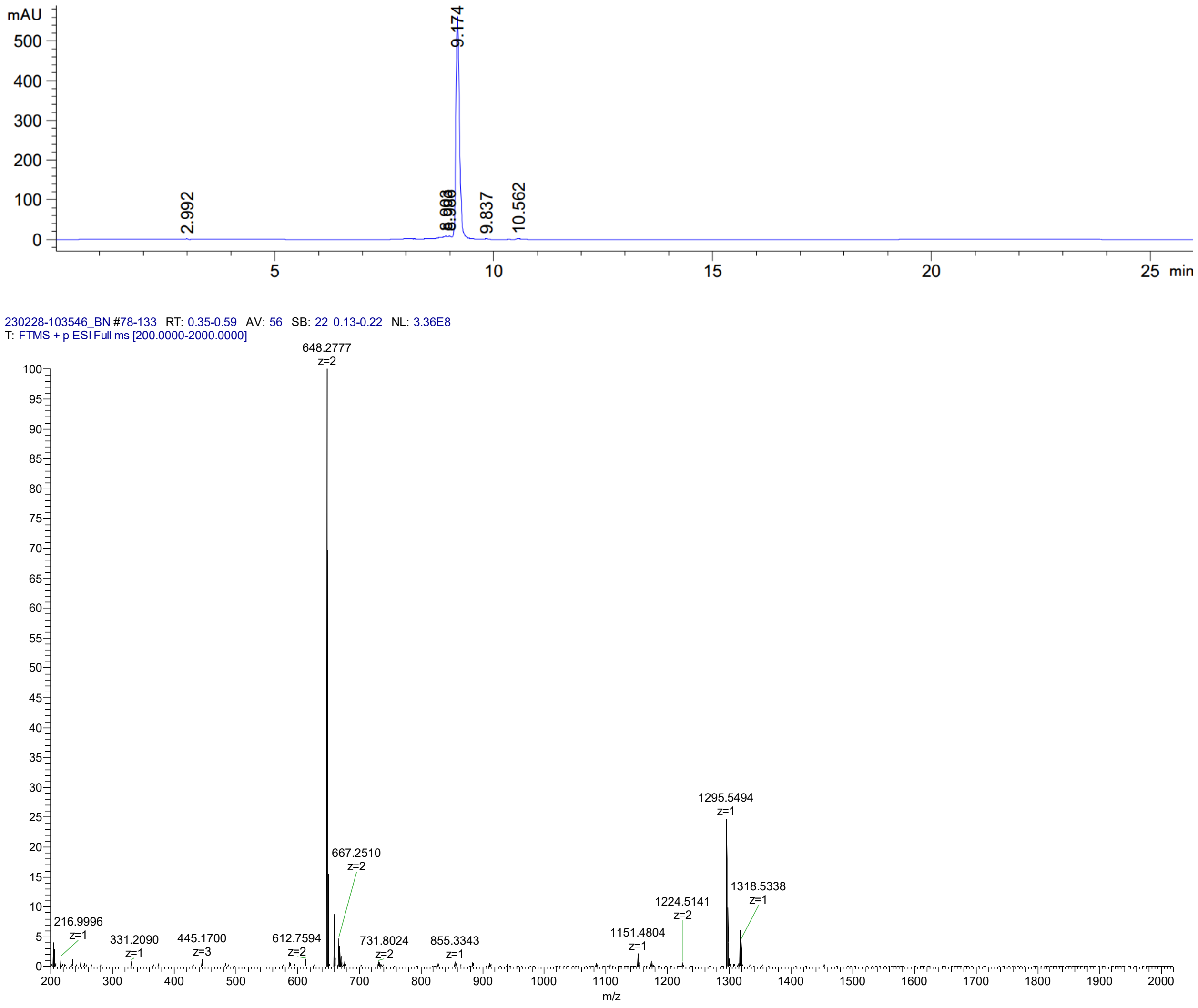

Supplementary Figure 81 Characterization of bicyclo-D/N 12-mer by analytical HPLC and high resolution MS. mAU refers to mini Arbitrary Unit.

[*N' – N3*] 12-mer retention time: 9.015 min calculated $[M+H]^+$: 1252.56 Observed $[M+H]^+$: 1252.5623

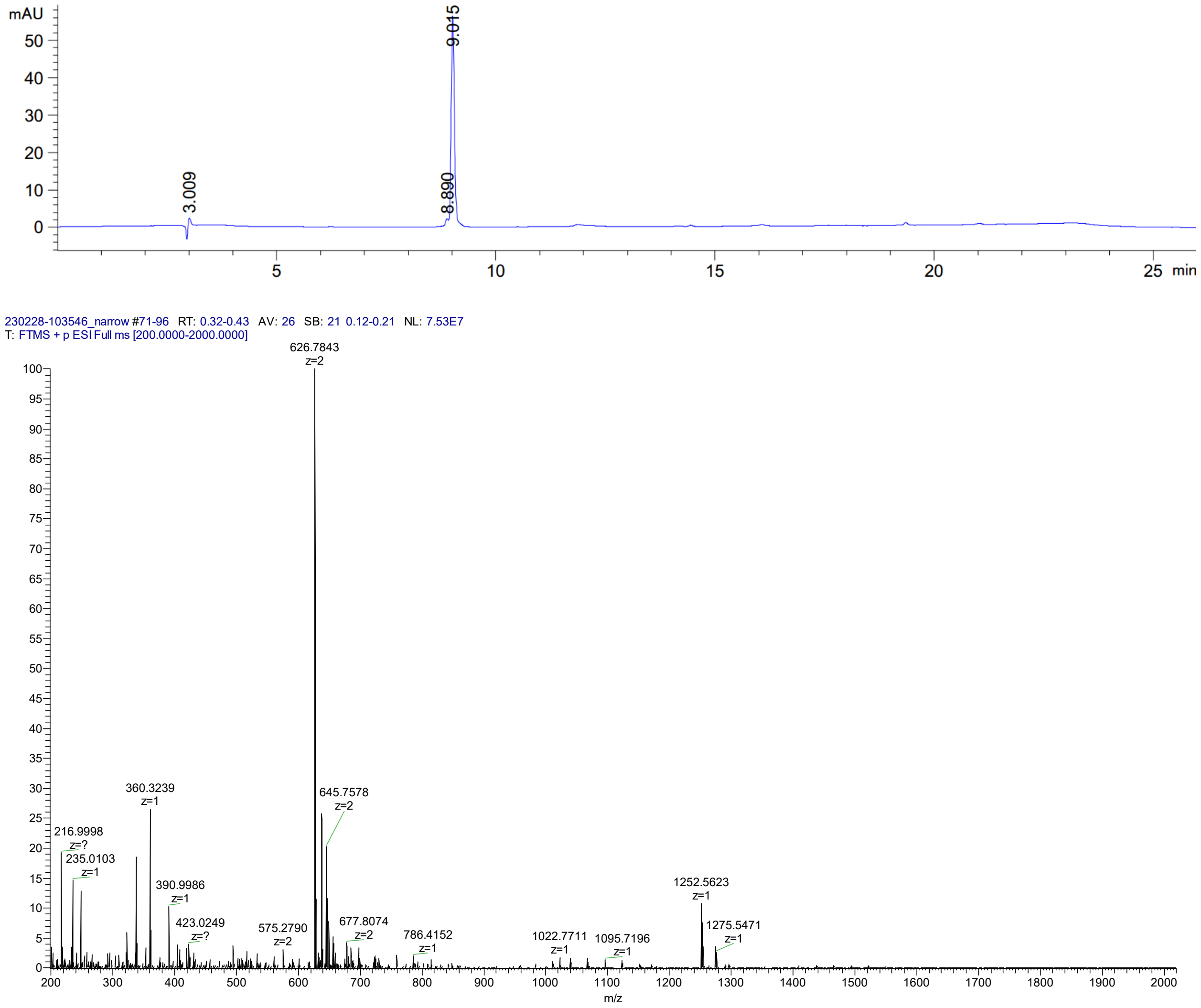

Supplementary Figure 82 Characterization of [*N' – N3*] 12-mer by analytical HPLC and high resolution MS. mAU refers to mini Arbitrary Unit.

[*N' – N4*] 12-mer retention time: 8.954 min calculated $[M+H]^+$: 1252.56 Observed $[M+H]^+$: 1252.5639

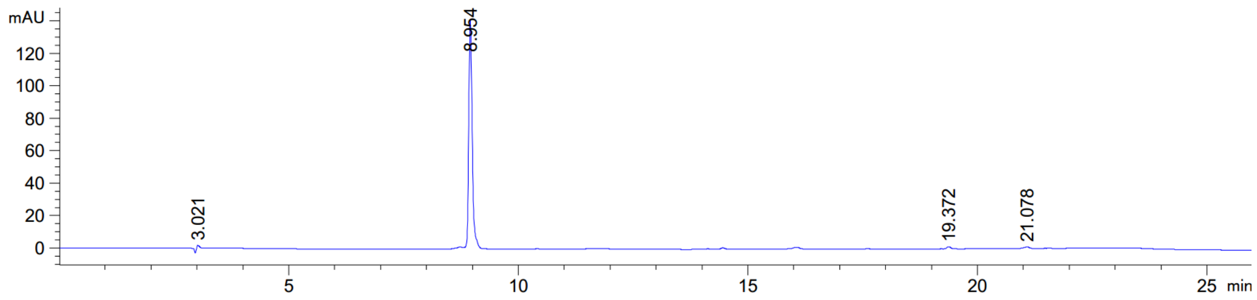

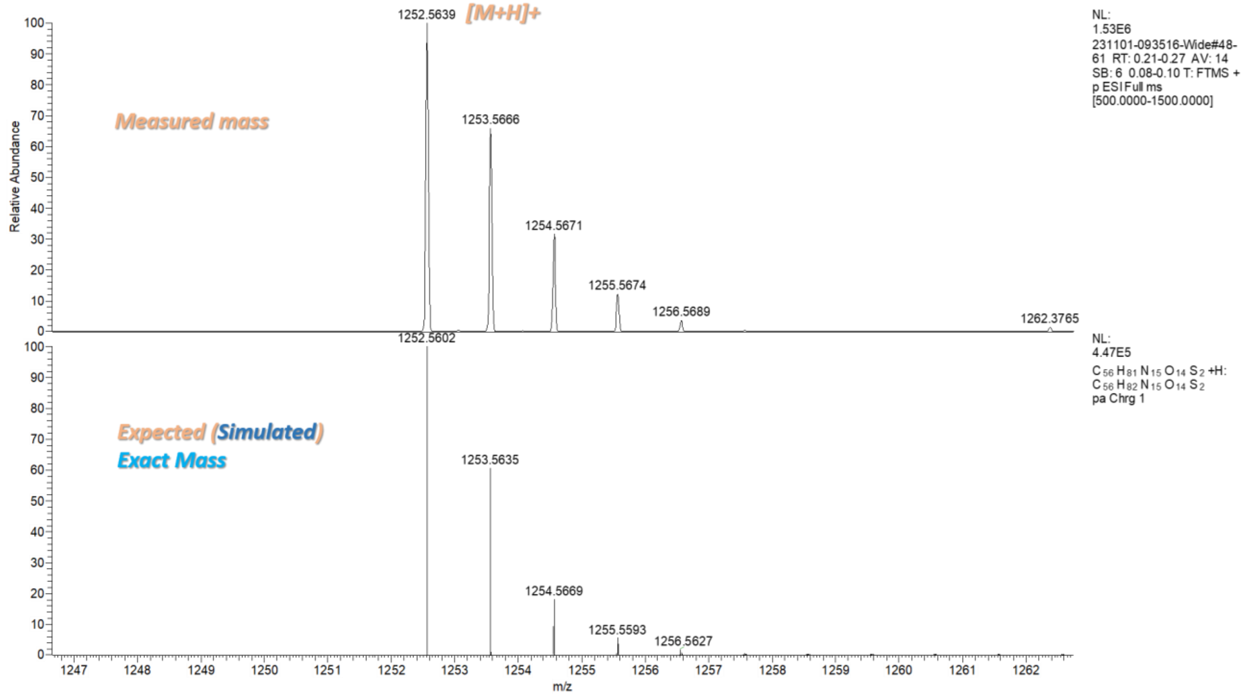

Supplementary Figure 83 Characterization of [*N' – N4*] 12-mer by analytical HPLC and high resolution MS. mAU refers to mini Arbitrary Unit.

1l retention time: 10.970 min calculated [M+H]$^{+}$: 1231.68 Observed [M+H]$^{+}$: 1231.6790

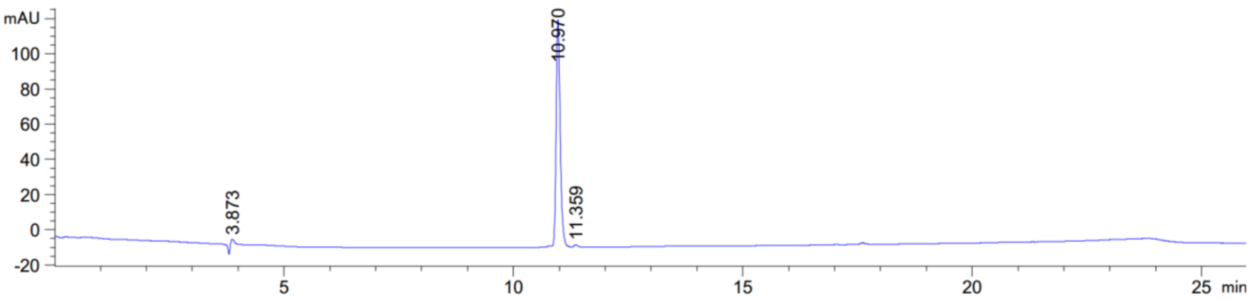

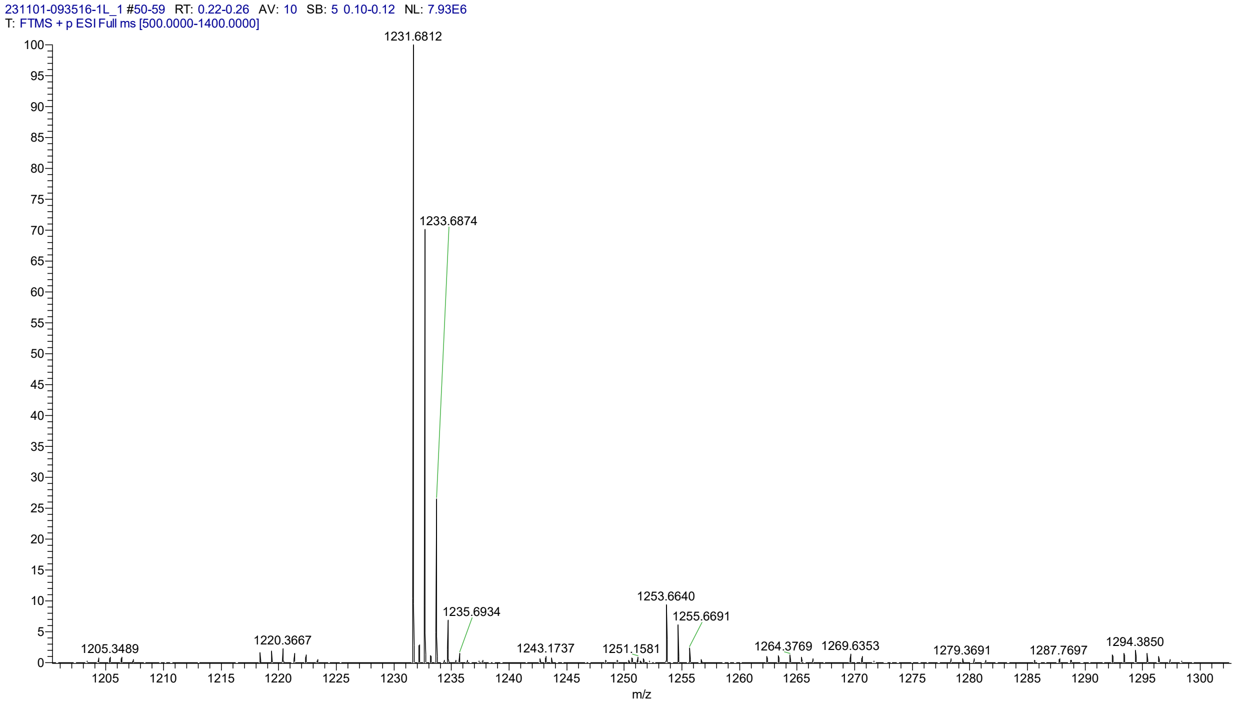

Supplementary Figure 84 Characterization of 1l by analytical HPLC and high resolution MS. mAU refers to mini Arbitrary Unit.

1B retention time: 11.008 min calculated $[M+H]^+$: 1500.63 Observed $[M+H]^+$: 1500.6332

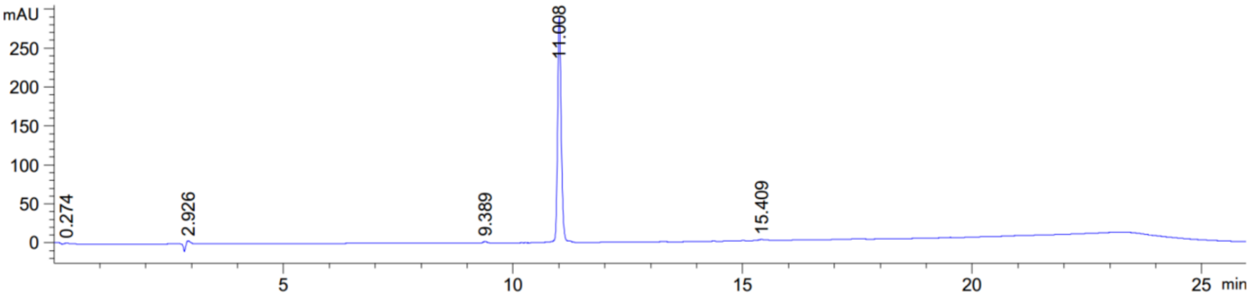

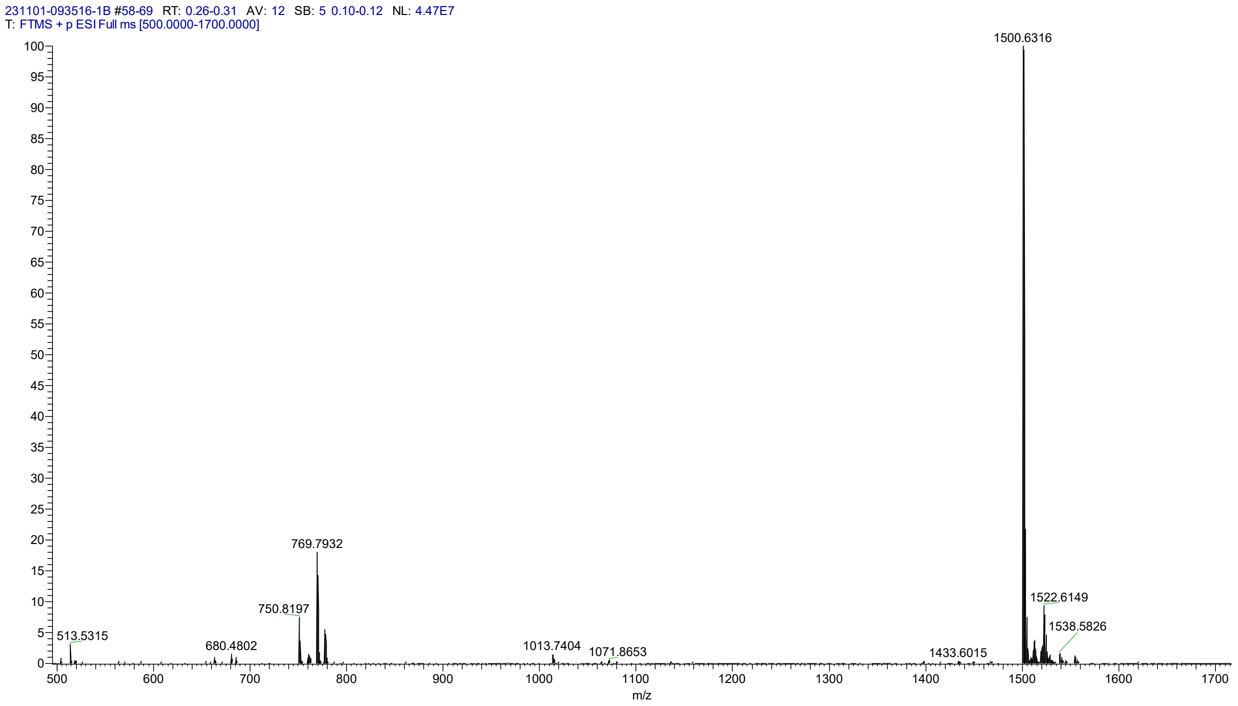

Supplementary Figure 85 Characterization of 1B by analytical HPLC and high resolution MS. mAU refers to mini Arbitrary Unit.

1m retention time: 10.315 min calculated $[M+H]^+$: 1428.63 Observed $[M+H]^+$: 1428.6325

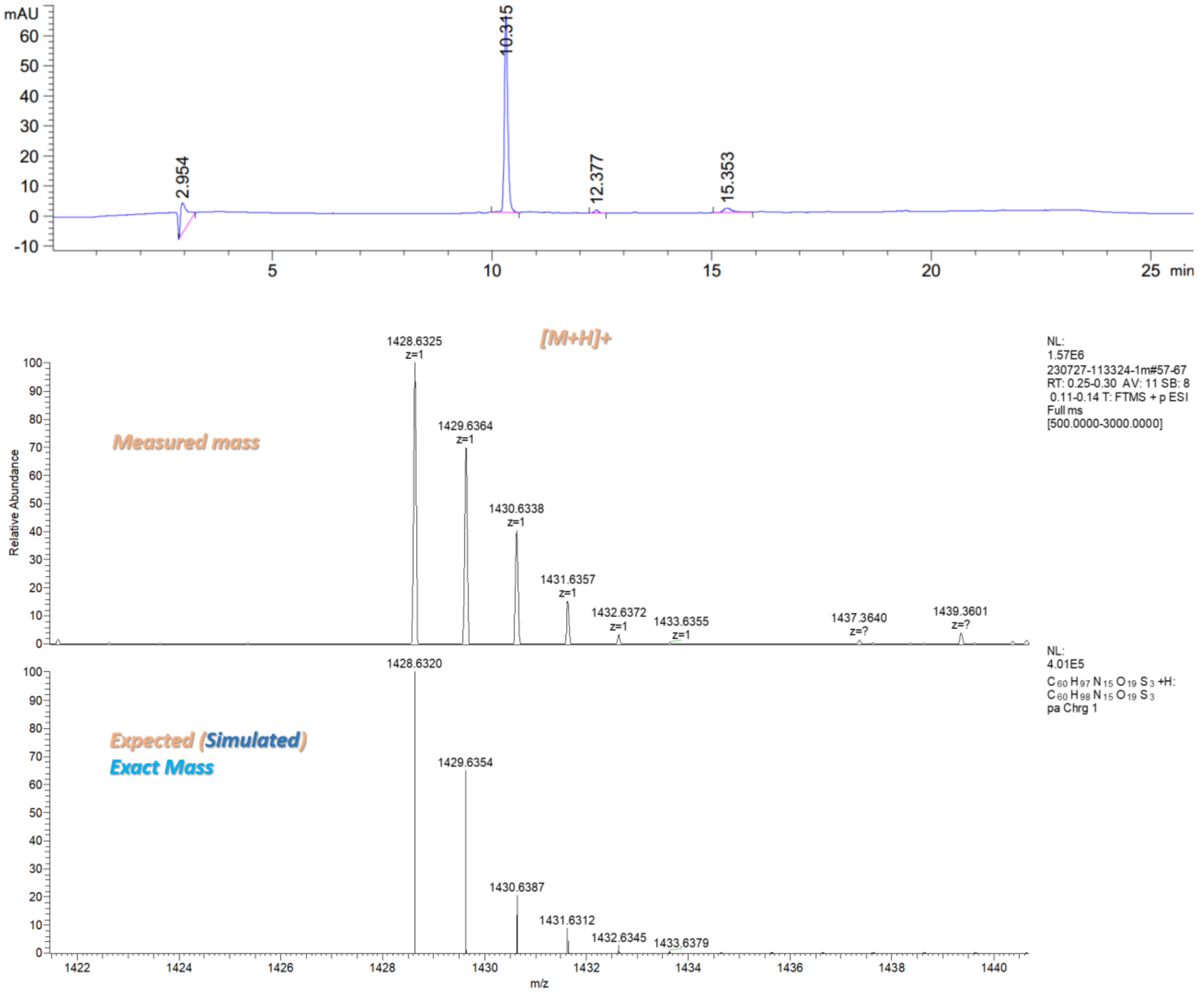

Supplementary Figure 86 Characterization of 1m by analytical HPLC and high resolution MS. mAU refers to mini Arbitrary Unit.

2l retention time: 11.549 min calculated $[M+H]^+$: 1489.77 Observed $[M+H]^+$: 1489.7646

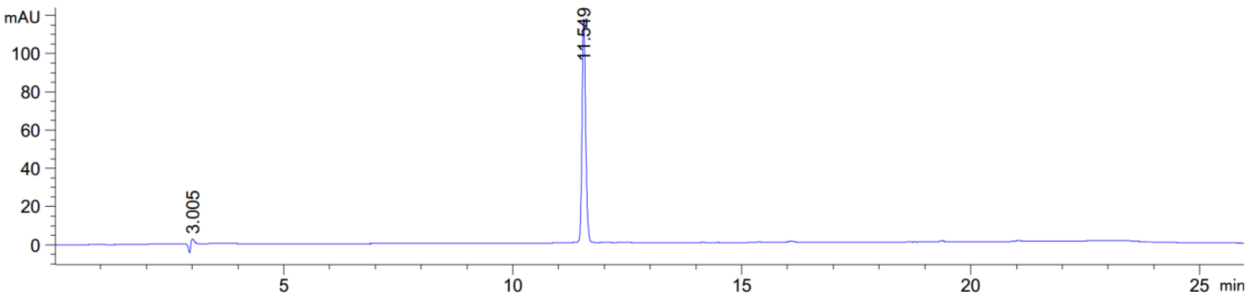

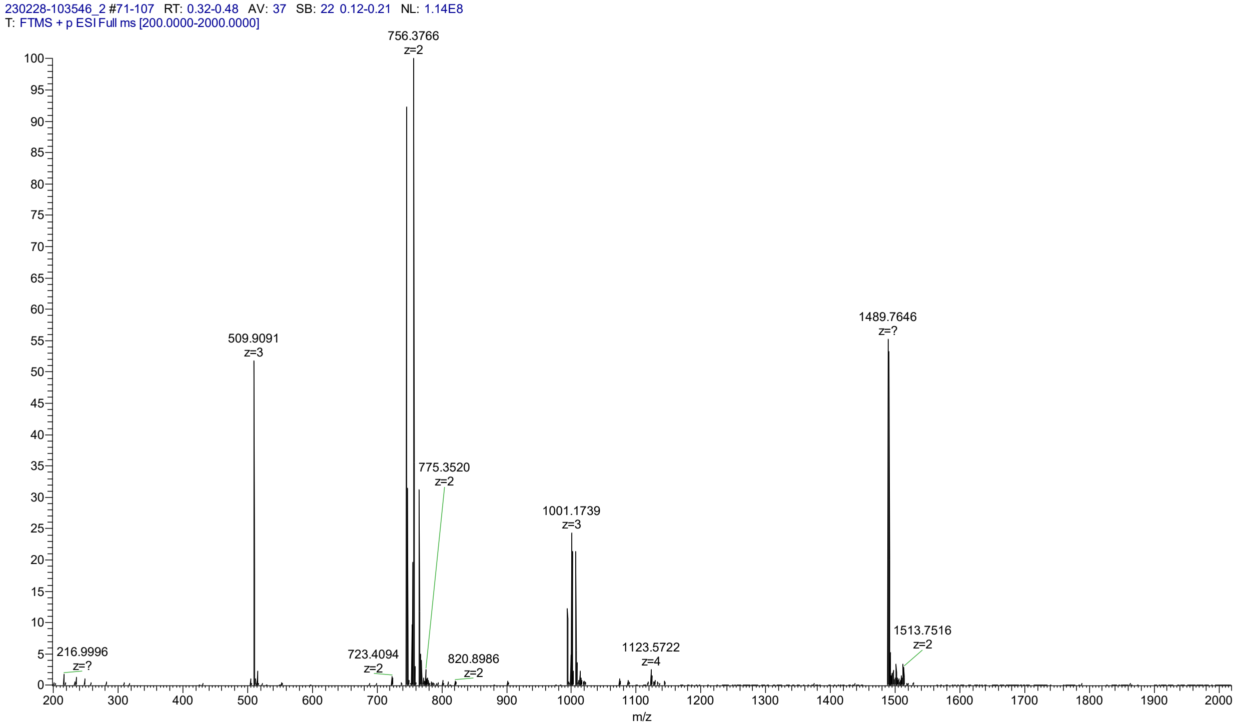

Supplementary Figure 87 Characterization of 2l by analytical HPLC and high resolution MS. mAU refers to mini Arbitrary Unit.

2B retention time: 11.864 min calculated [M+H]$^+$: 1727.77 Observed [M+H]$^+$: 1727.7731

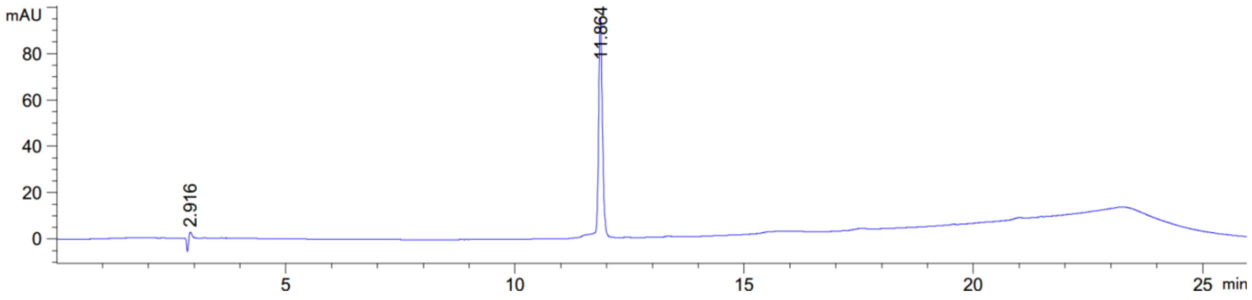

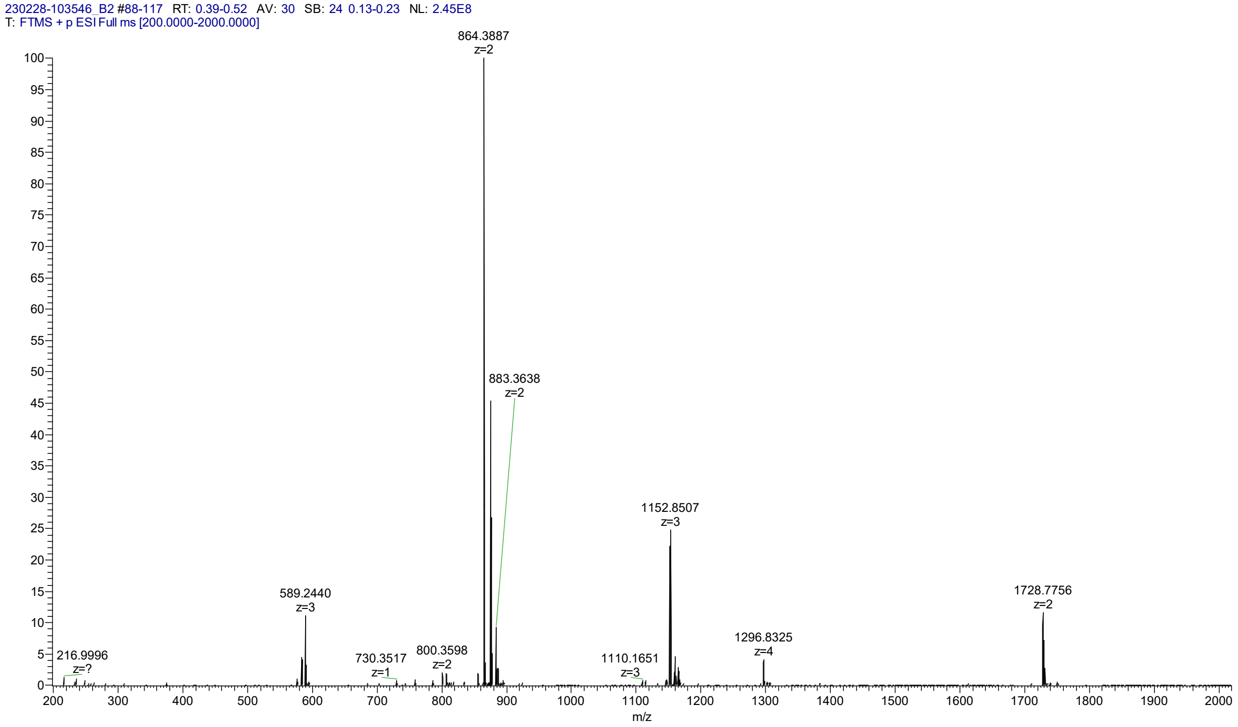

Supplementary Figure 88 Characterization of 2B by analytical HPLC and high resolution MS. mAU refers to mini Arbitrary Unit.

2m retention time: 11.379 min calculated $[M+H]^{+}$: 1655.77 Observed $[M+H]^{+}$: 1655.7688

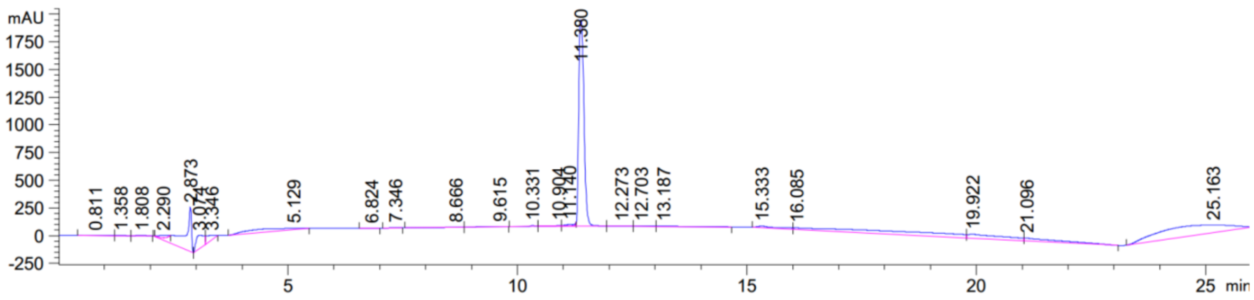

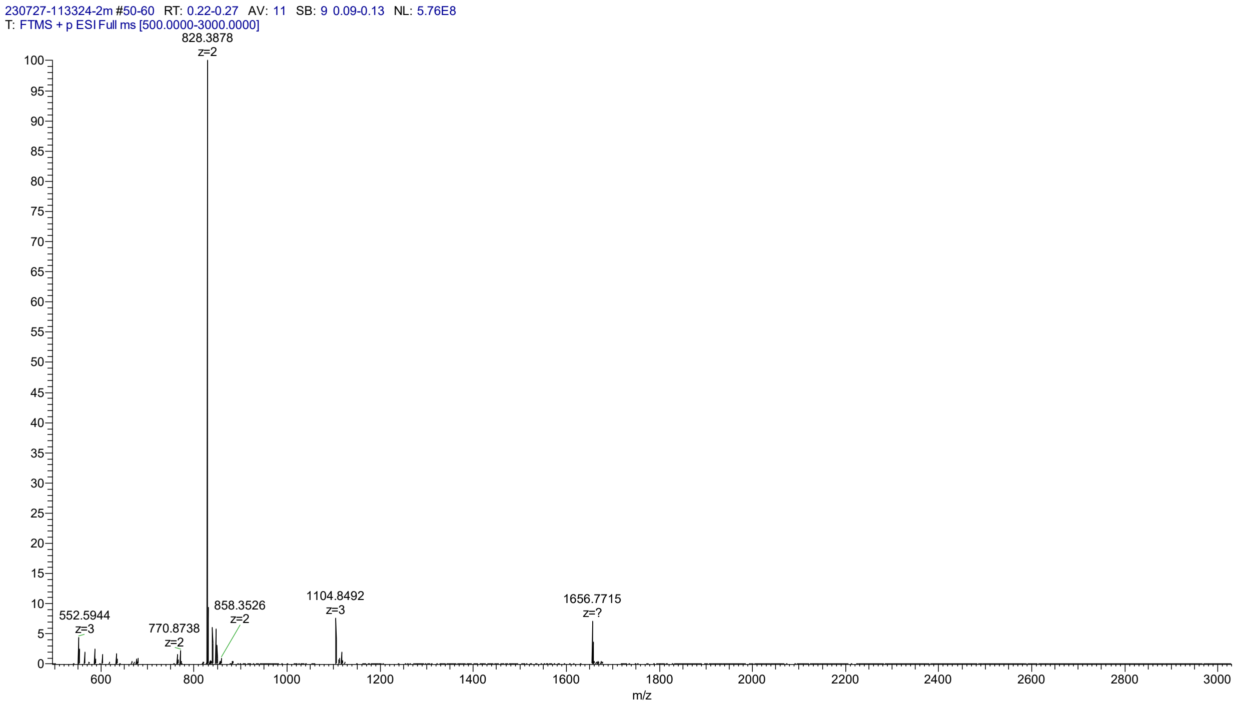

Supplementary Figure 89 Characterization of 2m by analytical HPLC and high resolution MS. mAU refers to mini Arbitrary Unit.

3l retention time: 11.049 min calculated $[M+H]^+$: 1634.91 Observed $[M+H]^+$: 1634.9097

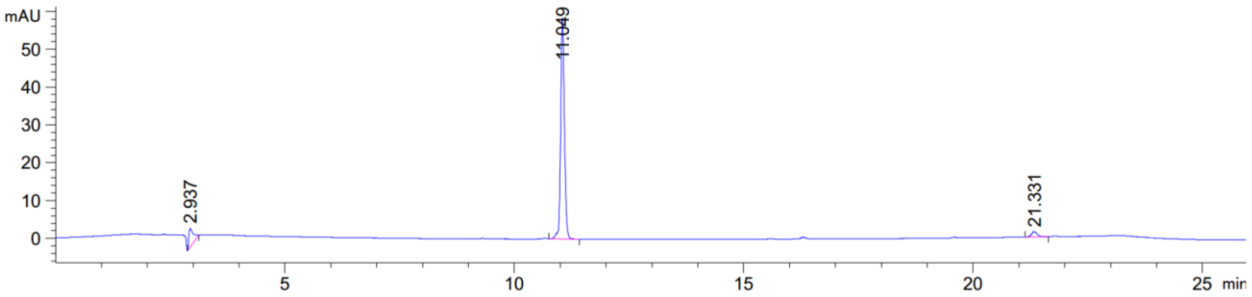

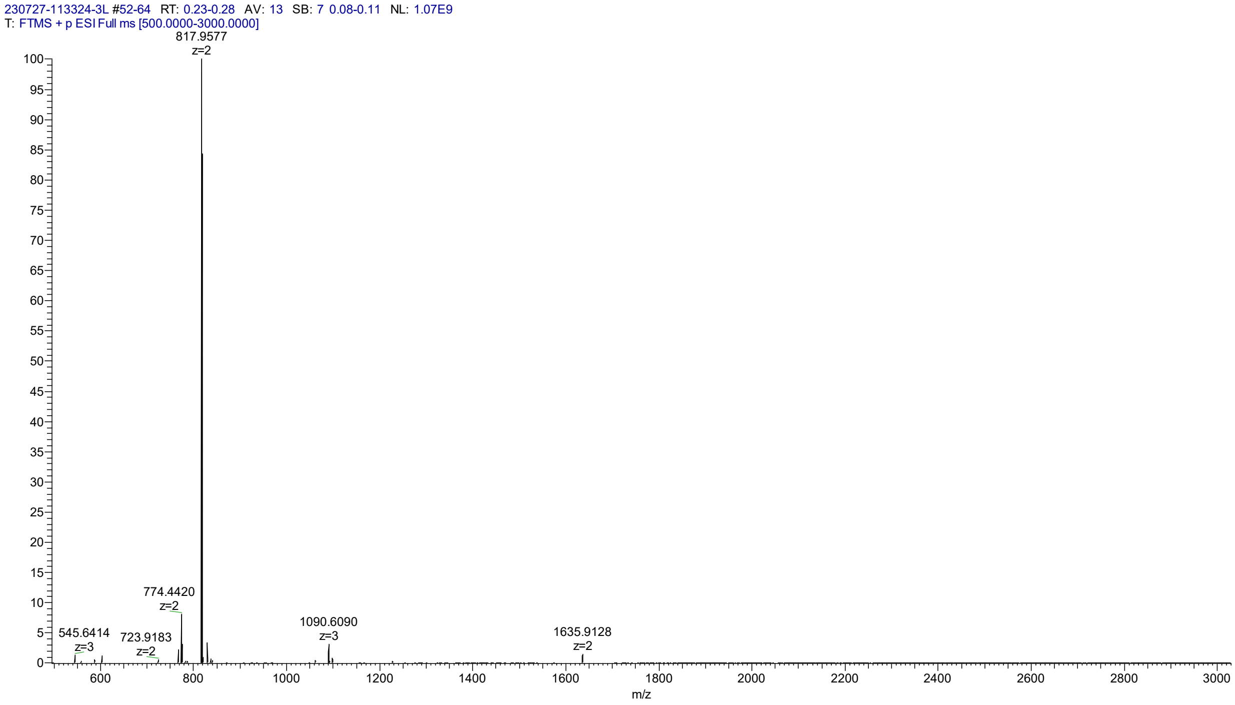

Supplementary Figure 90 Characterization of 3l by analytical HPLC and high resolution MS. mAU refers to mini Arbitrary Unit.

3B retention time: 11.394 min calculated [M+H]$^+$: 1958.92 Observed [M+H]$^+$: 1958.9244

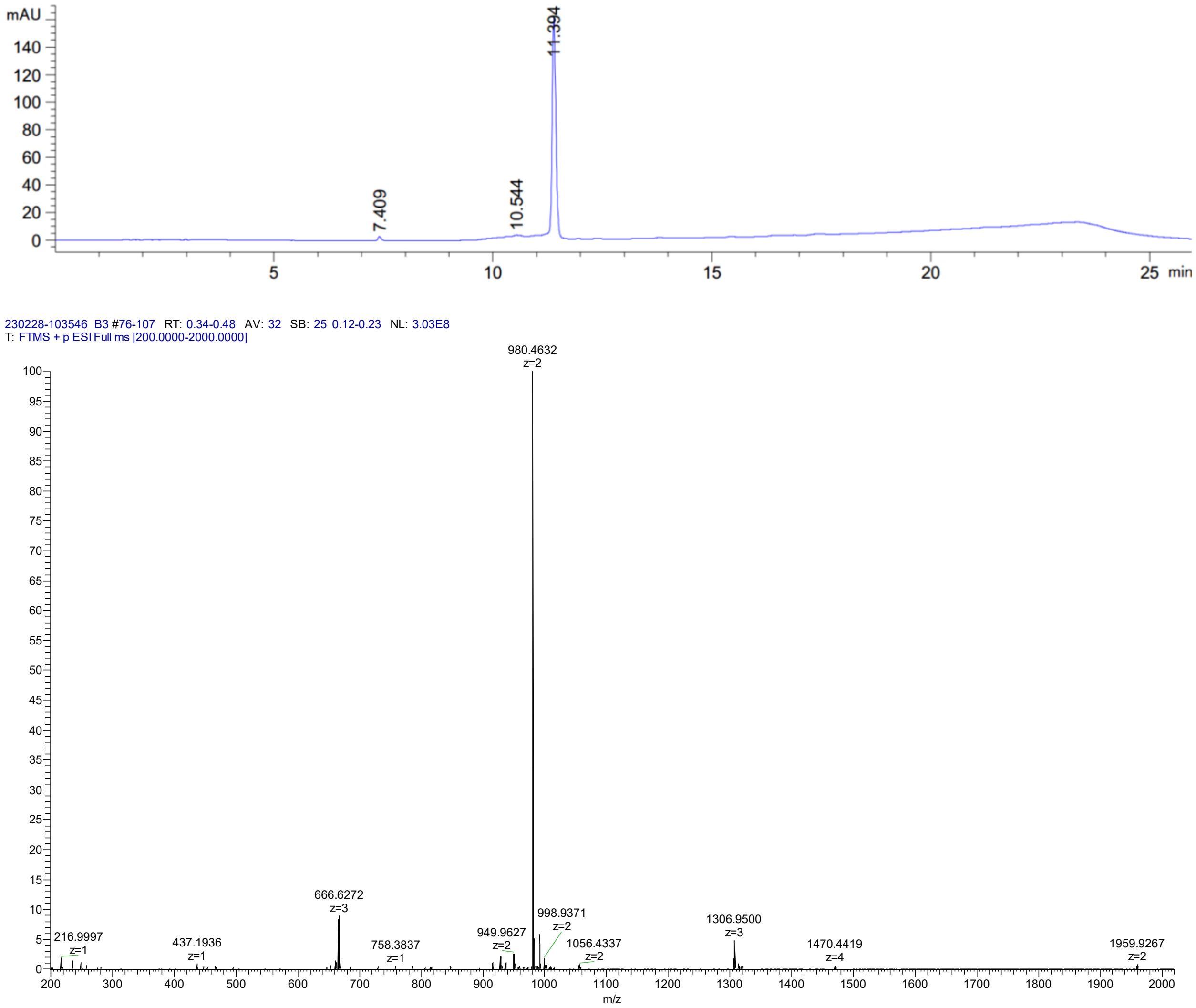

Supplementary Figure 91 Characterization of 3B by analytical HPLC and high resolution MS. mAU refers to mini Arbitrary Unit.

3m retention time: 10.799 min calculated $[M+H]^+$: 1886.92 Observed $[M+H]^+$: 1886.9194

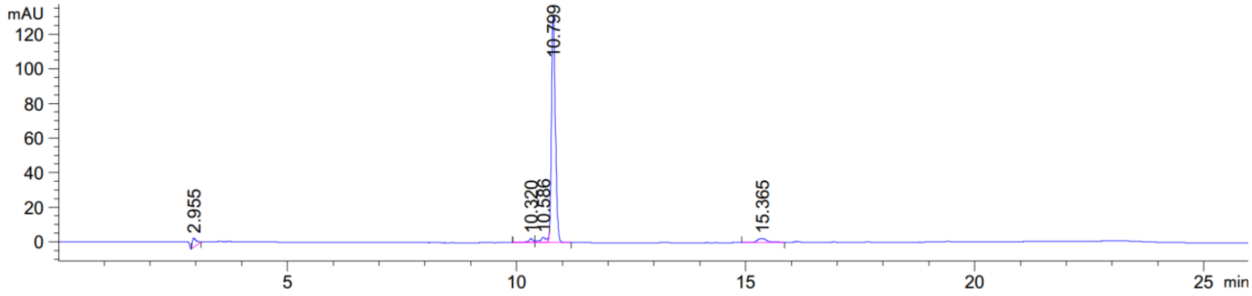

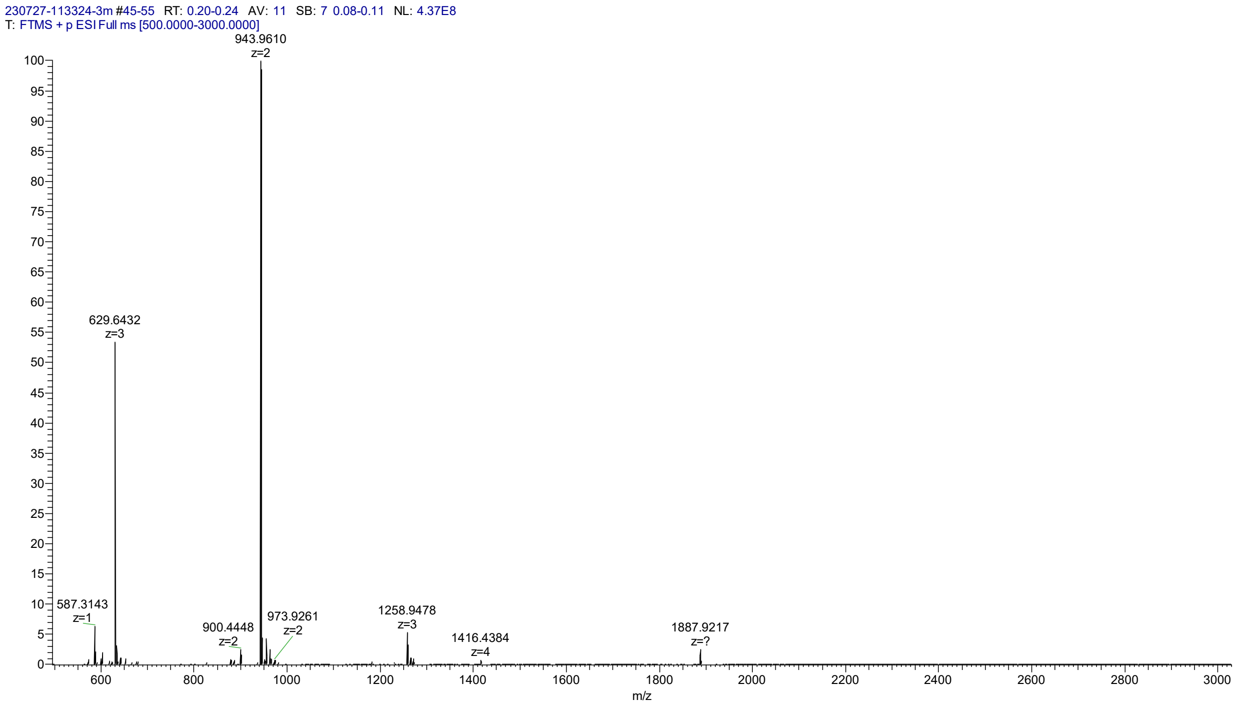

Supplementary Figure 92 Characterization of 3m by analytical HPLC and high resolution MS. mAU refers to mini Arbitrary Unit.

4l retention time: 9.819 min calculated $[M+H]^+$: 2309.38 Observed $[M+H]^+$: 2309.3697

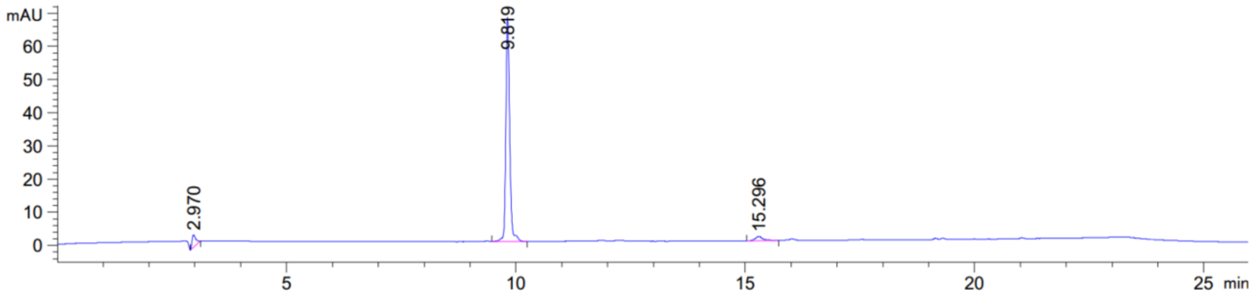

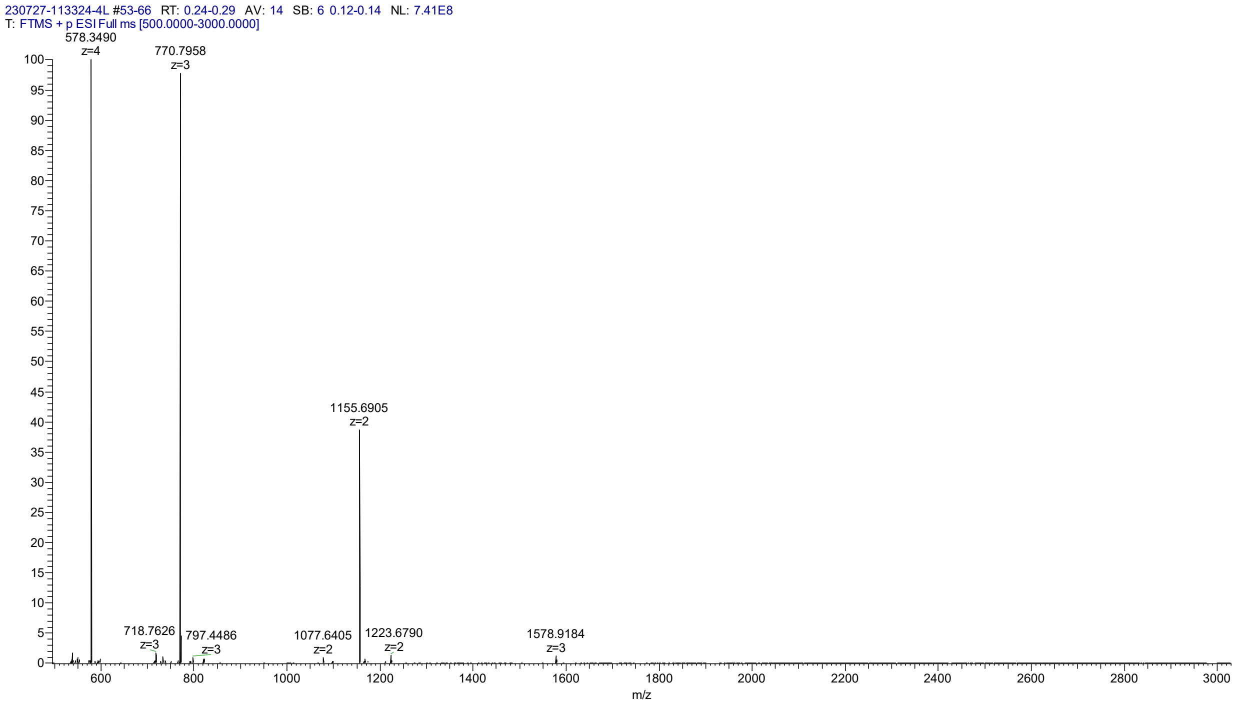

Supplementary Figure 93 Characterization of 4l by analytical HPLC and high resolution MS. mAU refers to mini Arbitrary Unit.

4B retention time: 10.854 min calculated $[M+2H]^{2+}$: 1474.27 calculated $[M+3H]^{3+}$: 983.19 calculated $[M+4H]^{4+}$: 737.64 Observed $[M+2H]^{2+}$: 1474.2785 Observed $[M+3H]^{3+}$: 983.1865 Observed $[M+4H]^{4+}$: 737.6416

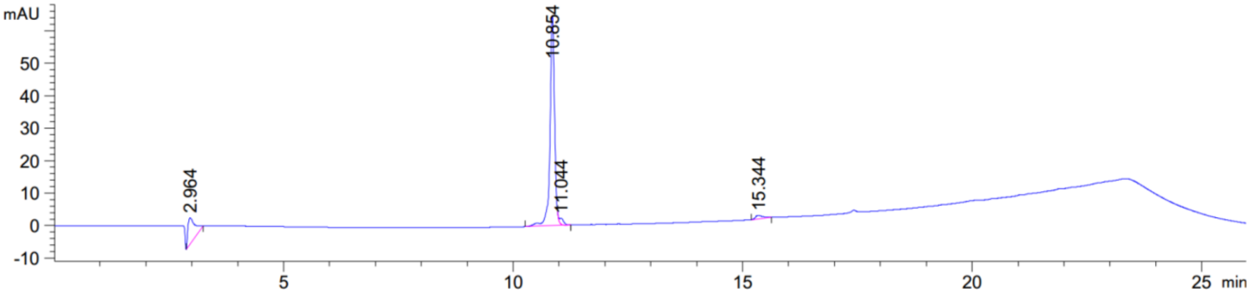

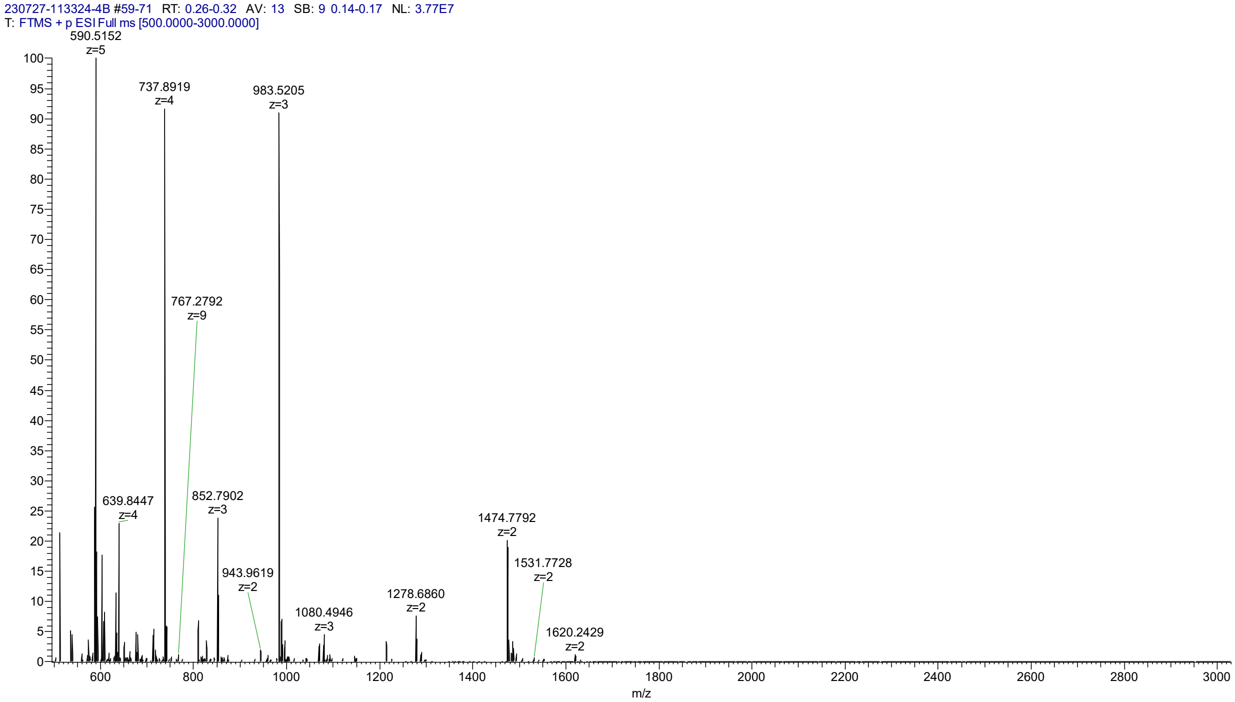

Supplementary Figure 94 Characterization of 4B by analytical HPLC and high resolution MS. mAU refers to mini Arbitrary Unit.

4m retention time: 10.238 min calculated $[M+2H]^{2+}$: 1438.27 calculated $[M+3H]^{3+}$: 959.19 calculated $[M+4H]^{4+}$: 719.64 Observed $[M+2H]^{2+}$: 1438.2726 Observed $[M+3H]^{3+}$: 959.1844 Observed $[M+4H]^{4+}$: 719.6400

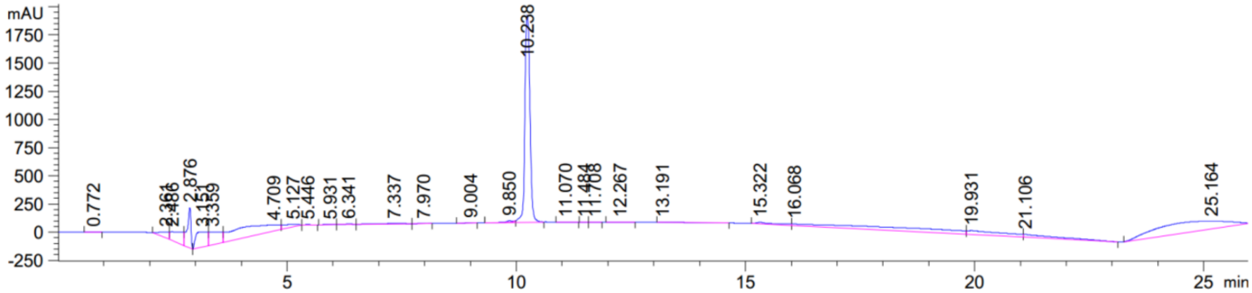

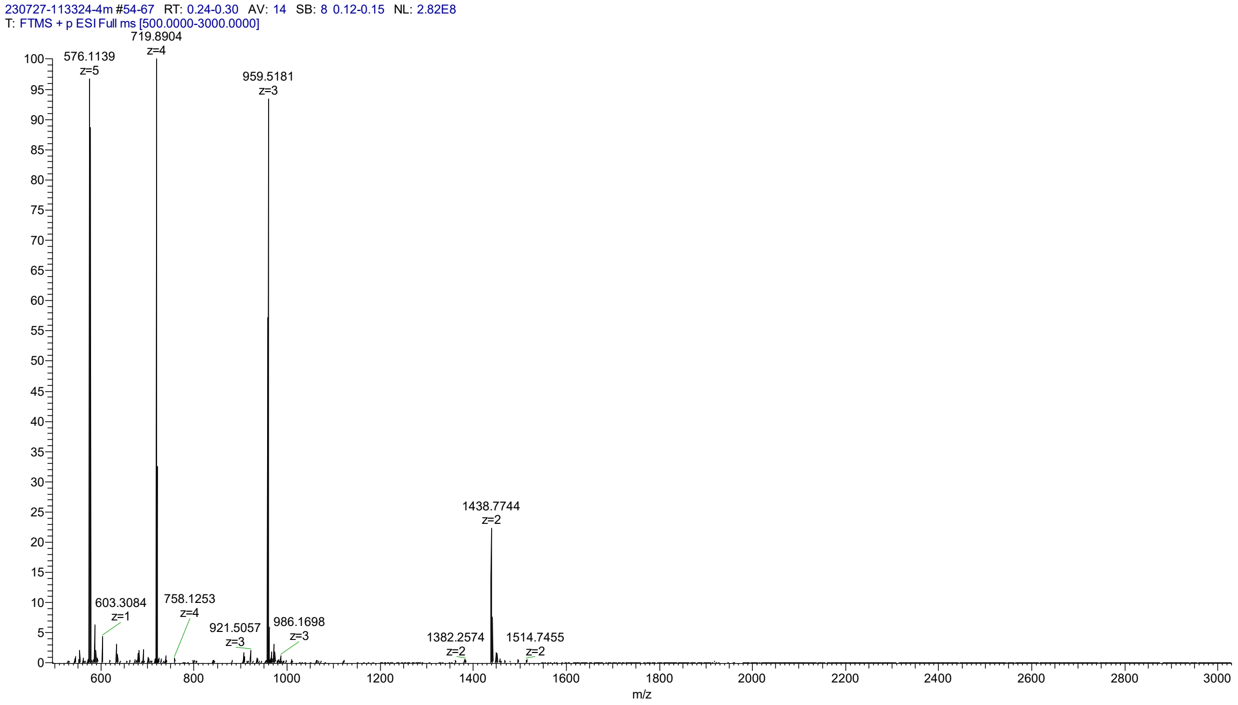

Supplementary Figure 95 Characterization of 4m by analytical HPLC and high resolution MS. mAU refers to mini Arbitrary Unit.

5l retention time: 12.313 min calculated [M+2H]$^{2+}$: 1595.27 calculated [M+3H]$^{3+}$: 1063.85 calculated [M+4H]$^{4+}$: 798.14 Observed [M+2H]$^{2+}$: 1595.2746 Observed [M+3H]$^{3+}$: 1063.8520 Observed [M+4H]$^{4+}$: 798.1407

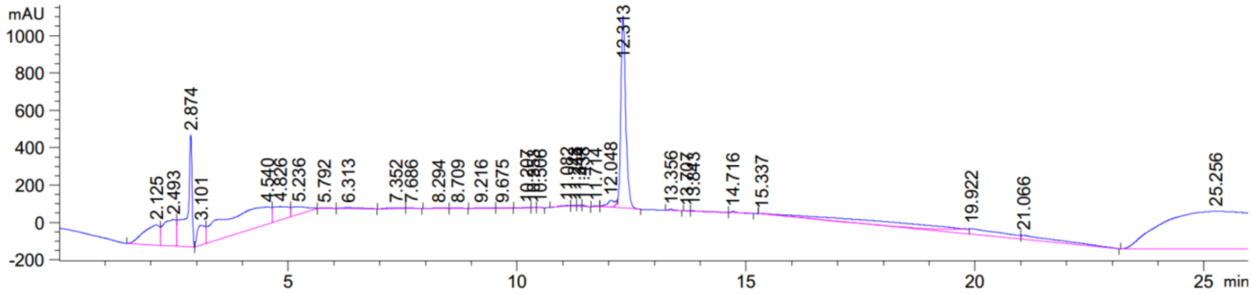

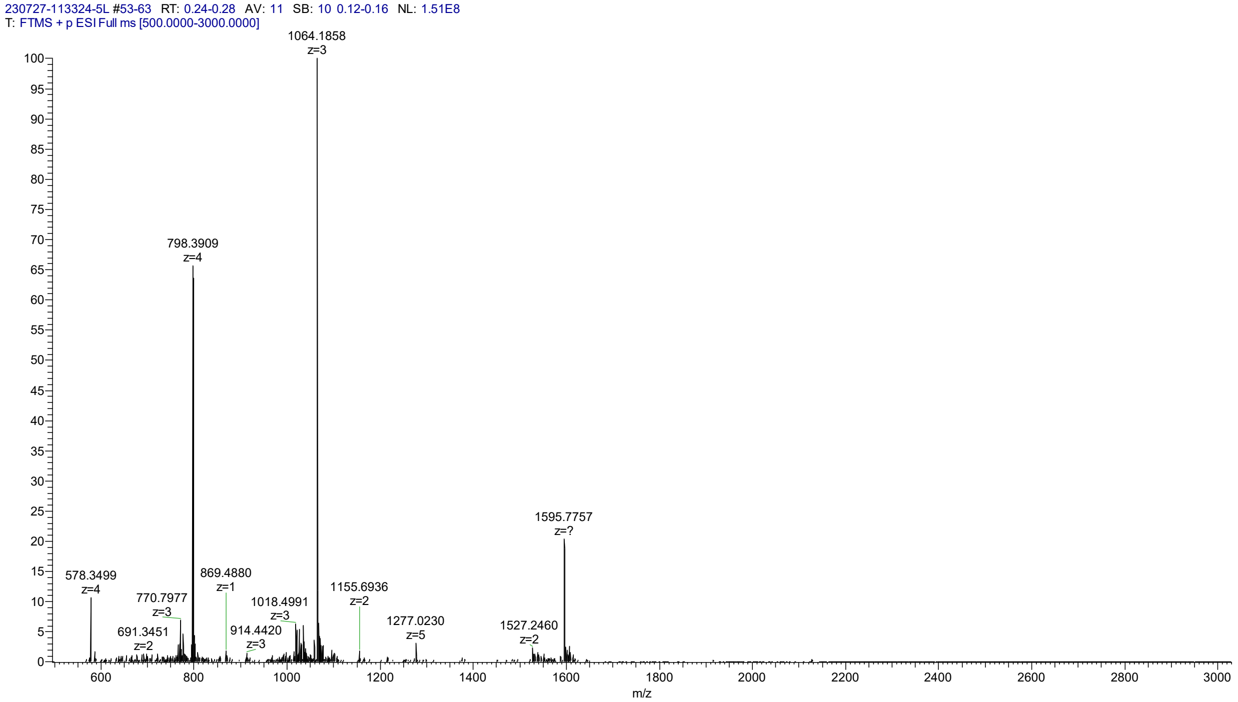

Supplementary Figure 96 Characterization of 5l by analytical HPLC and high resolution MS. mAU refers to mini Arbitrary Unit.

5B retention time: 12.074 min calculated $[M+2H]^{2+}$: 1655.74 calculated $[M+3H]^{3+}$: 1104.16 calculated $[M+4H]^{4+}$: 828.37 Observed $[M+2H]^{2+}$: 1655.7371 Observed $[M+3H]^{3+}$: 1104.1599 Observed $[M+4H]^{4+}$: 828.3714

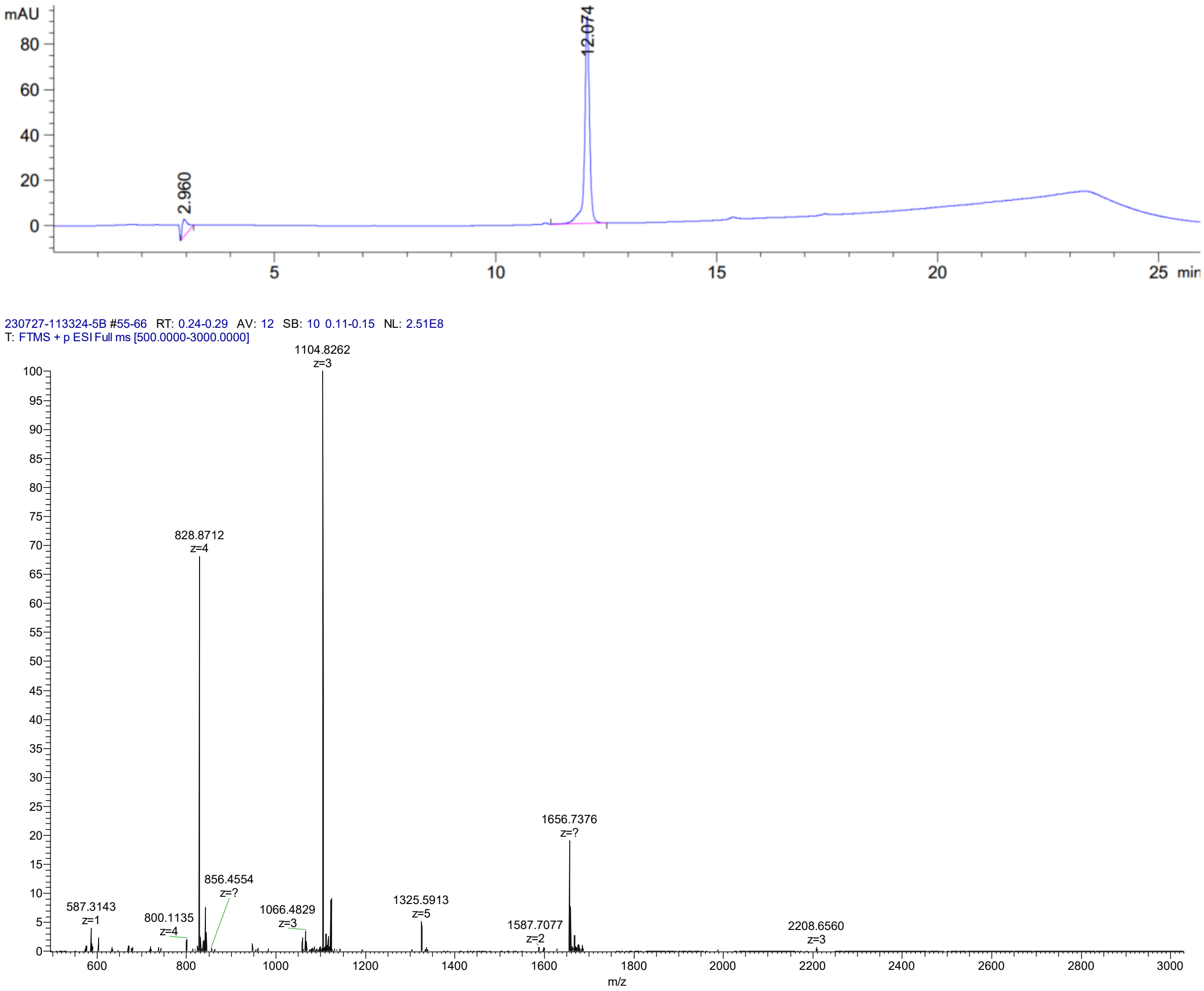

Supplementary Figure 97 Characterization of 5B by analytical HPLC and high resolution MS. mAU refers to mini Arbitrary Unit.

5m retention time: 11.700 min calculated $[M+2H]^{2+}$: 1619.74 calculated $[M+3H]^{3+}$: 1080.16 calculated $[M+4H]^{4+}$: 810.37 Observed $[M+2H]^{2+}$: 1619.7367 Observed $[M+3H]^{3+}$: 1080.1604 Observed $[M+4H]^{4+}$: 810.3719

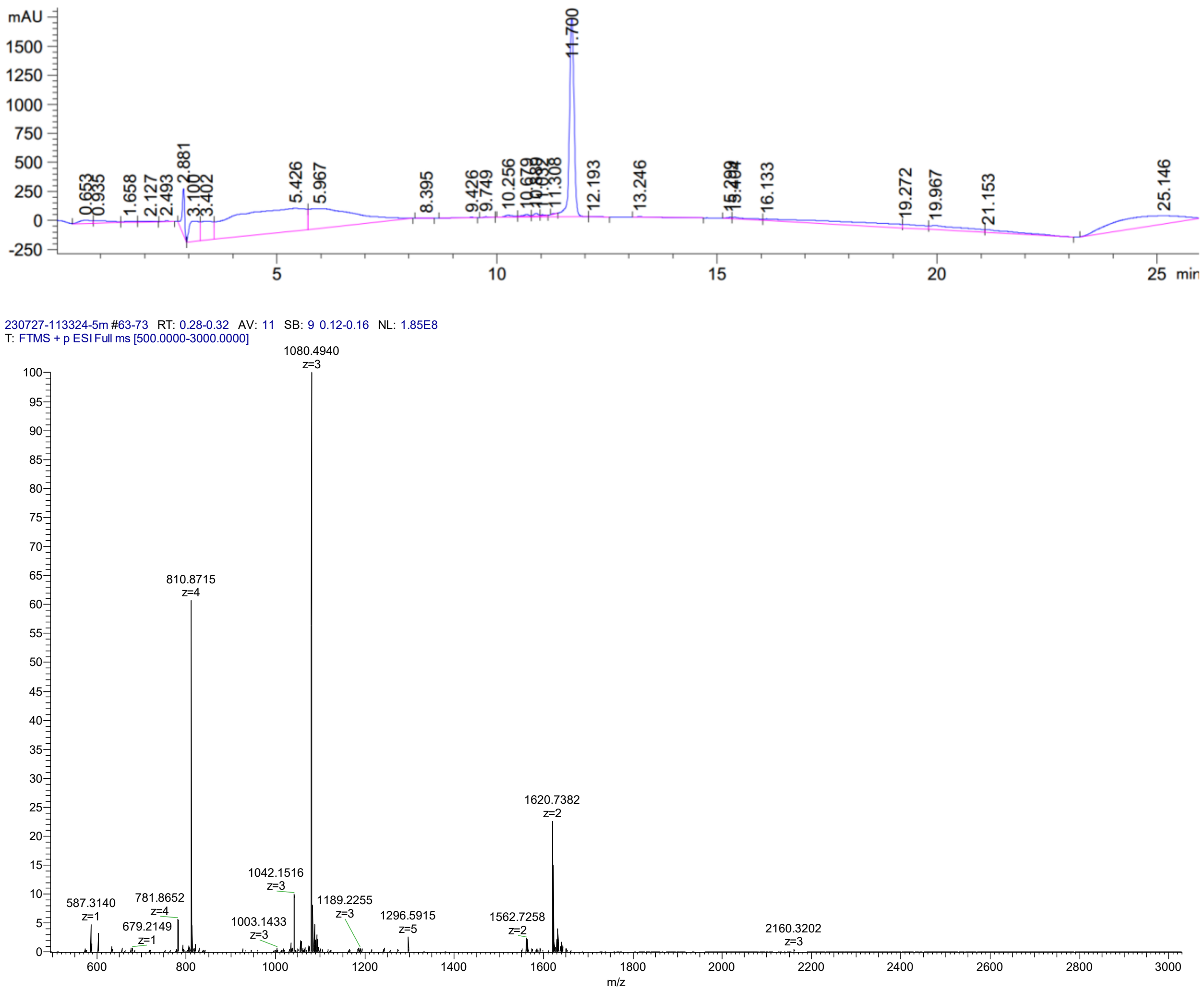

Supplementary Figure 98 Characterization of 5m by analytical HPLC and high resolution MS. mAU refers to mini Arbitrary Unit.

3lf retention time: 11.932 min calculated $[M+H]^+$: 2195.05 calculated $[M+2H]^{2+}$: 1098.03 Observed $[M+H]^+$: 2195.0451 Observed $[M+2H]^{2+}$: 1098.0259

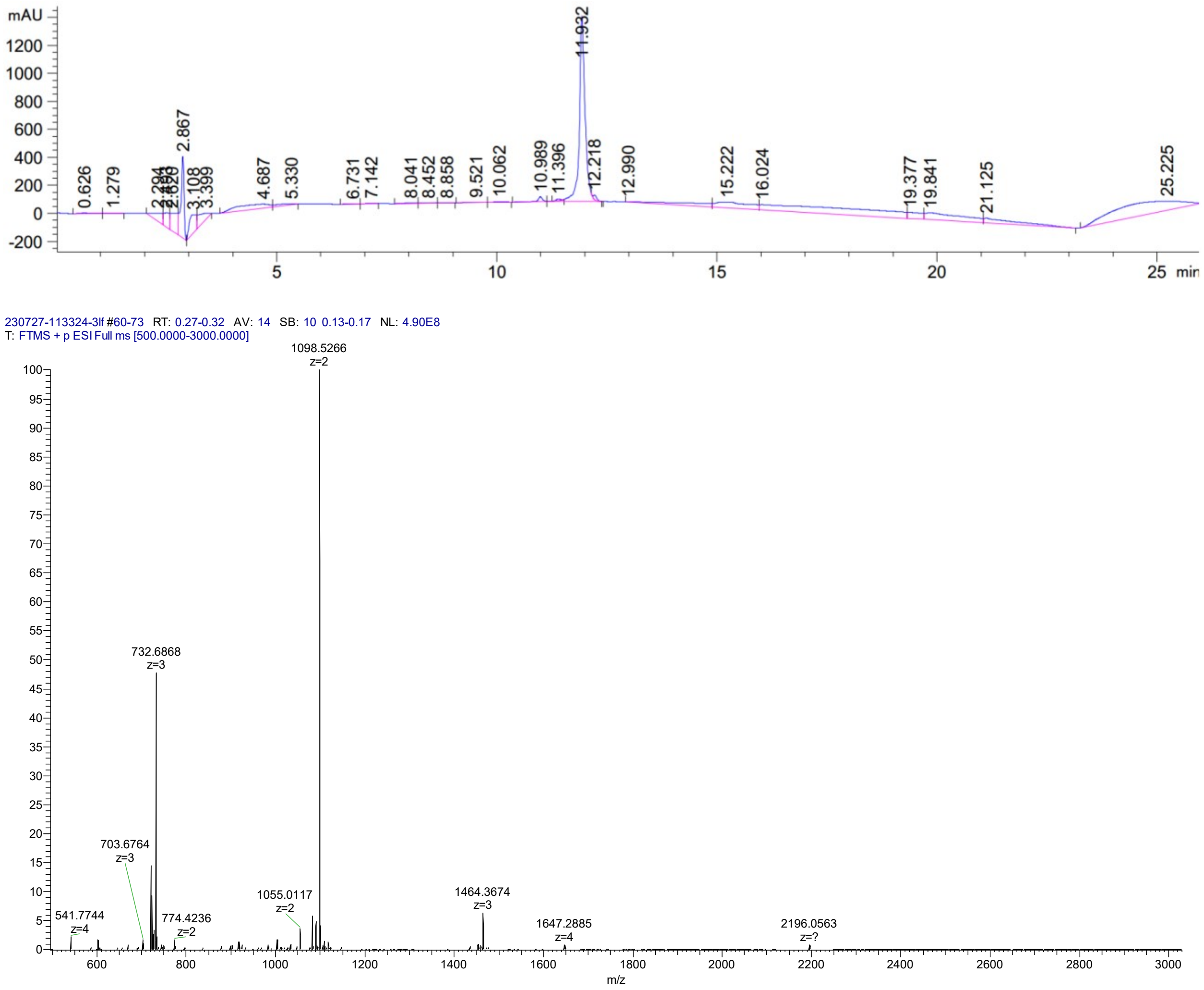

Supplementary Figure 99 Characterization of 3lf by analytical HPLC and high resolution MS. mAU refers to mini Arbitrary Unit.

3Bf retention time: 13.173 min calculated $[M+2H]^{2+}$: 1281.04 calculated $[M+3H]^{3+}$: 854.36 Observed $[M+2H]^{2+}$: 1281.0407 Observed $[M+3H]^{3+}$: 854.3612

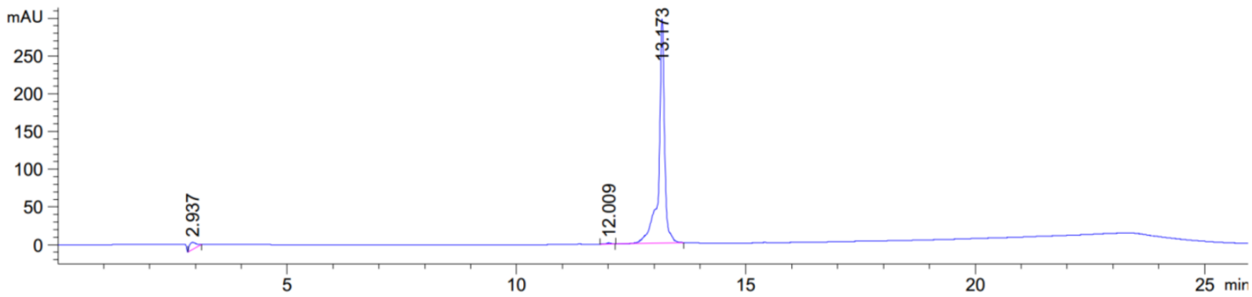

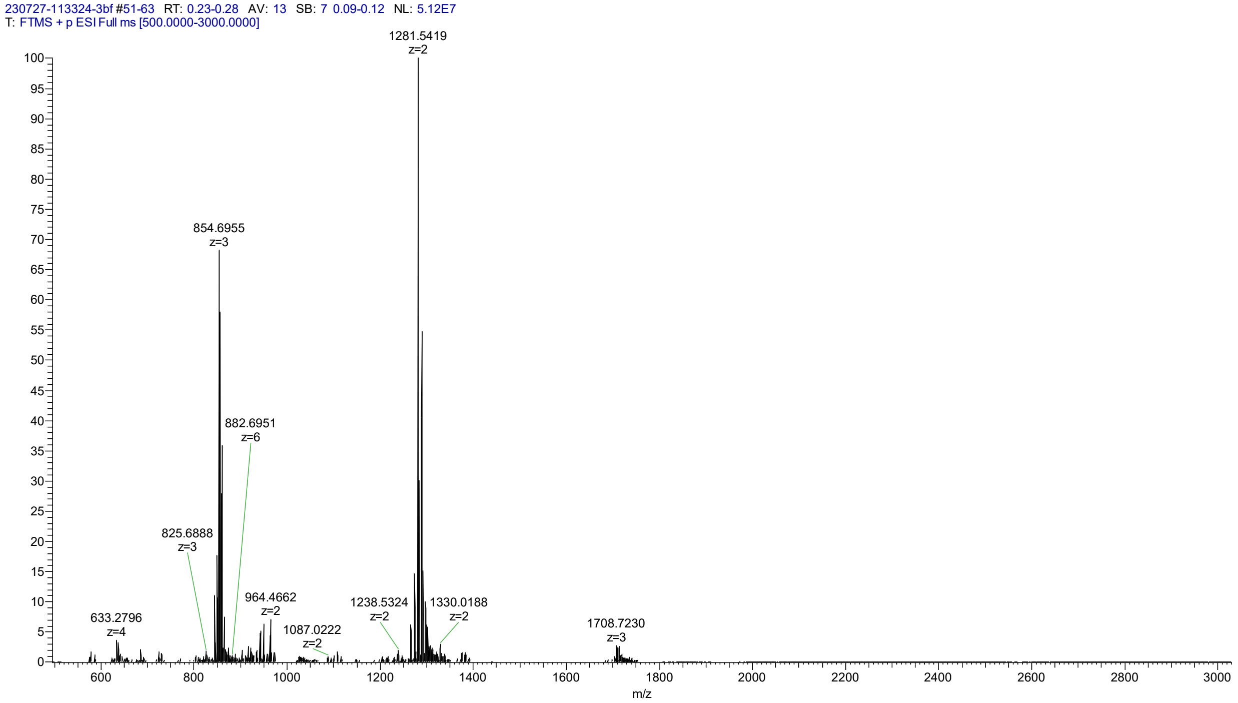

Supplementary Figure 100 Characterization of 3Bf by analytical HPLC and high resolution MS. mAU refers to mini Arbitrary Unit.

3mf retention time: 12.348 min calculated $[M+2H]^{2+}$: 1245.04 calculated $[M+3H]^{3+}$: 830.36 Observed $[M+2H]^{2+}$: 1245.0357 Observed $[M+3H]^{3+}$: 830.3592

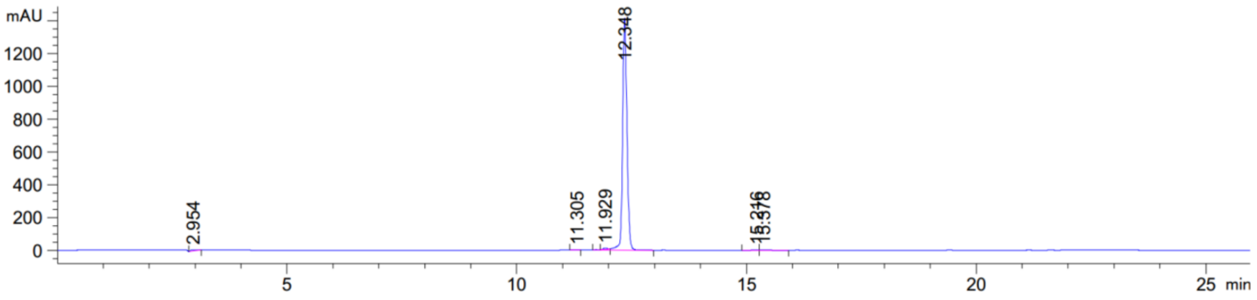

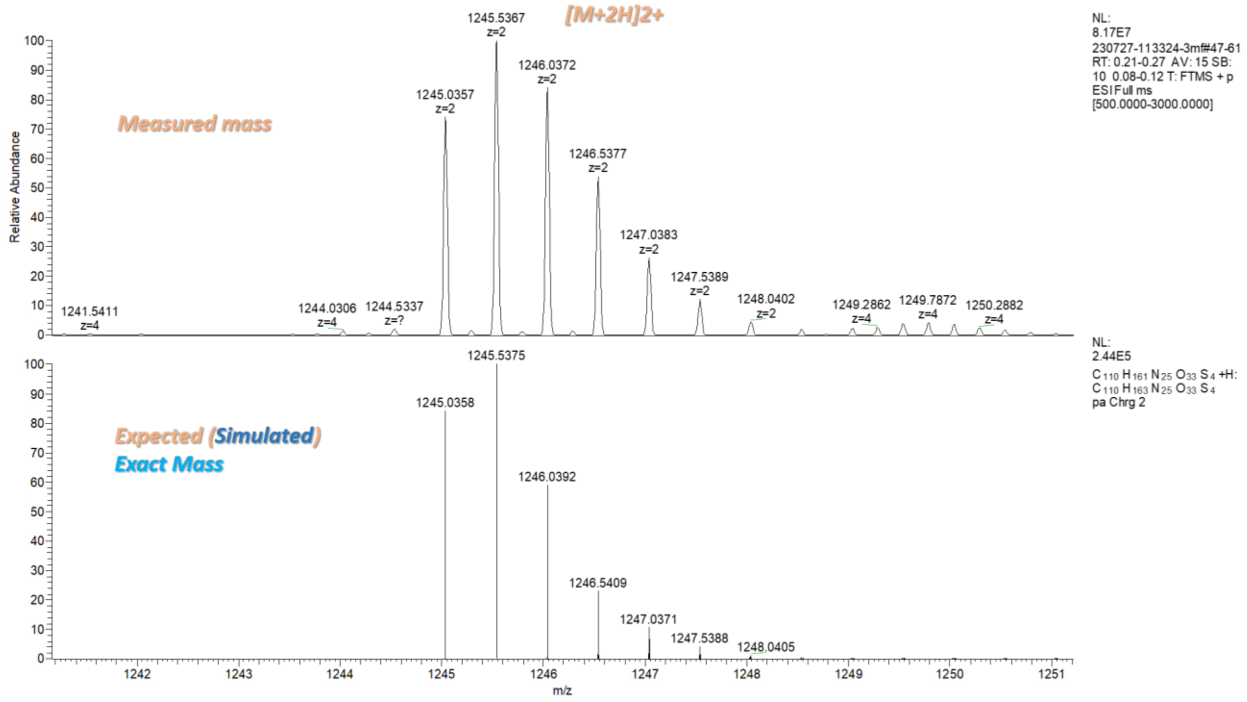

Supplementary Figure 101 Characterization of 3mf by analytical HPLC and high resolution MS. mAU refers to mini Arbitrary Unit.

BSM C-capped 12-mer retention time: 12.115 min calculated $[M+H]^+$: 1280.54 Observed $[M+H]^+$: 1280.5364

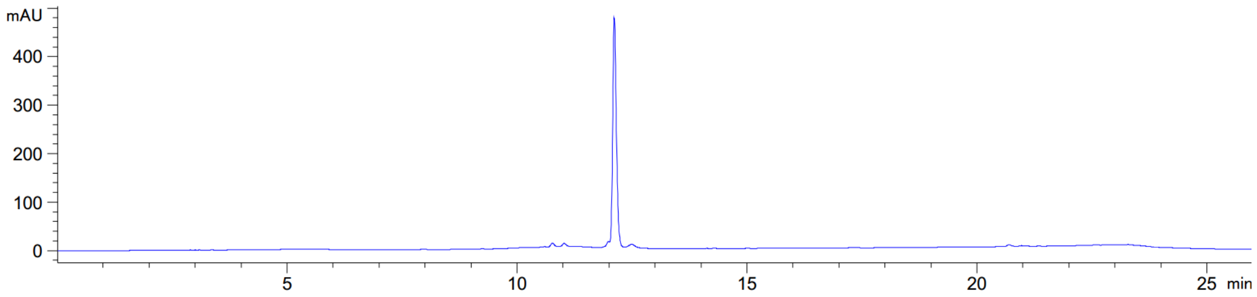

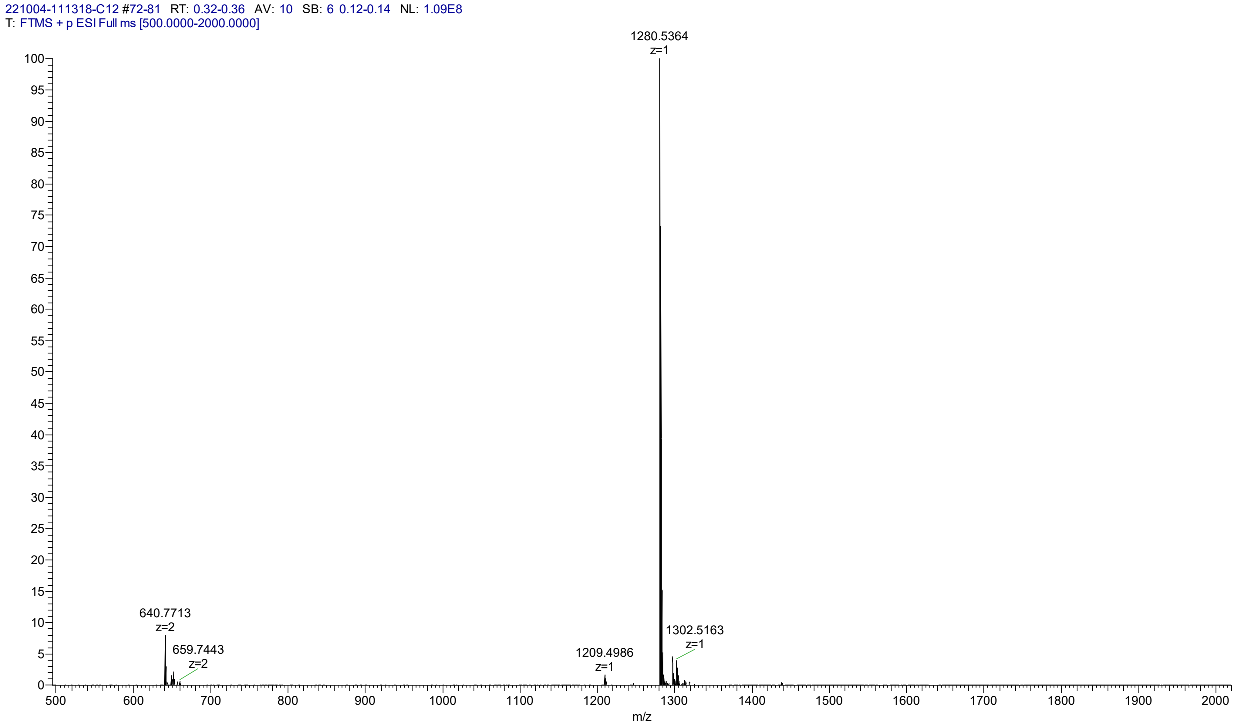

Supplementary Figure 102 Characterization of BSM C-capped 12-mer by analytical HPLC and high resolution MS. mAU refers to mini Arbitrary Unit.

TMB-3C retention time: 8.429 min calculated $[M+H]^+$: 603.15 Observed $[M+H]^+$: 604.1449

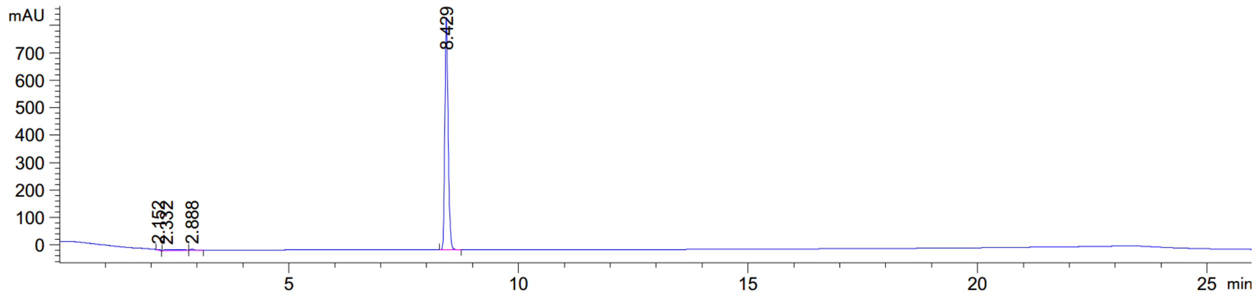

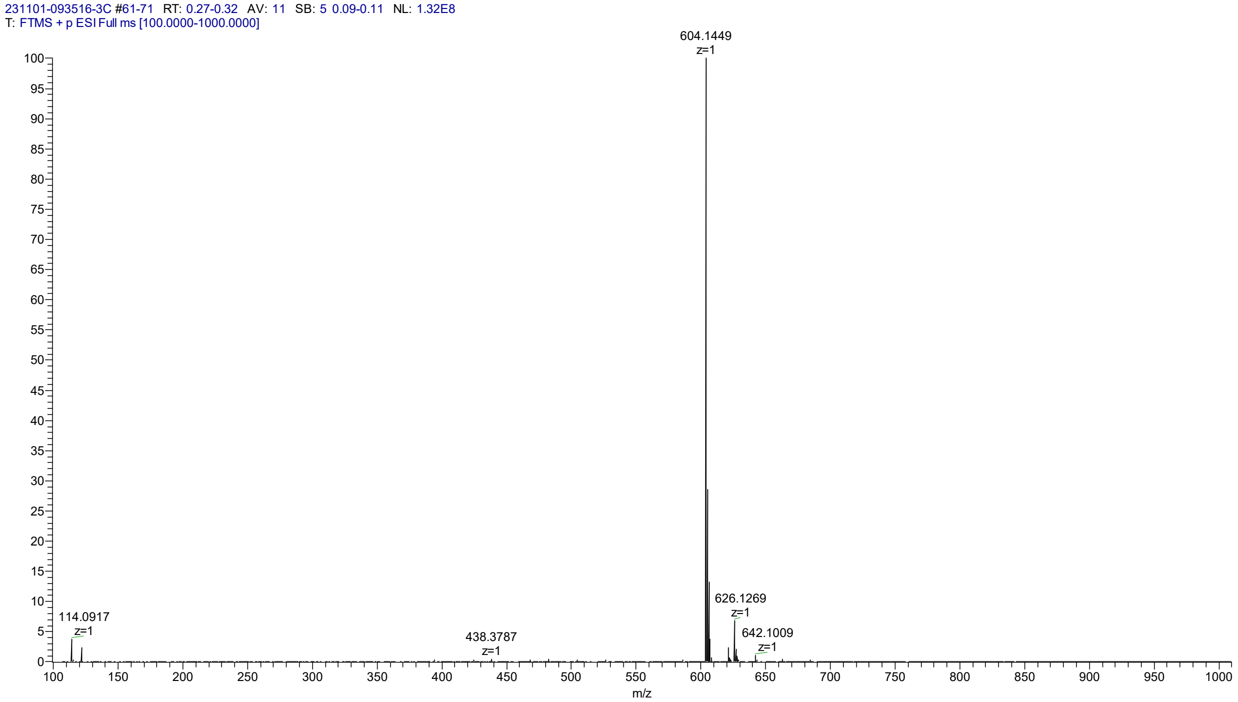

Supplementary Figure 103 Characterization of TMB-3C by analytical HPLC and high resolution MS. mAU refers to mini Arbitrary Unit.

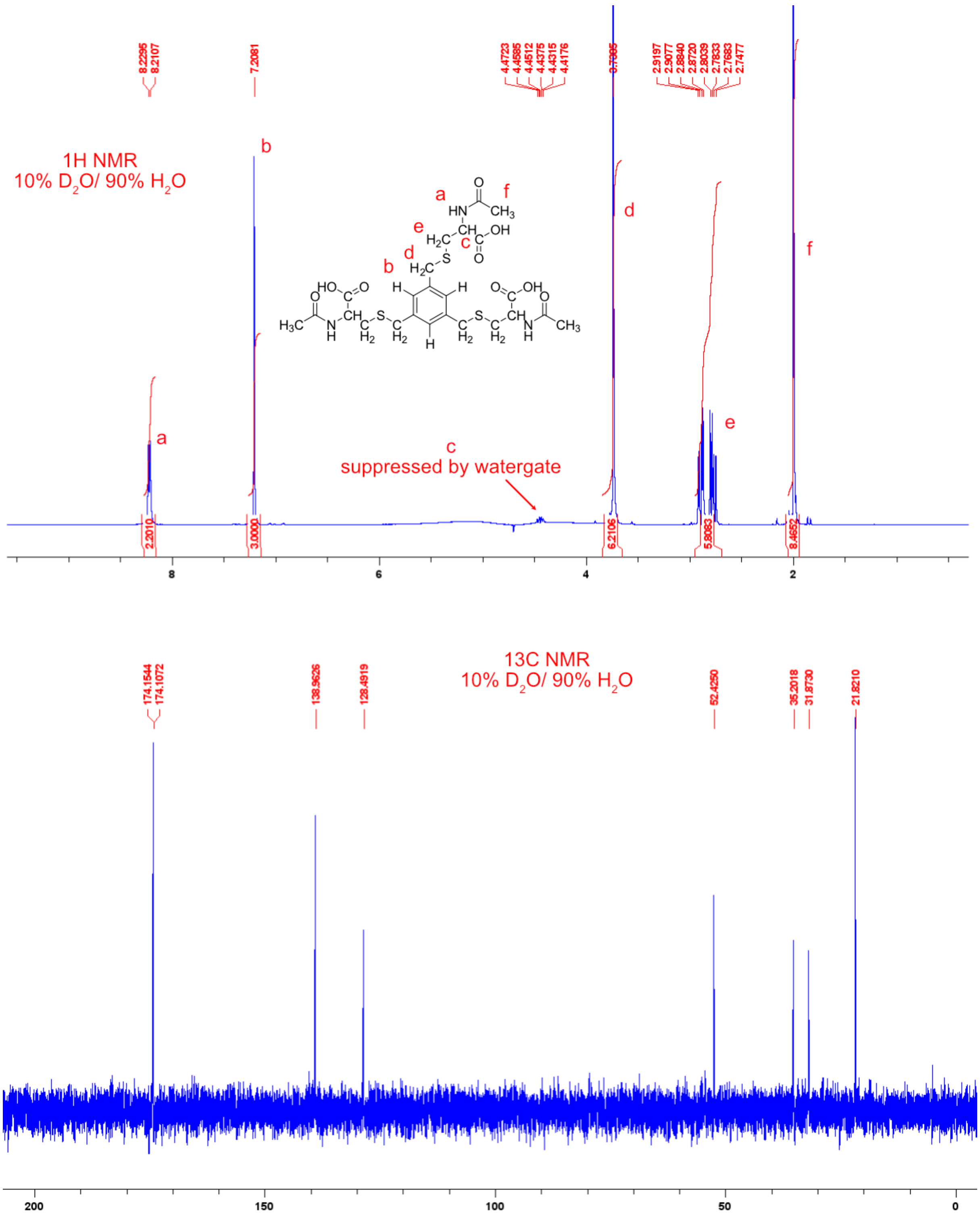

Supplementary Figure 104 Characterization of TMB-3C by ^{1}H and ^{13}C NMR spectra.

Supplementary References

1 Wan, W.-Y. & Milner-White, E. J. A natural grouping of motifs with an aspartate or asparagine residue forming two hydrogen bonds to residues ahead in sequence: Their occurrence at α-helical N termini and in other situations. *J. Mol. Biol.* **286**, 1633-1649, doi:10.1006/jmbi.1999.2552 (1999).

2 Kabsch, W. & Sander, C. Dictionary of protein secondary structure: pattern recognition of hydrogen-bonded and geometrical features. *Biopolymers* **22**, 2577-2637, doi:10.1002/bip.360221211 (1983).

3 Wallimann, P., Kennedy, R. J., Miller, J. S., Shalongo, W. & Kemp, D. S. Dual wavelength parametric test of two-state models for circular dichroism spectra of helical polypeptides: Anomalous dichroic properties of alanine-rich peptides. *J. Am. Chem. Soc.* **125**, 1203-1220, doi:10.1021/ja0275360 (2003).

4 Greenfield, N. J. Using circular dichroism spectra to estimate protein secondary structure. *Nat. Protoc.* **1**, 2876-2890, doi:10.1038/nprot.2006.202 (2006).

5 Zerbe, O. & Bader, R. Peptide NMR. https://www.chem.uzh.ch/zerbe/PeptidNMR.pdf

6 Nguyen, D., Mayne, L., Phillips, M. C. & Walter Englander, S. Reference Parameters for Protein Hydrogen Exchange Rates. *J. Am. Soc. Mass Spectrom* **29**, 1936-1939, doi:10.1007/s13361-018-2021-z (2018).

7 Wang, D., Lu, M. & Arora, P. S. Inhibition of HIV-1 fusion by hydrogen-bond-surrogate-based alpha helices. *Angew. Chem. Int. Edit.* **47**, 1879-1882, doi:10.1002/anie.200704227 (2008).

8 Wang, D., Liao, W. & Arora, P. S. Enhanced metabolic stability and protein-binding properties of artificial alpha helices derived from a hydrogen-bond surrogate: application to Bcl-xL. *Angew. Chem. Int. Edit.* **44**, 6525-6529, doi:10.1002/anie.200501603 (2005).

9 Henchey, L. K. *et al.* Inhibition of Hypoxia Inducible Factor 1-Transcription Coactivator Interaction by a Hydrogen Bond Surrogate alpha-Helix. *J. Am. Chem. Soc.* **132**, 941-+, doi:10.1021/ja9082864 (2010).

10 Kushal, S. *et al.* Protein domain mimetics as in vivo modulators of hypoxia-inducible factor signaling. *P. Natl. Acad. Sci. USA* **110**, 15602-15607, doi:10.1073/pnas.1312473110 (2013).

11 Patgiri, A., Yadav, K. K., Arora, P. S. & Bar-Sagi, D. An orthosteric inhibitor of the Ras-Sos interaction. *Nat. Chem. Biol.* **7**, 585-587, doi:10.1038/Nchembio.612 (2011).

12 Zhao, H. *et al.* Crosslinked Aspartic Acids as Helix-Nucleating Templates. *Angew. Chem. Int. Edit.* **55**, 12088-12093, doi:10.1002/anie.201606833 (2016).
