# Supplementary material for: Bioinformatics leading to conveniently accessible, helix enforcing, bicyclic ASX motif mimics (BAMMs)
Source: Nat Commun. 2024 May 17;15:4217. doi: 10.1038/s41467-024-48323-z (PMC11101637; doi:10.1038/s41467-024-48323-z)
Supplement: Supplementary file 3 — Description of Additional Supplementary Files [file 41467_2024_48323_MOESM3_ESM.pdf]

Description of Additional Supplementary Files

Supplementary Data 1:

(nonredundant ASX motifs):

Unique ASX motifs mined from PDB and their location, sequence, secondary structure and dihedral angle information.

Supplementary Data 2:

(helical N-cap ASX motifs)

Unique ASX motifs served as helical N-caps and their location, sequence, secondary structure and dihedral angle information.

Supplementary Data 3:

(hydrophobic patterns of helical N-cap ASX motifs)

Helical N-capped ASX motifs with hydrophobic residues at N' and N4, and their location, sequence, secondary structure, dihedral angle and cross-turn hydrophobic interaction information.
